# Fully Screen-Printed and Gentle-to-Skin Wet ECG Electrodes with Compact Wireless Readout for Cardiac Diagnosis and Remote Monitoring

**DOI:** 10.1021/acsnano.3c12477

**Published:** 2024-03-25

**Authors:** Sakandar Rauf, Rana M. Bilal, Jiajun Li, Mohammad Vaseem, Adeel N. Ahmad, Atif Shamim

**Affiliations:** † Electrical and Computer Engineering, CEMSE, King Abdullah University of Science and Technology (KAUST), Thuwal 23955-6900, Kingdom of Saudi Arabia; ‡ School of Medicine, University of Nottingham, Nottingham NG7 2UH, United Kingdom

**Keywords:** flexible electrode, ECG, wearable, silver nanowire ink, screen-printing, single-lead ECG, 12-lead ECG, antenna-on-package

## Abstract

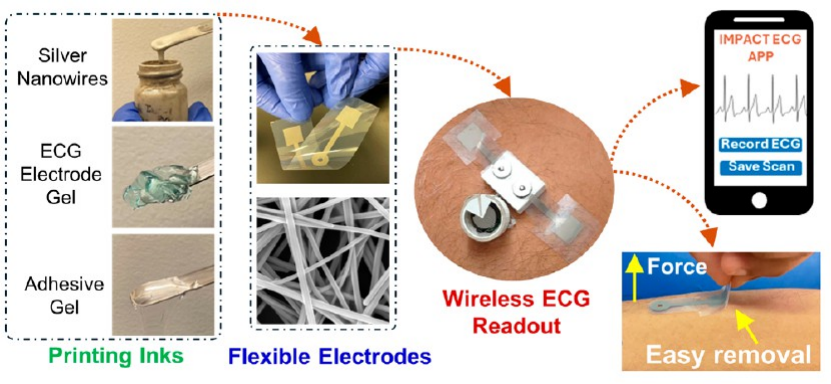

Recent advances in electrocardiogram (ECG) diagnosis and monitoring have triggered a demand for smart and wearable ECG electrodes and readout systems. Here, we report the development of a fully screen-printed gentle-to-skin wet ECG electrode integrated with a scaled-down printed circuit board (PCB) packaged inside a 3D-printed antenna-on-package (AoP). All three components of the wet ECG electrode (i.e., silver nanowire-based conductive part, electrode gel, and adhesive gel) are screen-printed on a flexible plastic substrate and only require 265 times less metal for the conductive part and 176 times less ECG electrode gel than the standard commercial wet ECG electrodes. In addition, our electrically small AoP achieved a maximum read range of 142 m and offers a 4 times larger wireless communication range than the typical commercial chip antenna. The adult volunteers’ study results indicated that our system recorded ECG data that correlated well with data from a commercial ECG system and electrodes. Furthermore, in the context of a 12-lead ECG diagnostic system, the fully printed wet ECG electrodes demonstrated a performance similar to that of commercially available wet ECG electrodes while being gentle on the skin. This was confirmed through a blind review method by two cardiology consultants and one family medicine consultant, validating the consistency of the diagnostic information obtained from both electrodes. In conclusion, these findings highlight the potential of fully screen-printed wet ECG electrodes for both monitoring and diagnostic purposes. These electrodes could serve as potential candidates for clinical practice, and the screen-printing method has the capability to facilitate industrial mass production.

According to the World Heart Federation, cardiovascular diseases caused 20.5 million deaths in 2021, and their incidence is rising worldwide, impacting over 500 million people globally.^1^ Therefore, there is a pressing need to develop tools for early diagnosis and therapies for personalized health monitoring. The electrocardiogram (ECG) is frequently used for diagnosing cardiovascular diseases and monitoring the health of the human heart. The 12-lead ECG system is used for diagnostic purposes, whereas 1, 3, or 6 leads are used for monitoring the heart rhythm.^2^ Recent advances have led to wearable ECG systems for monitoring the heart and quickly checking its health,^3^ which can reduce the frequency of hospital visits by users. In hospitals, ECG systems commonly use commercially available disposable Ag/AgCl wet ECG electrodes or reusable vacuum cup electrodes. Disposable single-use Ag/AgCl wet ECG electrodes cost around 0.2–0.9 USD per electrode,^4^ while reusable vacuum cup electrodes are uncomfotable and result in sore skin and rashes. In addition vacuum cup electrodes require decontamination and can spread antibiotic-resistant pathogens even after decontamination or due to inadequate decontamination.^5^ Therefore, disposable Ag/AgCl wet ECG electrodes are preferred over reusable vacuum cup electrodes. However, disposable Ag/AgCl wet ECG electrodes remain expensive for a broader community, such as the least developed or developing countries. To date, vacuum cup electrodes are still in use in many parts of the world. Additionally, the price per Ag/AgCl wet ECG electrode (ranging from 0.2 to 0.9 USD per electrode) increases with gentle-to-skin and easy to remove features, elevating the overall usage cost of ECG systems to a point where they become unaffordable. This can result in financial burdens and discomfort, which can discourage their everyday usage. Innovative approaches to fabrication that have the potential to decrease the cost of disposable wet ECG electrodes could increase the widespread use of these electrodes worldwide. On the other hand, wearable ECG systems for personal health monitoring use commercially available wet ECG electrodes or custom dry electrodes. Commercially available dry electrodes need some pressure in the form of a wearable belt to improve contact with the skin to acquire a good ECG signal.^6−8^ Applying or removing these electrodes causes significant discomfort to the patient and results in sore skin and rashes for patients with sensitive skin.^9,10^

Depending on the type of application, dry and wet ECG electrodes have advantages and disadvantages. For example, dry electrodes are suitable when it comes to long-term ECG monitoring during exercise and show comparable performance to the commercial wet ECG electrodes as soon as perspiration fills the skin–electrode gap.^11−13^ Whereas the wet ECG electrodes (commonly used commercial disposable ECG electrodes) perform better than the dry electrodes in the clinical settings and, hence, are used routinely in clinical practice to date. Numerous efforts have been made to fabricate dry ECG electrodes using various functional materials, such as silver nanowires (AgNWs) deposited on or embedded in poly(dimethylsiloxane) (PDMS),^14−17^ a zwitterionic polymer brush coated on Au/PDMS,^18^ graphene,^19−22^ carbon nanotube composites,^23^ conductive textiles,^24,25^ nanomaterial–polymer composites,^26^ 3D electrodes,^27,28^ poly(ionic liquid) nanofiber membranes,^29^ and conductive polymers blended with other materials for improved flexibility and adhesiveness.^30,31^ A common challenge is that the estimated production cost of these electrodes is higher than that of commercially available wet ECG electrodes owing to the inclusion of multiple complex manufacturing processes such as spin-coating, multiple lithography processes, casting, coating, electrode connection via snap connectors or soldering of the wires, and manual handling. Thus, many potential ECG electrodes that have been fabricated at the laboratory scale are unsuitable for large-scale production. This means that hospitals still have to use expensive Ag/AgCl wet ECG electrodes or uncomfortable vacuum cup electrodes for clinical diagnosis.

Electronics printing methods such as roll-to-roll and screen-printing allow for the large-area, high-volume, and low-cost production of flexible electronic components.^32^ Dry ECG electrodes have been screen-printed from conductive inks such as silver nanowires (AgNWs), PEDOT:PSS, and carbon.^13,33−35^ However, these efforts were mainly focused on the development of dry ECG electrodes for ECG monitoring purpose. To the best of our knowledge, the screen-printing of wet ECG electrodes and their testing on volunteers in a clinical setting for ECG diagnosis and monitoring purposes have not been reported.

Recent interest in telehealth, precision medicine, and the Internet of Things or Internet of Medical Things^36^ has led to an emerging trend of sending health data to smartphone apps for continuous monitoring and remote diagnosis.^36,37^ Such applications require an antenna for wireless communication, which should provide a decent gain across the whole bandwidth used by communication protocols and be easily integrated with a wearable ECG system. The antenna-on-package (AoP)^38^ fully utilizes the system’s volume and expands the design freedom to provide better performance. An AoP also serves as the packaging/enclosure, which saves considerable space, weight, and cost compared to other antenna design options in the literature. The miniaturization of ECG devices is critical for practical application by improving their mobility and ease of use. For instance, typical ECG devices for neonatal monitoring use long wires that connect electrodes to a readout device on the side of the infant’s bed (i.e., not worn). This reduces access and mobility for parental care as well as for clinical processes. In contrast, a small and lightweight wearable device would be far more convenient in such cases.^3^

Here, we report fully screen-printed wet ECG electrodes integrated with a miniaturized ECG readout device consisting of a custom-designed PCB packaged inside a 3D-printed AoP (Figure 1). The electrodes provide soft and gentle contact with the human skin while acquiring a high-quality ECG signal on both a 12-lead ECG diagnosis system and miniaturized ECG monitoring readout device. The readout device is user-friendly and has a better communication range than that of commercial chip antennas. In the case of fully screen-printed wet ECG electrodes, all three components (i.e., AgNW-based conductive part, electrode gel, and adhesive gel) are printed onto a single substrate. We also developed an atypical electrode–device interface that connects the electrodes to the wireless readout device in a plug-and-play manner without the need for snap connectors or conductive epoxy for soldering.

## Results and Discussion

**Figure 1:**
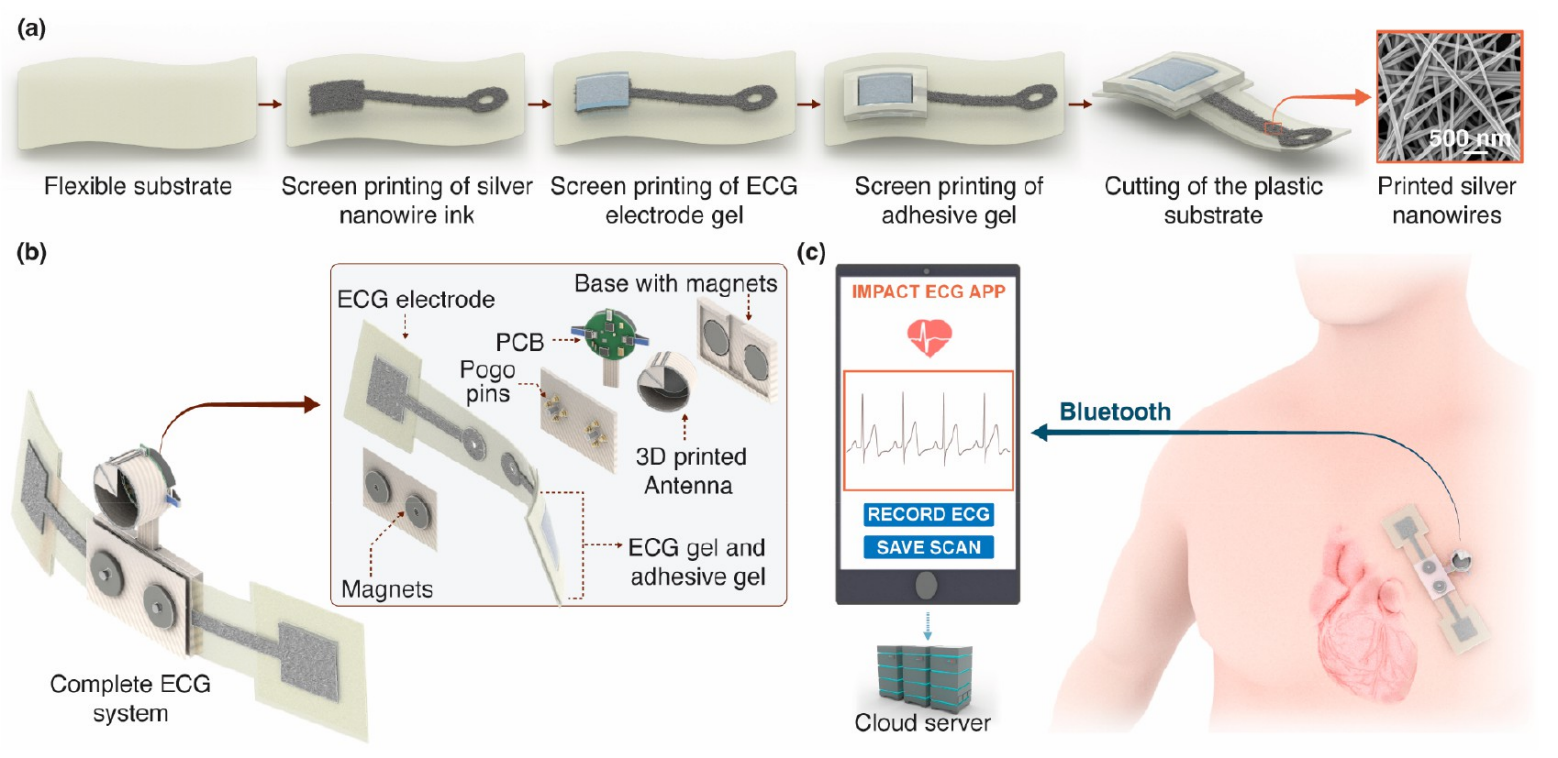
Schematic of the developed ECG system. (a) Fabrication process of the ECG electrodes. (b) Complete ECG system comprising the ECG electrodes, electrode interface, and PCB packed inside a 3D-printed AoP. The inset shows the details of each component. (c) Operation of the ECG system, which acquires the ECG signal and sends data to a dedicated server via a smartphone app. Printed with permission from H. H. Hwang, scientific illustrator. The illustration was created after receiving input from one of the authors and using real pictures of the ECG system. One of the authors provided a rough sketch of the illustration and also labeled all the components of the figure.

Figure 1 illustrates the developed ECG system. Figure 1a shows the fabrication process of the wet ECG electrodes. The AgNWs ensure that the electrode has a high conductivity and that it can acquire a high-quality ECG signal. The electrode gel reduces electrical impedance at the point of contact with the skin and results in a high-quality ECG signal. The biocompatible adhesive gel creates soft adhesion and easy release from the skin. The ECG electrodes are gentle on the skin, easier to remove than typical commercial ECG electrodes, and avoid skin damage or irritation. The ECG electrodes can be used in 12-lead ECG systems and wearable wireless ECG readout devices for health monitoring. We used screen-printing to fabricate the ECG electrodes, which are low in cost and can be scaled up for mass production. Figure 1b shows the miniaturized wireless readout device, which was designed to complement the ECG electrodes. The custom readout device integrates a custom 3D-printed AoP. Figure 1c shows the operation of the complete ECG system, which is compact, wearable, and wireless. It has a communication range that is 4 times greater in the direction of maximum radiation than that of a commercial chip antenna.

### Electrodes

**Figure 2:**
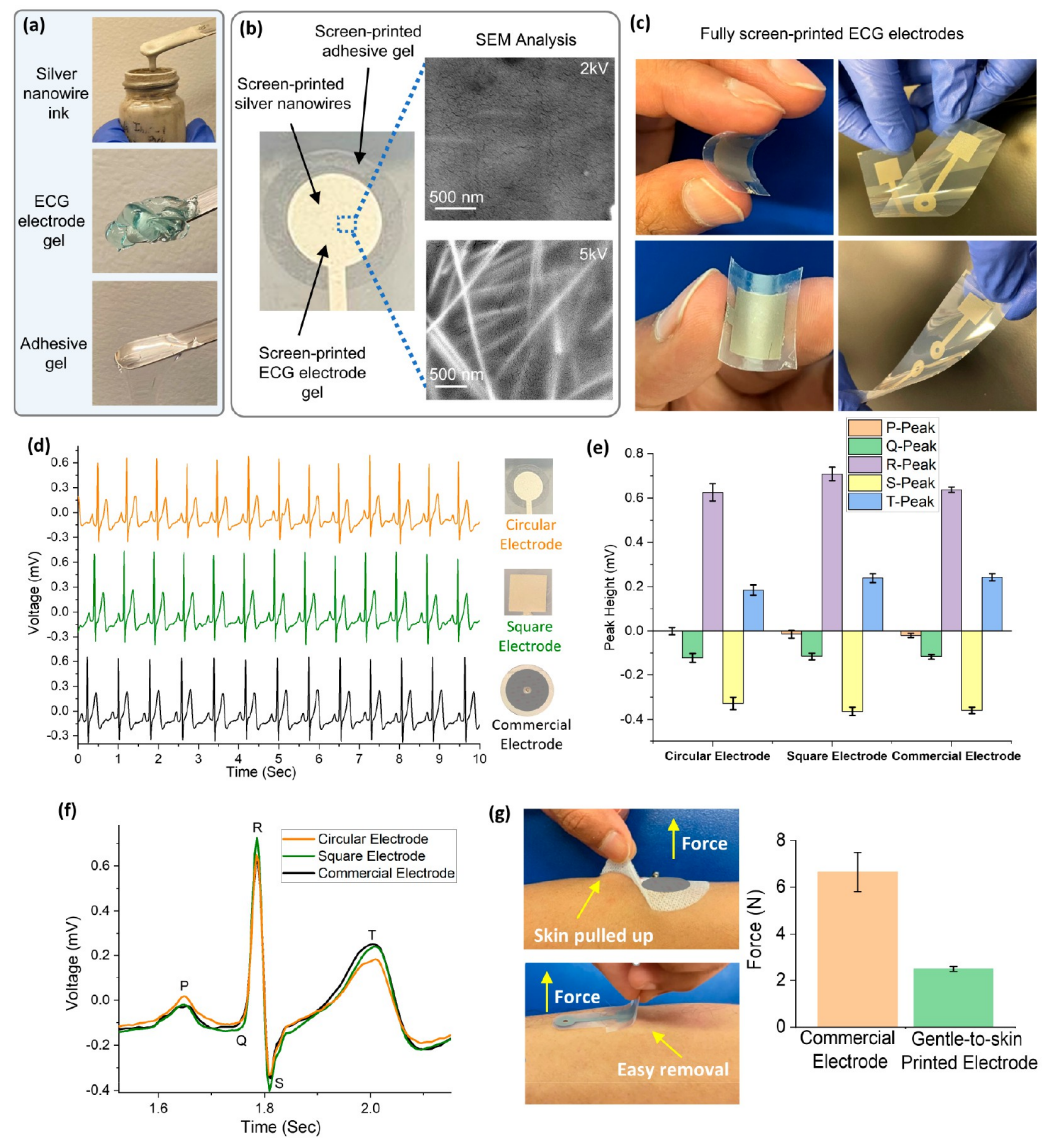
Fabrication and characterization of ECG electrodes. (a) Inks used for screen-printing of electrodes. (b) Electrode comprising conductive AgNWs, ECG electrode gel, and adhesive gel screen-printed on a plastic substrate. (c) Flexible fully screen-printed ECG electrodes. (d) ECG signal from an adult volunteer using pairs of screen-printed square and circular electrodes as well as commercial ECG electrodes. The ECG electrodes were placed on the volunteer’s chest at a center-to-center distance of 8 cm. (e) Characteristic peak amplitudes of the ECG signals (P, Q, R, S, and T) from the electrodes. The error bars show the standard deviation of peak amplitudes calculated for a 10 s ECG with each type of electrode. (f) Normal sinus rhythm ECG wave with characteristic ECG peaks (P, Q, R, S, and T) for each electrode type. (g) Removal of the screen-printed ECG electrode and a commercially available ECG electrode (Supporting Information Videos 2 and 3). The bar graph illustrates the force required to remove the commercial ECG electrode and the gentle-to-skin-screen-printed ECG electrode. The measurements were conducted by using a universal testing machine (ZwickRoell Z0.5 TN) with a strain rate of 20 mm/min.

Commercially available wet ECG electrodes consist of three components: the conductive part (commonly silver/silver chloride) attached to a snap connector, the electrode gel on top of the electrode, and an adhesive on the sides so that the electrode sticks to the body. We screen-printed all three components and replaced the snap connector with a custom interface (Figure 1b). We fabricated fully screen-printed ECG electrodes that are gentle on the skin and compatible with our custom ECG readout device and existing 12-lead ECG diagnosis systems in hospitals (Figure 1a,b). Figure 2a,b shows the inks used to print the electrodes and a printed circular electrode. The thixotropic behavior of the AgNW ink, ECG electrode gel, and adhesive gel was determined by rheological studies, which confirmed that the required fluid behavior and viscosity for good printability were achieved (Figure S1). After the AgNWs were printed on the plastic substrate, the electrode gel was printed on top of the AgNWs, and the adhesive gel was printed on the sides of the substrate, as shown in Figure 2b. The scanning electron microscopy images show that the electrode gel completely covered the AgNWs. At a low acceleration voltage of 2 kV, only the top surface of the electrode gel is visible, and the AgNWs are not visible. The embedded AgNWs could be observed at an acceleration voltage of 5 kV. Figure 2c shows the flexibility of the screen-printed electrodes. To obtain an ECG signal quality comparable to that of commercial ECG electrodes, we evaluated different electrode sizes (Figure S2) and the number of printed layers of AgNW ink (Figures S3 and S4). Increasing the diameter of the electrode from 7 to 14 mm and printing three layers of AgNW ink with a sheet resistance of 0.72 ± 0.03 Ω/sq realized an acceptable ECG signal quality (Figures S2 and S3). Printing three layers of AgNW ink also realized an interfacial impedance close to that of commercial ECG electrodes and less than the impedances with one or two layers of AgNWs (Figure S4). Thus, ECG electrodes with three printed layers of AgNW ink were used in all subsequent measurements. To assess the effect of the electrode shape on the ECG signal quality, we tested circular electrodes with a diameter of 14 mm and square electrodes with similar surface areas (Figure 2d–f). All characteristics of ECG peaks (PQRST) appeared in all ECG signals, and the calculated peak heights (i.e., amplitudes) clearly show that the electrode shape did not significantly affect the ECG signal. However, the square electrodes acquired lower interfacial impedance (Figure S4) than the circular electrodes, and they performed similarly to commercial electrodes. A low interfacial impedance is essential for obtaining a reliable and sensitive ECG signal.^39^ Hence, the square electrodes were used in subsequent volunteer studies on single-lead wireless and 12-lead ECG systems. The screen-printed ECG electrodes are gentle on the skin because we used a biocompatible adhesive gel (high-tack silicone gel A4717) that sticks well to the skin (Supporting Information Video 1), which allows the electrodes to acquire a high-quality ECG signal while easily separating from the skin compared to commercial ECG electrodes (Supporting Information Videos 2 and 3). Figure 2g clearly shows that a volunteer’s skin was pulled up when a commercial ECG electrode was removed (Supporting Information Video 3), which causes discomfort and can result in skin rashes. However, our screen-printed electrodes are easy to remove and do not pull up the skin compared with commercial ECG electrodes (Supporting Information Video 2). In addition, we conducted a peel test to assess the ease of removing the printed ECG electrodes compared to the commercial ECG electrodes (Figures 2g and S5). The adhesion of the printed ECG electrode is sufficient to acquire the ECG signal, requiring approximately 2.5 times less force during the peel test, compared to the commercial ECG electrode. This demonstrates the gentle-to-skin and easy removal features of the printed ECG electrode. To assess the operational stability and reliability of the printed ECG electrodes, cyclic bending and twisting tests were conducted (Figure S6 and Supporting Information Video 4). It is evident that the ECG signal remained unaffected even after 50 000 bending cycles and 24 h of twisting, indicating the operational stability of the ECG electrodes.

Another interesting fact about screen-printed ECG electrodes is that these are lightweight and use less material than commercial wet ECG electrodes. On average, a printed ECG electrode uses 0.86 ± 0.05 mg of silver nanowires as a conductive part, which is 265 times less metal than the commercial wet ECG electrode (227.72 ± 2.48 mg of metal as a conductive part). In addition, the ECG electrode gel used in the screen-printed ECG electrode (2.57 ± 0.47 mg) is 176 times less than the commercial wet ECG electrode (453.73 ± 43.55 mg). Our screen-printing method is versatile, and AgNW ink can be printed on different substrates such as polyethylene terephthalate (PET), polyethylene naphthalate (PEN), and polyimide (PI) (Figure S7). The estimated cost of a single ECG electrode using the lab-based reagents and lab scale synthesis is 0.35 USD per electrode (Table S1) while using a PET substrate. The cost of the two most expensive chemicals (ethylene glycol and silver nitrate used in the synthesis of silver nanowire ink) if purchased at the industrial scale was found to be 10 times less than the lab scale (Sigma-Aldrich) chemicals (Table S1). Applying the similar estimate for all the chemicals and considering the mass production using the screen-printing industrial process, which is a well-established, automated, and high-throughput printing method, we anticipate that the cost of a single ECG electrode will be approximately 10 times lower (0.03 USD per electrode) or even lower further in a full-scale industrial process compared to the lab scale. Moreover, Table S2 illustrates the advantages and disadvantages of various techniques reported in the literature for fabricating ECG electrodes, comparing them to the screen-printing method, which provides a competitively low-cost option for mass manufacturing and fast prototyping compared to other techniques. Hence, the screen-printed electrodes show promise for low-cost manufacturing at an industrial scale while being gentle on the skin.

### Overview of ECG Monitoring System and Components

**Figure 3:**
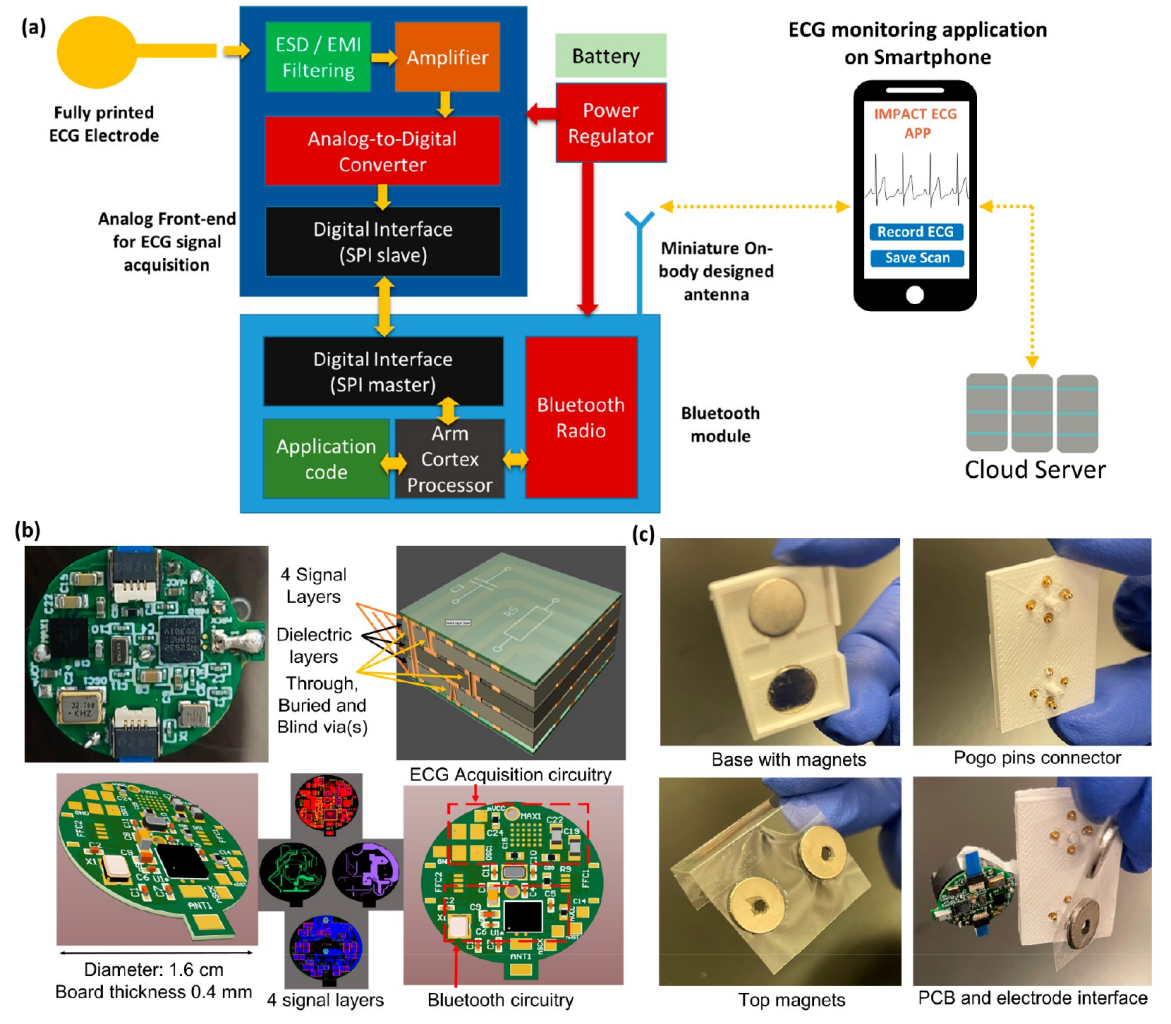
Overview of ECG system and components. (a) Conceptual diagram of the ECG monitoring system. (b) PCB of wireless ECG readout device comprising four signal layers on a fiberglass epoxy (FR4) board with a thickness of 0.4 mm and containing through, buried, and blind vias to interconnect signal layers. (c) Interface connecting the ECG electrodes with the PCB without the need for traditional snap connectors. The electrodes are placed face down on the pogo pins. A small pillar in the center of the pogo pins aligns the electrodes, which make pressure contact due to the presence of the base and top magnets.

As a complement to the screen-printed wet ECG electrodes, we also developed a complete end-to-end ECG monitoring system comprising a miniaturized wireless readout device, a smartphone app, and cloud server software for storage and later analysis of collected ECG data (Figures 3 and S8). Figure 3a depicts the complete ECG system. The ECG signal is acquired via screen-printed electrodes attached to the human body. Then, the readout device filters the signal for noise removal and amplifies and digitizes the signal for Bluetooth low energy (BLE) transmission to the smartphone app. The smartphone app presents the ECG waveform to the user and sends it to the cloud server for storage and possible postprocessing in the future, such as with artificial intelligence.^40^Figures 3b and S9 show the detailed design of the printed circuit board (PCB) inside the readout device on a four-layer fiberglass epoxy (FR4) substrate. This system uses pogo pins and a magnetic press connection (Figures 1b and 3c) for a seamless interface with the electrodes. The Methods section contains further details on the design.

### Antenna-on-Package

**Figure 4:**
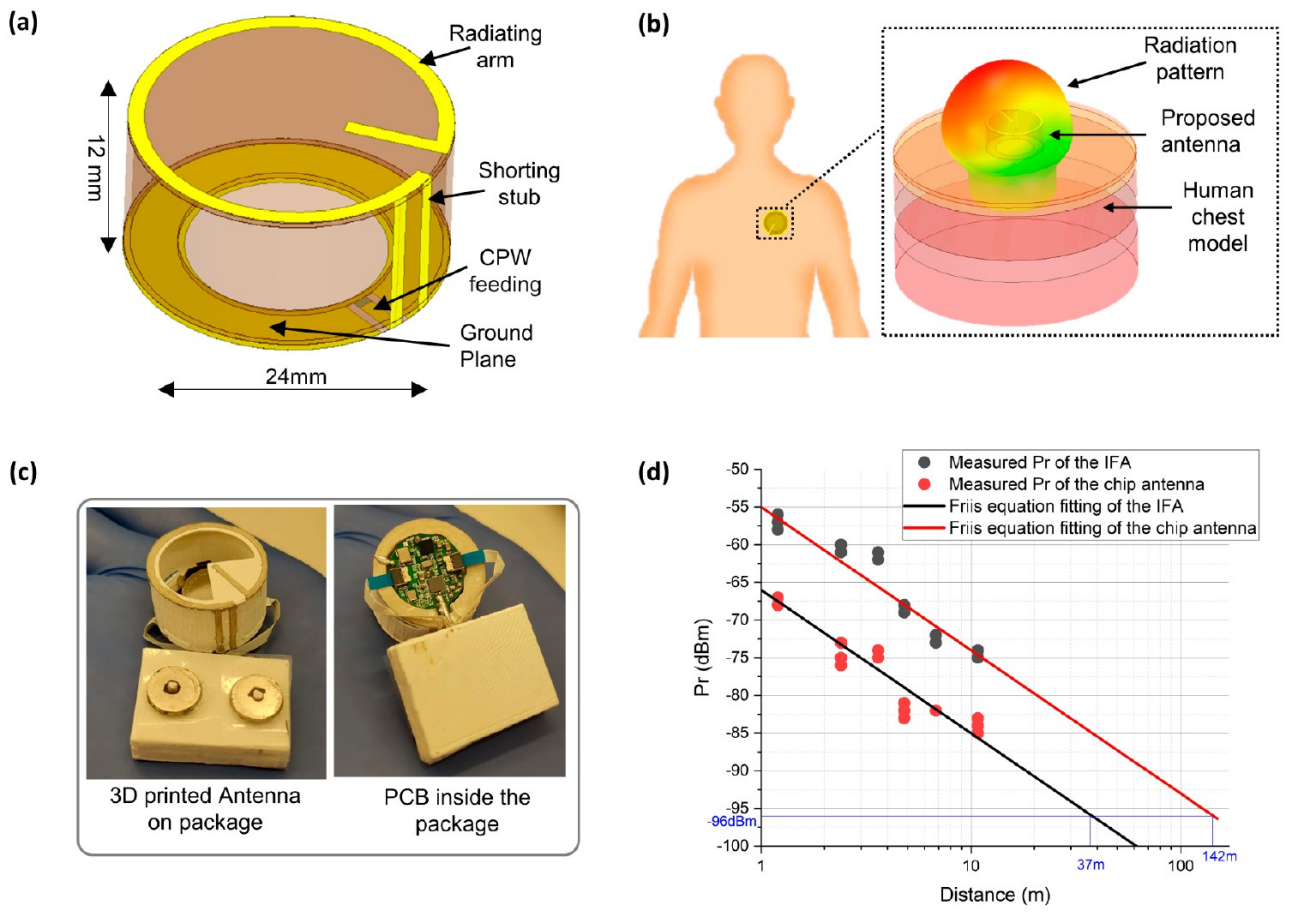
3D-printed electrically small antenna-on-package (AoP). (a) Model of the proposed antenna. (b) Antenna on human body and its radiation pattern. (c) Fabricated antenna integrated with the circuits. (d) Active test of the antenna: received power (Pr) against the communication distance.

Our ECG readout device uses an electrically small AoP to overcome the challenges of achieving adequate gain and bandwidth within a limited space. As shown in Figure 4, the AoP design endows the dual functions of radiation and protection, and it utilizes the available space to mitigate size limitations. The AoP was specifically designed for on-body application, and its outstanding on-body performance was verified via simulation with the commercial electromagnetic simulation software High-Frequency Structure Simulator (HFSS)^41^ and measurements without the ECG PCB. Figure 4a shows the 3D model detailing different components of the AoP. Figure 4b shows the detailed simulation model of the AoP and human chest, which comprised a skin layer (2 mm), fat layer (4 mm), muscle layer (20 mm), bone layer (10 mm), and heart layer (30 mm).^42^Figure 4c shows the fabricated AoP and the PCB within. The designed AoP is easy to integrate with the ECG PCB and the electrode interface. The RF output of the PCB and coplanar waveguide (CPW) of the AoP are connected by conductive epoxy. The front side of the AoP is open to allow for battery replacement. A ground plane is located underneath the radiator to isolate the antenna from the human body, to reduce the degeneration owing to the loss from the tissue, and to protect the patient from electromagnetic radiation. We compared the on-body radiation of the designed AoP and a chip antenna with the same PCB by active testing on the volunteer’s chest (Figure 4d). Based on the Friis equation,^43^ we calculated that the maximum read range of the AoP is 142 m. In addition, when the input power is set to 0 dBm, which is the output power of the Bluetooth transceiver chip nRf52832, the maximum average specific absorption rate (SAR) was found to be 0.0162 W/kg in 10 g—less than the EU standard of 2 W/kg. Additionally, the SAR was determined to be 0.0379 W/kg in 1 g, which is below the USA standard of 1.6 W/kg. Also, the on-body gain was found to be 0.3 dB. These results demonstrate that the AoP provides a stable data link when communicating with a smartphone and in larger-scale monitoring scenarios, such as transmitting data to the receiving station in a big hall. It is estimated that the cost per wearable ECG system, when producing 1000 units, is ≅20 USD (Table S1). Thus, the developed ECG system is low cost and provides outstanding on-body performance and better communication than a commercial chip antenna.

### Adult Volunteer Study

**Figure 5:**
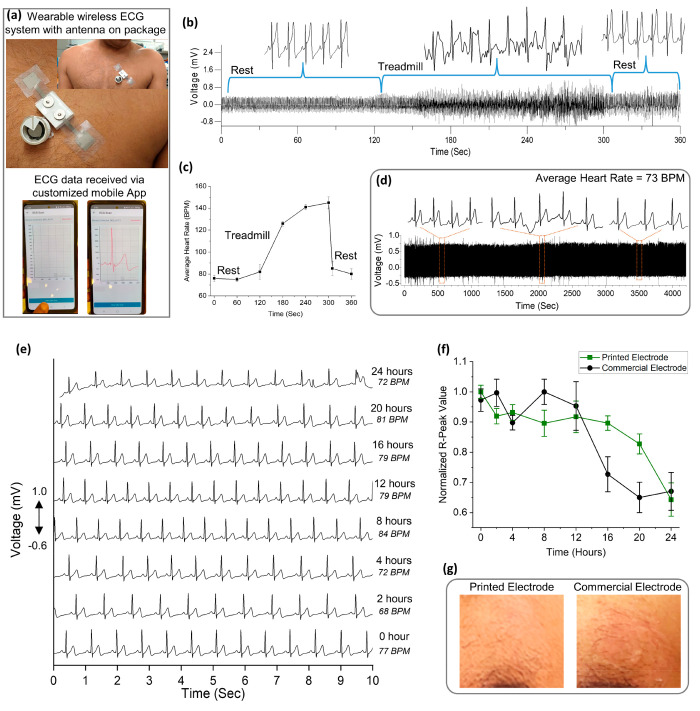
Adult volunteer single-lead ECG study. (a) Wearable ECG system attached to the chest of a volunteer and snapshots from the mobile app. (b) ECG data obtained from the volunteer at rest, exercise, and rest state. (c) Heart rate obtained using the data from b. The heart rate was calculated in the 10 s intervals between the rest, exercise, and rest. (d) ECG data from volunteer sitting in a work environment and wearing the ECG system for more than 1 h. (e) Long-term wearability of the printed wet ECG electrode. (f) Normalized R-peak values calculated for the printed wet ECG electrode and commercial ECG electrode. (g) Pictures taken after the removal of the printed wet ECG electrode and the commercial ECG electrode following 24 h of wear.

Finally, we conducted ECG measurements with adult volunteers to validate the practical applicability of our fully screen-printed wet ECG electrodes and readout device. All ECG electrodes were tested on adult volunteers, and the data were compared to those of commercially available Ag/AgCl wet ECG electrodes. Figure 5a shows the complete ECG system attached to the body of a volunteer. A customized smartphone app collected the ECG data (Supporting Information Video 5). We initially evaluated the wearable ECG monitor in various scenarios, such as rest–exercise–rest, and over extended measurement intervals to assess its suitability for daily use. Figure 5b shows the ECG data from a volunteer wearing the ECG system during the rest–exercise–rest scenario. The volunteer started exercising on a treadmill for 2 min after a rest period, followed by 1 min of rest. It is evident that the wearable ECG system accurately measures changes in the ECG during these scenarios (Figure 5b). The volunteer’s heart rate (Figure 5c) increased from 76 BPM during the resting state to a peak of 145 BPM during exercise and then returned to 80 BPM during the subsequent resting state. It is important to note that as the volunteer began walking and running on the treadmill, the noise in the ECG signal increased (Figure 5b), primarily affecting the P and T peaks. However, the R peaks remained clearly visible, allowing for accurate heart rate measurements. This phenomenon occurs because, with the increase in speed (from rest to walking and running), the muscle movements generate stronger electromyography (EMG) signals compared to the ECG signal, leading to baseline drift.^44−46^ Other factors contributing to the ECG baseline drift are motion artifacts and breathing during the exercise.^44−46^

To validate the accuracy of our system, we also utilized a commercially available single-lead wireless ECG system (Getwellue), which also exhibited a baseline drift trend (Figure S10) similar to that of our system. This confirmed that the baseline drift was not an artifact of our electrodes or ECG readout. Overall, the signal quality was adequate to measure the heart rate of the volunteer during different scenarios. For long-term measurements, Figure 5d shows the ECG signal from a volunteer for more than 1 h while sitting in an office environment. Minor fluctuations in the ECG signal were observed; however, a high-quality ECG signal was obtained during this period where all ECG peaks (PQRST) can be identified clearly with an average heart rate of 73 BPM.

Additionally, to compare the long-term usage and wearability with the commercial wet ECG electrodes, both the printed wet ECG electrodes and commercial wet ECG electrodes were worn for 24 h. The volunteer followed a normal daily routine, including daytime activities and sleep, throughout this period. Figures 5e and S11 display the ECG readings from both electrodes measured during the 24 h period. Figure 5f illustrates the comparison of R-peak values in both cases over time. It is evident that after 16 h of use, the performance of the commercial wet ECG electrode significantly degraded (approximately 30%) compared to the initial measurement at 0 h. In contrast, the screen-printed wet ECG electrode demonstrated better performance, maintaining functionality until 20 h (approximately 15% decrease in signal) compared to the wet ECG electrode. Both electrodes showed a decrease in the R-peak value of approximately 35% after 24 h of wearability. This decline is attributed to the drying of the ECG electrode gel in both cases, caused by water vaporization from the hydrogels, leading to signal decay and noise in the ECG signal.^31^ However, the R-peak signal loss was slower in the case of printed wet ECG electrodes. Additionally, screen-printed wet ECG electrodes showed no signs of irritation/rashes on the skin after 24 h, unlike the commercial wet ECG electrodes (Figure 5g), indicating that these electrodes could be useful for monitoring purposes.

The printed wet ECG electrodes were also tested for sweat tolerance by applying artificial sweat on top of the electrodes for 2 to 24 h. Figure S12 shows that the addition of sweat has a minor effect on the electrode performance for the first 8 h. After 16 h, a decrease of approximately 20% in the R-peak value was observed compared to the sample without treatment with sweat. Nonetheless, even after 16 h of exposure to artificial sweat, the characteristic ECG peaks (P, Q, R, S, and T) can still be clearly identified, and the heart rate can be measured. After 24 h, the conductive part of the electrode (printed silver nanowires) was detached from the plastic substrate, resulting in no further acquisition of ECG data. The decrease in the ECG signal is mainly due to the removal of the ECG electrode gel, which is soluble in water and dissolved in artificial sweat. However, the electrode’s consistent signal is attributed to the mixture of electrolytes from sweat and residual ECG electrode gel on the electrodes. Even after the removal of sweat, the electrodes still appear wet, maintaining electrical impedance at the point of contact with the skin and resulting in an acceptable ECG signal.

**Figure 6:**
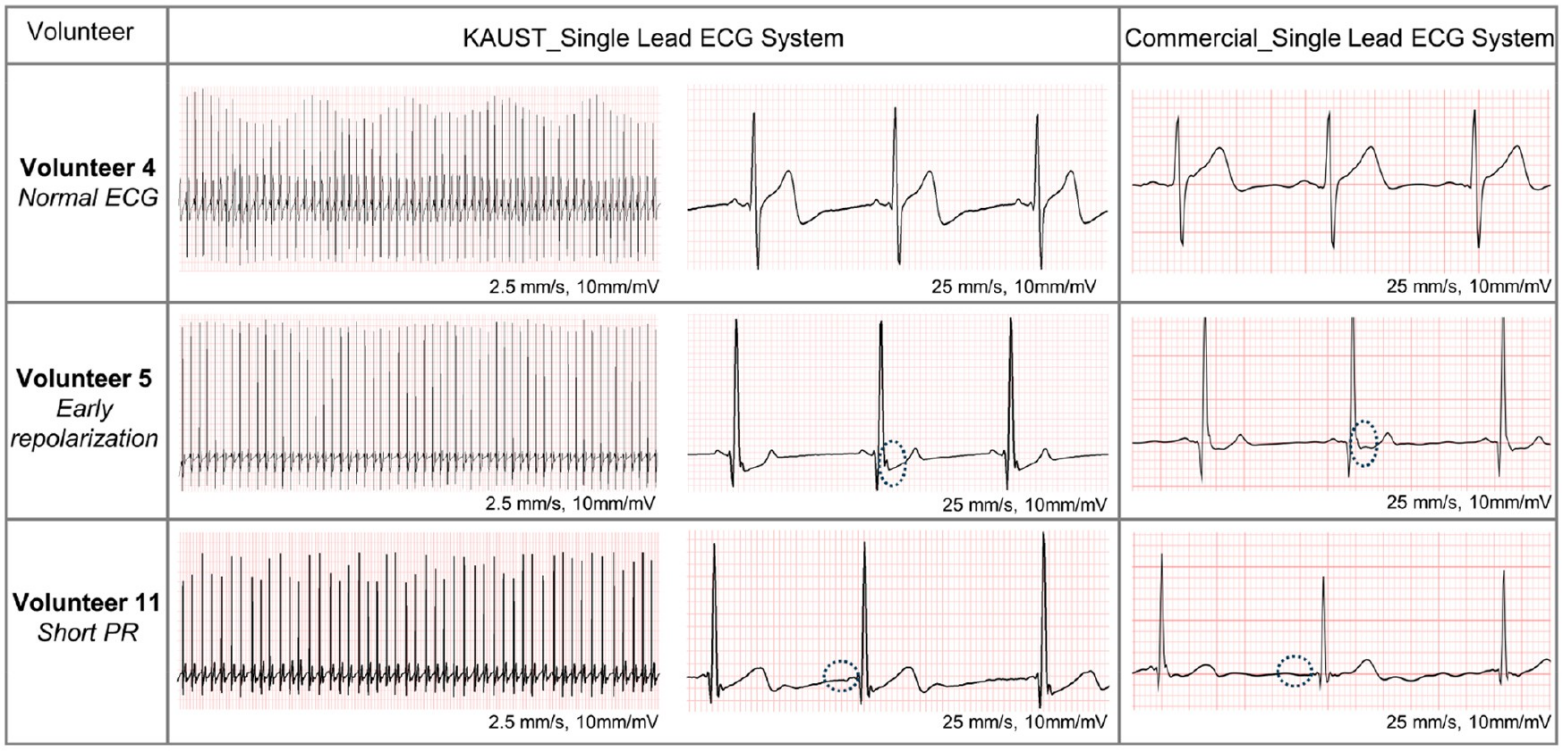
KAUST single-lead and commercial single-lead ECG comparisons between different volunteers. The ECG data from three volunteers were selected to show the normal ECG and detection of abnormalities such as early repolarization and short PR in the ECG. The abnormalities of early repolarization and short PR in the ECG are pointed out by dotted circles.

To highlight the potential of our wearable ECG system for diagnostic applications, we conducted a study with adult volunteers encompassing 20 individuals from diverse ethnic backgrounds, genders, and age groups (20–50 years). On each volunteer, four ECG measurements were performed. We conducted two ECG measurements using our single-lead ECG system, equipped with gentle-to-skin fully screen-printed electrodes, and a commercially available single-lead ECG system with commercially available disposable Ag/AgCl wet ECG electrodes. The remaining two measurements were performed using a hospital 12-lead ECG system utilizing gentle-to-skin screen-printed electrodes and commercially available disposable Ag/AgCl wet ECG electrodes. All the data were reviewed by two cardiology consultants and one family medicine consultant in a blinded review method (Figures S13–S32). Figure 6 compares the data for only three different volunteers, which show normal and abnormal ECG findings. To confirm that our system was accurate, we also used a commercially available single-lead wireless ECG system (Getwellue), which gave similar results (Figure 6) and confirmed that this was not an artifact of our electrodes or the ECG system. The ECG data of our ECG system and the commercial single-lead ECG system showed excellent correlation for all volunteers, showing all characteristic ECG peaks (PQRST complex; Figures S13–S32). The ECGs of volunteers 1–4 were identified as normal. However, in the case of male volunteer 5 (Figure 6), early repolarization (ER) was observed, characterized by J waves or J-point elevation,^47^ which is an electrocardiographic abnormality. ER is generally considered a normal variant, but recent studies have highlighted that its presence may increase the risk of death from cardiac arrhythmias.^47,48^ The ECG data from both our ECG system and the commercial single-lead ECG exhibited excellent correlation in detecting ER, and cardiologists’ reviews indicated that both single-lead ECGs and 12-lead ECGs provided the same information about ER (Figure S17a,b). The second abnormality detected was a short PR interval in female volunteer 11 (Figure 6). PR intervals start at the onset of the P wave and end at the start of the QRS complex. A PR interval of less than 120 ms is considered as the short PR interval,^49^ and it may belong to normal electrocardiogram or a combination of different electrocardiographic abnormalities.^50^ It is evident that both KAUST single-lead ECG (PR interval = 110) and commercial single-lead ECG detected a short PR interval (PR interval = 98). The data from 12-lead ECG from the same volunteer also suggest the presence of a short PR interval (Figures S23a,b).

In summary, these results demonstrate that our developed single-lead ECG system is capable of detecting ECG abnormalities, making it a promising system for personal health monitoring. It is evident that printed wet ECG electrodes can be useful for short-term ECG monitoring and diagnosis using a single-lead ECG system, providing better resolution of ECG spectra and aiding in the diagnosis of ECG abnormalities. On the other hand, dry ECG electrodes require perspiration to acquire a good ECG signal,^11,12,51^ which is beneficial for users engaging in physical exercise. Dry electrodes may miss critical diagnostic information due to the low-resolution ECG signal acquired without perspiration. However, dry electrodes could be beneficial for monitoring heart rate for several days, where the R-peak can be recognized even with low-resolution ECG spectra. Therefore, it is sufficient to say that neither wet ECG electrodes nor dry electrodes are suitable for all-purpose ECG and can be beneficial for users, depending on different application scenarios.

**Figure 7:**
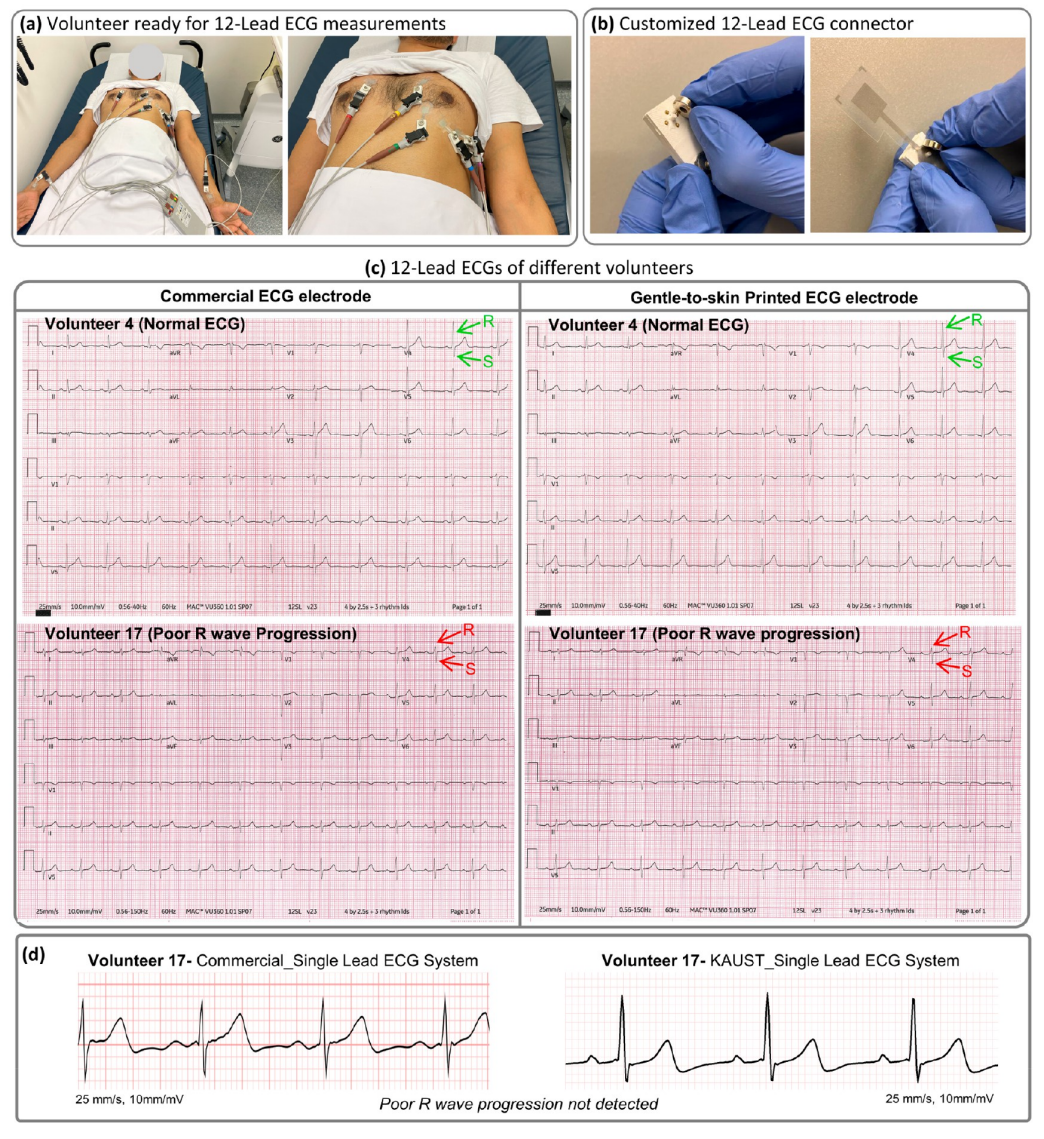
Adult volunteer 12-lead ECG study. (a) Gentle-to-skin ECG electrodes were tested on a volunteer using a 12-lead ECG system. (b) A custom-developed electrode interface utilized to connect the gentle-to-skin ECG electrodes with the 12-lead ECG system, based on the same concept of connection illustrated in Figures 1 and 3. (c) ECG data obtained from the 12-lead ECG system with gentle-to-skin ECG electrodes and commercially available ECG electrodes. The sample information was hidden from the cardiologists for a blind review. The green arrows show normal R and S wave amplitudes, and red arrows show lead V4, where the R wave is smaller than the S wave, which is evidence of poor R wave progression. (d) Single-lead ECG from volunteer 17 using the KAUST single-lead ECG system and commercial single-lead ECG system showing that poor R wave progression cannot be detected in the single lead.

Furthermore, we conducted tests on the gentle-to-skin ECG electrodes using a commercially available 12-lead ECG system (Figure 7a). We designed a special connector for the 12-lead ECG system employing the same concept of connection using pogo pins as in the case of our readout device and ECG electrodes (Figures 1b and 7b). Commercial Ag/AgCl wet ECG electrodes that are routinely available in hospitals were also used to perform an ECG test on the same volunteer (Figures S13–S32). All ECGs taken from 20 volunteers were sent to two cardiologists and one family medicine consultant for a blind review (i.e., the sample information was hidden). The complete details of the review are available in the Supporting Information Figures S13–S32. The cardiologists and family medicine consultant found that the extent of artifacts was similar in all ECGs with no differences in the voltage, intervals, axis, or PQRST morphology. The cardiologists could not differentiate between the ECGs taken with the commercial Ag/AgCl wet ECG electrodes and our gentle-to-skin fully screen-printed wet ECG electrodes. To determine abnormal ECGs, ECGs for which at least two reviewers reached a consensus and agreed on the presence of an abnormal finding were considered abnormal. For example, volunteers 3 and 11 with sinus arrhythmia (Figures S15 and S23), volunteers 5, 8, and 10 with early repolarization (Figures S17, S20, and S22), and volunteer 11 with short PR (Figure S23) were found in all single-lead and 12-lead ECGs confirming that the ECG readout and ECG electrodes are giving the similar information when compared to the commercial single-lead ECG system and electrodes. In the case of volunteer 17, 12-lead ECGs obtained using commercial and screen-printed electrodes detected poor R wave progression (Figures 7c and S29), often interpreted as indicative but not as a diagnostic and cannot rule out, possible, or probable anterior myocardial infarction (AMI).^52,53^ Poor R wave progression is usually diagnosed by looking at the lack of progressive increase in amplitude of the R wave from leads V1 to V6.^52,53^ With poor progression, in lead V4 we can see that the R wave amplitude is smaller than the S wave amplitude, which is evidence of poor R wave progression (Figure 7c). Poor R-wave progression is a common abnormal ECG finding; however, this cannot be seen using both of the single-lead ECG systems (Figure 7d) and can only be seen using the 12-lead ECG system.

We also obtained the volunteers’ feedback on the attachment and removal of the gentle-to-skin ECG electrodes compared to the commercial Ag/AgCl wet ECG electrodes. The same healthcare staff performed the attachment and removal of the commercial and gentle-to-skin electrodes, and volunteers were asked for their feedback regarding the ease of use and removal. Table 1 presents the feedback of the volunteers (scores of 1–10) as well as ECG parameters calculated from the single-lead and 12-lead ECG systems. The values indicate that the gentle-to-skin ECG electrodes scored 9.1 out of 10 in the case of ease of removal compared to the commercial ECG electrode score of 5.4. In the case of pain during the removal of ECG electrodes, gentle-to-skin electrodes scored 0.35 out of 10 (10 being the highest pain) for 20 volunteers, whereas commercial ECG electrodes scored 4.7 out of 10. Therefore, fully screen-printed wet ECG electrodes are gentle-to-skin, while providing high-quality ECG data comparable to the commercial wet ECG electrodes.

**Table 1:** Volunteer Feedback on the Ease of Removal and Pain with the Commercial and Gentle-to-Skin ECG Electrodes

Volunteer Details			Gentle-to-Skin Screen-Printed ECG Electrodes				Commercial ECG Electrodes			
Volunteer No.	Male (M) or Female (F)	Nationality	KAUST Single-Lead ECG System (Heart Rate, BPM)	12-Lead ECG (Heart Rate, BPM)	Ease of Removal (0–10)	Pain during Removal (0–10)	Commercial Single-Lead ECG System (Hear Rate, BPM)	12-Lead ECG (Heart Rate, BPM)	Ease of Removal (0–10)	Pain during Removal (0–10)
1	M	Japanese	57	63	9	0	56	59	4	5
2	M	Pakistani	61	76	9	2	60	61	2	8
3	M	Brazilian	60	61	9	0	65	60	6	4
4	M	Chinese	64	65	10	0	65	66	5	3
5	M	Indian	58	50	9	0	63	59	6	6
6	M	Pakistani	83	76	8	3	76	85	4	7
7	M	Chinese	56	61	9	0	56	60	7	5
8	M	Kazakhstani	54	57	9	0	53	55	6	4
9	M	Saudi	72	75	9	0	67	76	5	5
10	M	Indian	60	59	9	0	58	63	4	6
11	F	French	50	54	10	0	48	53	7	4
12	M	Mexican	62	65	9	0	59	68	6	4
13	F	Romanian	60	69	9	0	57	60	6	3
14	F	Saudi	61	62	10	0	61	63	5	3
15	F	Saudi	57	61	9	0	56	58	7	4
16	M	Greek	58	59	8	2	55	60	4	8
17	F	Saudi	68	69	10	0	68	72	6	3
18	F	German	59	63	9	0	60	69	5	4
19	M	Nigerian	64	64	9	0	61	66	6	4
20	F	Chinese	52	51	10	0	50	53	7	4

## Conclusion

Our smart and wearable ECG system offers gentle-to-skin fully screen-printed wet ECG electrodes and a more extended communication range, which are useful for monitoring the human heart. ECG data are wirelessly transmitted to a smartphone app and can be stored on a cloud server. The results of an adult volunteer study showed that our electrodes provide an easier-to-use option for ECG measurements and a similar ECG signal quality compared with those of commercial ECG electrodes.

The AoP encases the readout PCB and provides a longer wireless communication range in comparison to a commercial chip antenna. We tested our gentle-to-skin wet ECG electrodes on a 12-lead ECG system. We showed that they could be used in hospitals and detect abnormalities in the ECGs, such as early repolarization, short PR, and poor R wave progression, similar to commercial ECG electrodes. Our wet ECG electrodes are fabricated by a simple screen-printing method, which has the potential to produce high-quality ECG electrodes at the industrial scale that has the potential to offer affordable and gentle to the skin ECG measurements for all age groups.

## Methods

### Materials

Silver nitrate (AgNO_3_), ethylene glycol (EG), polyvinyl pyrrolidone (PVP K30 Mw = 55 000, PVP K90 Mw = 360 000), iron(III) chloride (FeCl_3_), propanediol, and ethanol were purchased from Sigma-Aldrich. PVP (K120 Mw = 3 000 000) was obtained from Ashland. A hypoallergenic Spectra 360 ECG electrode gel was purchased from Amazon. For the adhesive gel, a high-tack silicone gel A4717 kit was purchased from Factor II, Inc., USA. Artificial sweat was purchased from Nanochemazone, Canada.

### Synthesis of Silver Nanowires

AgNWs were synthesized by using a modified polyol method.^54^ Briefly, PVP (Mw = 55 000, 0.8 g, Mw = 360 000, 0.8 g) were mixed in 200 mL of EG and stirred until fully dissolved. Next, 2.1 g of AgNO_3_ (2 g) was added to the PVP solution. After complete dissolution, 30 g of FeCl_3_ seed solution (0.6 mM in EG) was added to this mixture and stirred for a few minutes. Finally, the mixture was transferred to a round-bottom flask preheated at 120 °C. After 5 h, the heating was stopped, and the reaction mixture was allowed to cool to room temperature. The AgNWs were washed multiple times with acetone and finally left in acetone.

### Preparation of Silver Nanowire Ink

AgNW ink was prepared by adding 1.5 g of PVP K120 (Mw = 2 000 000) to 27 g of propanediol and 20 g of ethanol and stirring at 600 rpm until dissolved. Finally, ∼3 wt % AgNWs was added to the solution to make the ink.

### Screen-Printing

Figure 1 shows the screen-printing of all three components of the ECG electrodes: the AgNW ink, ECG electrode gel, and adhesive gel. In general, any flexible plastic sheet can be used as a substrate provided good adhesion of AgNW ink onto the substrate. A flexible 50 μm poly(ethylene terephthalate) (PET) or 50 μm poly(ethylene naphthalate) (PEN) plastic sheet was used as the substrate. The ECG electrodes were designed in L-Edit software and exported as a DXF file. A screen mesh with a mask containing the ECG electrode design was obtained from Aurel, Italy. A screen-printing system (Aurel Screen Printer 900PA) was used to print the AgNW ink at a speed of 200 mm/s.^55^ After the first printing cycle, an air dryer was used to dry the printed AgNW. In total, three printing cycles were used. The printed AgNW structures were dried at 80 °C for 10 min and then cooled. They were then immersed in deionized water for 30 min to remove PVP and dried at 80 °C for 1 h. Next, the Spectra 360 ECG electrode gel was screen-printed by aligning the AgNWs printed on the substrate on the screen-printer. The Spectra 360 ECG electrode gel layer was then used to cover the AgNWs. A high-tack silicone gel A4717 kit was used to print the adhesive gel. Parts A and B in the kit were mixed, and air bubbles were removed using a vacuum desiccator. Then, the gel was ready for screen-printing. After alignment with the printed structures, the adhesive gel was screen-printed at a speed of 200 mm/s.

### Miniaturized Wireless Readout PCB

The miniaturized wireless readout PCB consisted of two main components: the ECG data acquisition frontend (AFE) and the BLE transceiver. MAX30003 from Maxim Integrated was used for the ECG data AFE. The AFE acquired the ECG signal from the electrodes and performed the initial filtering (bandpass filtering from 0.5 to 28.35 Hz), amplification (20 V/V), and analog-to-digital conversion (128 samples per second, 18-bit samples). The digitized ECG signal was then stored in a memory buffer in the AFE, which was accessed by the BLE system-on-a-chip (SoC) over the serial peripheral interface. We used NRF52832 from Nordic Semiconductor as the BLE SoC. The BLE SoC acquired a digitized ECG signal from the memory buffer in the AFE and made it available for the smartphone app using the BLE protocol. A Bluetooth virtual serial port (Nordic UART service) was created, over which data were transmitted to the smartphone app after a successful connection. The smartphone app displayed the ECG data to the user and sent it to a cloud server to be stored for later access or possible postprocessing by a cardiologist or artificial intelligence.^40^ The hardware of the ECG readout PCB (Figure 3) was designed on a four-layer FR4 PCB with a diameter of 1.6 cm and a thickness of 0.4 mm. Through, buried, and blind vias were used to fit all necessary routing within the available PCB area. To realize a seamless interface to the screen-printed electrodes, an innovative connection mechanism combining pogo pins and a magnetic press was designed (Figure 1b). The magnets and screen-printed ECG electrodes featured centric holes at connection sites, which mated with circular studs on the complementary part of the connector for alignment and protection against slipping from the correct position. The hole and stud sizes as well as other dimensions were optimized based on several design iterations.

### Antenna Fabrication

The AoP was completely fabricated by printing. The substrate was 3D printed from ABS, and the conductor was printed with a silver paste. Thus, the fabrication followed the concept of additive manufacturing to reduce material waste. A 3D model of the substrate was designed in HFSS. The 3D printer Raise3D Pro2 was used to fabricate the designed substrate. A thickness of 0.5 mm gave the optimal balance between the mechanical strength and dielectric loss. Another thin cap was 3D-printed and installed under the ground plane to protect the electronic chips on the exposed PCB during integration with the wireless ECG readout PCB. This cap also helped maintain the proper distance between the AoP and human chest for stable performance. Four snap fits were used to integrate the thin cap with the rest of the AoP and make it easy to remove and install. The cap had a thickness of only 0.5 mm, so it had a very slight effect on the AoP radiation. The conductor was manually printed with a mask. The 2D pattern of the conductor was initially designed in HFSS and was transferred to CGSTAPE-8358 heat-resistant tape by a VLS 3.50 Laser. Parameters such as the height, power, and speed were optimized to obtain an accurate pattern width. Then, we wrapped the tape around the AoP and took off the conductor segments. The silver paste was printed afterward. Then, the semifinished sample was placed in an oven for 3 h at 70 °C to cure the silver paste. The optimal temperature was determined as 70 °C, which resulted in good conductivity. After the silver paste was cured, a conductive epoxy was used for mechanical fixing and electrical connections. A U.FL connector was connected to the RF signal input for the measurements. A chip capacitor was inserted in the sideline for impedance matching. For testing of the antenna performance, the whole system was placed on the volunteer’s chest, the smartphone performs as a receiver in the forward direction, and the received power was recorded at different distances in the open area. The output power of the BLE chip was set to 0 dBm, and the received power was compared to that of the commercial chip antenna. The received power was approximately 11 dB larger than that of the chip antenna. Since the sensitivity of the smartphone is −96 dBm, the communication range of the proposed antenna was estimated to be 142 m, which is 4× longer than that of the chip antenna.

### Smartphone App and Cloud Server Software

The smartphone app was designed in a native Android development environment and provided the functionality to acquire data from the custom ECG monitoring device. The app starts with user registration. Each user is only registered once they provide informed consent to comply with the ethical requirements of our university’s bioethics committee (IBEC approval no. 22IBEC011). The user registration, profile, and data are stored on a cloud server, so the user can log on to their account from any other smartphone or Internet-enabled device. Once logged in, the user can view the previous history or record the ECG data. If the user opts to view previous history, a list of previous ECG scans stored against the user’s profile in the cloud server database is presented. The user can then click on any entry to view the corresponding ECG waveform. If the user opts to record another ECG, then the app performs a BLE scan and presents the user with a list of ECG devices in the vicinity. Our ECG device is broadcast as a connectable peripheral with a particular naming format (i.e., BED_[XXXX], where XXXX is an exclusive alphanumeric identifier). The user can then click on an ECG device to select it. The smartphone app then sends a connection request to the selected device and, upon successful connection, starts receiving and displaying ECG data from the device on a Bluetooth virtual serial port (Nordic UART service). At the end of data collection, the user can save all acquired ECG data on the cloud server. The cloud server was developed on a Google Firebase, and it provides basic authentication, user registration, and ECG data storage against user profiles.

### ECG Measurements

The adult volunteer study was conducted with the approval of our institute’s bioethics committee (IBEC approval no. 22IBEC011). All volunteers were first fully briefed about the experiments and given a consent form to read and agree to before the ECG tests. The ECG measurements were conducted by using fully screen-printed wet ECG electrodes and commercially available ECG electrodes with a wearable ECG system and a 12-lead ECG system (GE Healthcare MAC VU 360). ECG measurements for optimization of the electrodes were taken by using the commercially available evaluation kits for MAX30003 and NRF52832. For adult volunteer studies, in the rest–exercise–rest test and extended measurement interval test, two data collection setups were used. The first data collection setup was the wearable ECG system presented in this paper and fully printed wet ECG electrodes. Data were viewed on custom designed smartphone app (ECG waveform) and also collected on “Bluefruit Connect” Android app (for numerical data export), developed and made publicly available by Adafruit industries. The second data collection setup was a commercial single-lead ECG system (Getwellue ECG system) with commercial electrodes (3M Red dot ECG electrodes). For the exercise test, a silver paste was placed at the point of connection between the pogo pins and printed silver nanowires to avoid disconnection during the exercise test. For extended study with diverse ethnic backgrounds, genders, and age groups, four data collection setups were used. The first data collection setup was a commercial 12-lead ECG system (GE Healthcare MAC VU 360) with commercial ECG electrodes (3M Red dot ECG electrodes). The second data collection setup was the commercial 12-lead ECG system (GE Healthcare MAC VU 360) with fully printed ECG electrodes. The third data collection setup was the commercial single-lead ECG system (Getwellue ECG recorder) with commercial electrodes (3M Red dot ECG electrodes). Finally, the last data collection setup was the wearable ECG system presented in this paper and the fully printed ECG electrodes. Data were viewed on custom designed smartphone app (ECG waveform) and also collected on the “Bluefruit Connect” Android app (for numerical data export), developed and made publicly available by Adafruit industries. All of the data collected were plotted using Origin Pro software. MATLAB scripts were employed for calculating PQRST peaks and dips as well as for determining the R–R interval to calculate the heart rate. In the case of commercial ECG systems, the heart rate was calculated and reported using the built-in proprietary software in the commercial 12-lead ECG system (GE Healthcare MAC VU 360) and the commercial single-lead ECG system (Getwellue single-lead ECG recorder).

## Supporting Information Available

Thixotropic properties of silver nanowires (AgNWs), ECG electrode gel and adhesive gel inks, cost analysis of ECG electrodes, ECG electrode response optimization, peel test for ECG electrodes, operational stability of ECG electrodes, sweat tolerance studies of ECG electrodes, schematic of ECG PCB, single-lead and 12-lead ECG data of volunteers (PDF)
Video 1: Screen-printed electrode adhesion test (MP4)
Video 2: Screen-printed electrode removal test (MP4)
Video 3: Commercial electrode removal test (MOV)
Video 4: Cyclic bending test (MP4)
Video 5: Demonstration of werable ECG system (MOV)

## Supplementary Material

nn3c12477_si_003.mp4

nn3c12477_si_004.mov

nn3c12477_si_005.mp4

nn3c12477_si_006.mov
