# Supplementary material for: Fully Screen-Printed and Gentle-to-Skin Wet ECG Electrodes with Compact Wireless Readout for Cardiac Diagnosis and Remote Monitoring
Source: ACS Nano. 2024 Mar 25;18(14):10074–87. doi: 10.1021/acsnano.3c12477 (PMC11022287; doi:10.1021/acsnano.3c12477)
Supplement: Supplementary file 1 — nn3c12477_si_001.pdf [file nn3c12477_si_001.pdf]

Supporting Information

Fully Screen-Printed and Gentle-to-Skin Wet ECG Electrodes with Compact Wireless Readout for Cardiac Diagnosis and Remote Monitoring

Sakandar Rauf,*[1] Rana M. Bilal,[1] Jiajun Li,[1] Mohammad Vaseem,[1] Adeel N. Ahmad,[2] and Atif Shamim[1]*

[1]Electrical and Computer Engineering, CEMSE, King Abdullah University of Science and Technology (KAUST), Thuwal, 23955-6900, Kingdom of Saudi Arabia.

[2]School of Medicine, University of Nottingham, Nottingham, NG7 2UH, United Kingdom.

E-mail: sakandar.rauf@kaust.edu.sa; atif.shamim@kaust.edu.sa

Thixotropic Properties of Silver Nanowires (AgNWs), ECG Electrode Gel and Adhesive Gel Inks

To confirm the thixotropic behavior of AgNWs ink, ECG electrode gel and adhesive gel, the viscosity of these solutions were measured using a rheometer at a shear rate from 0.01 to 1000 s^{-1}, and the corresponding curves are displayed in Figure S1 (a-c). We observed a shear-thinning behavior of the AgNW ink as well as electrode gel and adhesive gel. For example, the ink viscosity decreased from 511 to 0.33 Pa.s as the shear rate increased from 0.01 to 10 s^{-1} for Ag NWs ink. Similarly, a decrease in viscosity from 3301 to 27 Pa.s and from 483 to 57 Pa.s has been observed for ECG electrode gel and adhesive gel, respectively as the shear rate increased from 0.01 to 10 s^{-1}. This type of fluid behavior and viscosity is essential for good printability. However, it should be noted that the viscosity decrease is slower in the case of adhesive gel compared to the ECG electrode gel and AgNWs ink in the shear rate range from 1 to 250 s^{-1} possibility due to the polymerization reaction of two components of the silicon adhesive gel A4717. Next, to confirm the loading of the

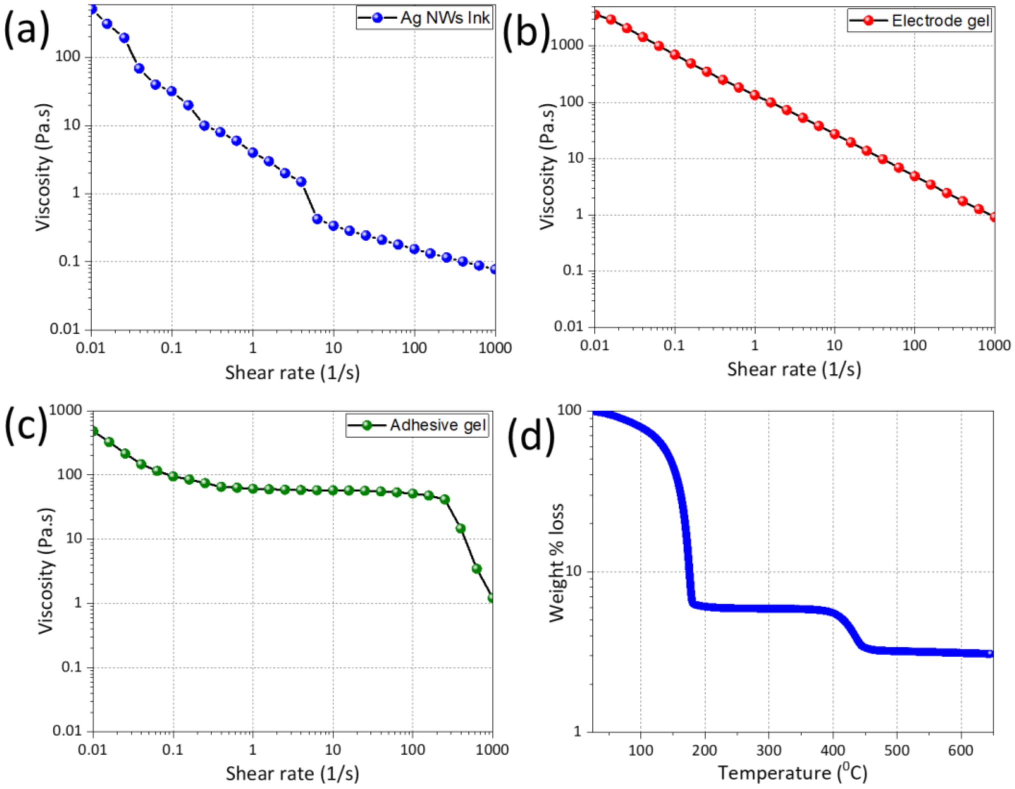

Figure S1: The measured viscosity of (a) Ag NWs ink (b) electrode gel and (c) adhesive gel. Thermogravimetric analysis (TGA) analysis of (d) Ag NWs ink.

AgNWs, the as-formulated ink was further characterized using thermogravimetric analysis (TGA), as shown in Figure S1(d). The TGA results confirmed a sharp decrease in the weight % loss between 70 and 200 °C; this corresponds to the temperature range at which the ink solvents (propanediol and ethanol) evaporated. The

weight % loss between 400 and 450 °C was mainly due to the thermal decomposition of the PVP K-120 polymer. The weight % beyond 600 °C corresponded to the ink's solid loading of the AgNWs. The added weight % of the AgNWs in ink is well matched with that of the corresponding thermally decomposed Ag. After the ink characterizations, the ink was used to print ECG electrodes using a screen-printing system (AUREL screen-printer 900PA).

Cost Analysis of ECG Electrodes

**Component**	**Cost Determination**
ECG Electrode	**Synthesis of silver nanowire ink**<br>0.8 g PVP (K90) = 0.2192 € (274 €/Kg, Sigma-Aldrich)<br>0.8 g PVP (K30)= 0.368 € (230€/Kg, Sigma-Aldrich)<br>230 mL Ethylene Glycol = 11.19 € (48.67€/L, Sigma-Aldrich)<br>2.1g Silver nitrate = 10.353 € (493€/100g, ACS reagent, Sigma-Aldrich)<br>0.0162 g Iron chloride= 0.0012 € (74.10/Kg, Sigma-Aldrich)<br>Washing with 1200 ml Acetone = 1.84 € (7.7 €/5L, Sigma-Aldrich)<br>10 ml Ink vehicle = 1.5 €<br>*The estimated cost of 10 ml Silver nanowire ink = 25.6 € or 27.46 USD*<br>*10ml ink produces approx= 80 ECG electrodes*<br>*Cost of ink per electrode= 0.343 USD per electrode*<br><br>*Cost of PET substrate per electrode*<br>500 Kg roll of PET (0.1mm)= 755 USD (Alibab.com)<br>*0.1792 g PET used for one ECG electrode = 0.000271 USD per electrode*<br><br>*Cost of ECG electrode gel per electrode*<br>Spectra 360 ECG electrode gel = 9.28 USD/250g or 0.03712 USD/g<br>2.57 mg of 360 ECG electrode gel per electrode = 0.0000954 USD per electrode<br><br>High Tack Silicon Gel 1:1, 2-pound (0.9072 Kg) A4717 = 87.95 USD<br>Weight of Hightack gel per electrode= 0.0114 g<br>High Tack Silicon Gel 1:1, 2-pound (0.9072 Kg) A4717 per electrode = 0.00111 USD<br><br>Screen printing cost per electrode= 0.01 USD<br><br>**Estimated lab scale cost per ECG electrode = 0.35 USD per electrode**<br><br>**Estimation of industrial scal cost of most expensive reagents**<br>*Industrial cost of only two expensive reagents from Alibaba.com*<br>Ethylene glycol used 0.25 g (690USD/Ton or 907 Kg)= 0.19 USD (Alibaba.com)<br>Price silver nitrate AgNO3 powder 99.8%, Reagent Grade (450 USD/Kg)= 0.945 USD (Alibaba.com)<br>*Based on these, estimating 10 times less cost for all the reagents*<br>0.8 g PVP (K90) = 0.02192 € (274 €/Kg, Sigma-Aldrich)<br>0.8 g PVP (K30)= 0.0368 € (230€/Kg, Sigma-Aldrich)<br>0.0162 g Iron chloride= 0.00012 € (74.10/Kg, Sigma-Aldrich)<br>Washing with 1200 ml Acetone = 0.184 € (7.7 €/5L, Sigma-Aldrich)<br>10 ml Ink vehicle = 0.15 €<br>*The estimated cost of 10 ml Silver nanowire ink = 1.527 € or 1.63 USD*<br>*10ml ink produces approx= 80 ECG electrodes*<br>*Cost of ink per electrode= 0.020 USD per electrode*<br><br>*Cost of PET substrate per electrode*

	500 Kg roll of PET (0.1mm)= 755 USD (Alibab.com)<br>*0.1792 g PET used for one ECG electrode = 0.000271 USD per electrode*<br><br>*Cost of ECG electrode gel per electrode*<br>Spectra 360 ECG electrode gel = 9.28 USD/250g or 0.03712 USD/g<br>2.57 mg of 360 ECG electrode gel per electrode = 0.0000954 USD per electrode<br><br>High Tack Silicon Gel 1:1, 2-pound (0.9072 Kg) A4717 = 87.95 USD<br>Weight of Hightack gel per electrode= 0.0114 g<br>High Tack Silicon Gel 1:1, 2-pound (0.9072 Kg) A4717 per electrode = 0.00111 USD<br><br>Screen printing cost per electrode= 0.01 USD<br><br>**Estimated industrial scale cost per ECG electrode = 0.03 USD per electrode**
ECG PCB, antenna, and connection interface	ECG PCB plus battery for 1000 units<br>Components cost/set= 15.91 USD<br>Assembly cost/set = 1 USD<br>Packing cost/set = 0.1 USD<br>PCB cost/set = 0.5 USD<br>Battery = 0.3 USD<br>***PCBA set total = 17.81 USD***<br>PCB tooling = 125 USD<br>Assembly engineer cost = 46 USD<br>Total cost = 17810+125+46 = ***17681 USD for 1000 pieces***<br>***Cost per PCB piece= 17.981***<br>***3D Printed Antenna and connection interface = 2 USD per piece***
**Total cost of ECG readout**	**19.98 USD ≅ 20 USD**

Table S1: Estimated cost of the ECG electrode and ECG readout

**ECG Electrode Fabrication Technique**	**ECG Electrode Material**	**ECG Electrode Mass Manufacturing Capability**	**Direct Pattern Capability**	**ECG Electrode Fabrication Speed**	**Competitive for Low cost Manufacturing**	**Reference**
Spin Coating	Silver nanowires & PDMS[1]	No	No	Slow	No	**1**
Spray Coating	Electroconductive polymer spray-coated 3D porous graphene[2]	Possible	No	Slow	No	**2**
Electrospinning	Nanofiber-Reinforced Silver Nanowires[3]	No	No	Slow	No	**3**
Vacuum Filtration Method	Silver Nanowire/Polydimethylsiloxane[4]	No	No	Slow	No	**4**
Diect write 3D printing	Conductive silver paste,[5]	Possible	Yes	Medium	Yes	**5**
3D printing and Molding	Liquid Metal Composite[6]	Possible	Yes	Medium	Yes	**6**
Inkjet Printing	PEDOT:PSS Electrodes[7]	Possible	Yes	Fast	Yes	**7**
Electroplating Textile	Textile based on Ag/AgCI electro-plating materials[8]	Possible	No	Slow	No	**8**
Screen Printing	Silver nanowires with graphene oxide (GO)[9], silver nanoparticles and copper nanowires[10], Ag/AgCl Electrode on Textiles[11] PEDOT:PSS Electrodes[12], Graphene electrode on textile[13] *(In all cases only the conductive part was screen printed)*	Possible	Yes	Fast	Yes	**9, 10, 11**<br>**12**<br>**13**
Screen printing and coating	Printing of silver paste and coating of silicone adhesive[14]	Possible	Yes	Medium	Yes	**14**
Screen Printing (This Study)	***Silver Nanowire ink, ECG electrode gel and Adhesive gel (All components screen printed)***	***Possible***	***Yes***	***Fast***	***Yes***	***This Study***

Table S2: Advantages and disadvantage of different techniques reported in the literature for the fabrication of ECG electrodes compared to the fully screen printing method.

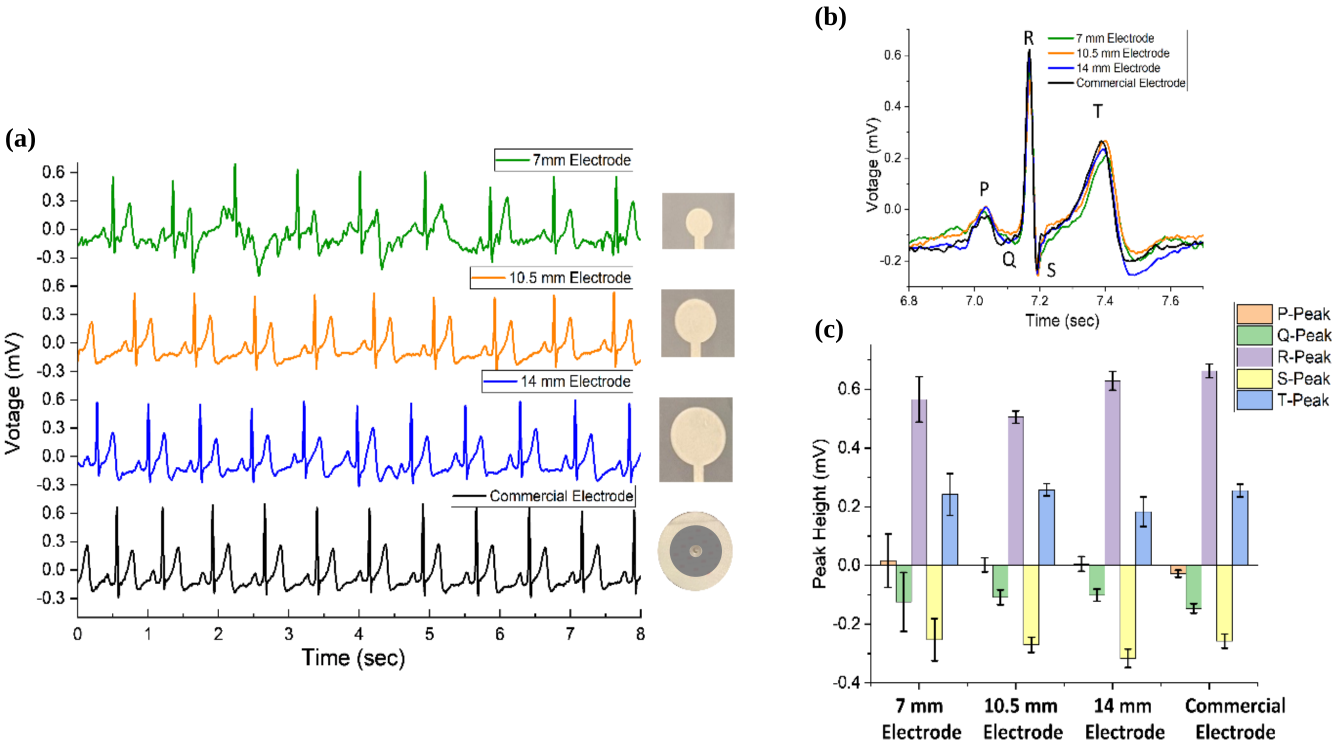

Figure S2: a. ECG signal from an adult volunteer using a pair of different sizes of screen-printed circular electrodes (7 mm, 10.5 mm and 14 mm) and commercial ECG electrodes. The ECG electrodes were placed at the volunteer's chest at 8 cm center-to-center distance. **b,** The graph shows a normal sinus rhythm ECG wave with characteristic ECG peaks (P, Q, R, S, and T) for each electrode type. **c,** Characteristic peak amplitudes of ECG signal (P, Q, R, S, and T) plotted to compare data acquired from different sizes of screen-printed circular electrodes and commercial electrodes. The error bars show the standard deviation of peak amplitudes calculated for a 8s ECG for each electrode type.

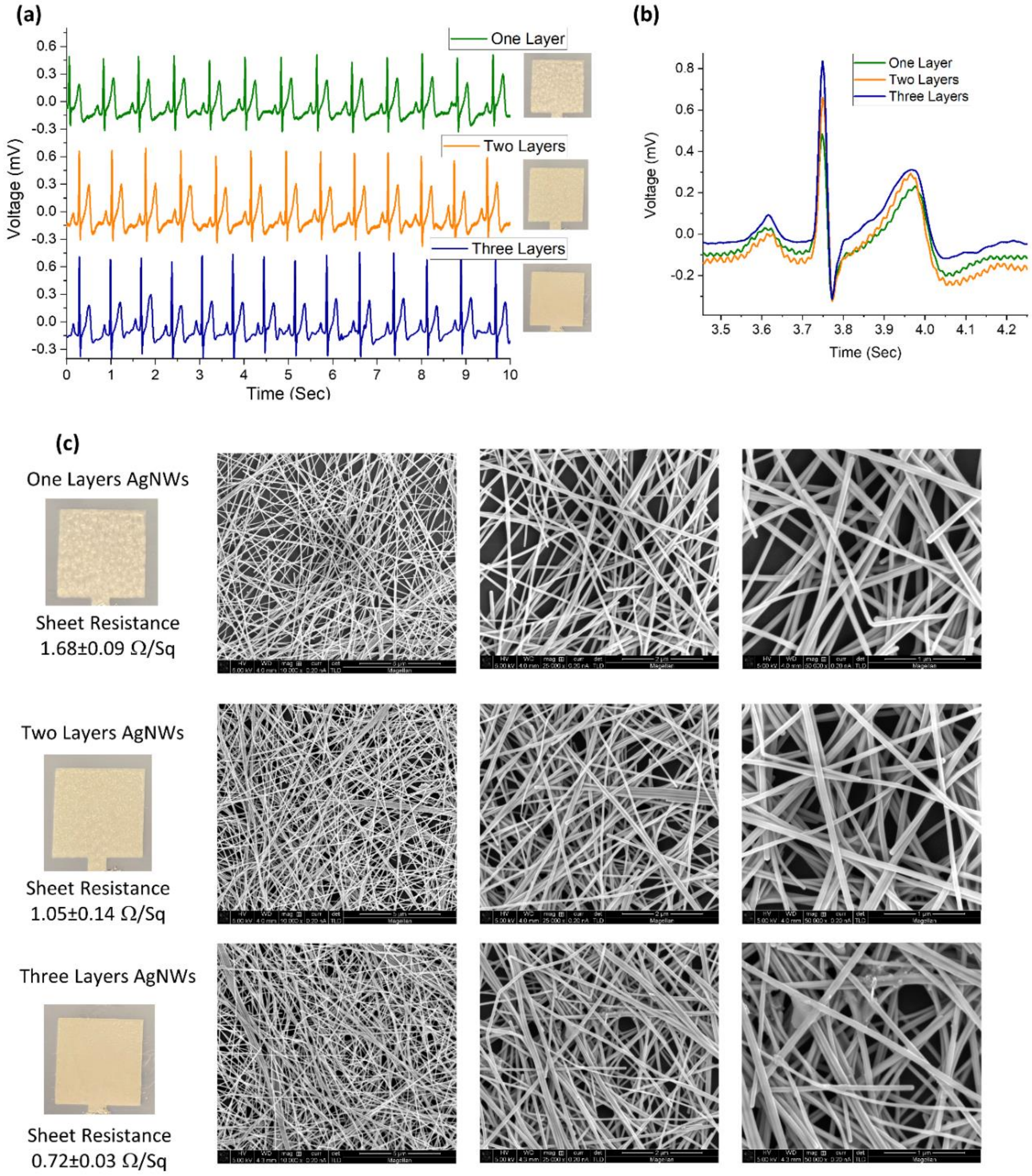

Figure S3: a. ECG signal from an adult volunteer using a pair of screen-printed square electrodes with one layer silver nanowires, two layer silver nanowires and three layers silver nanowires. The ECG electrodes were placed at the volunteer's chest at 8 cm center-to-center distance. **b,** The graph shows a normal sinus rhythm ECG wave with characteristic ECG peaks (P, Q, R, S, and T) for each electrode type. **c,** Pictures of the ECG electrodes and subsequent sheet resistance obtained after printing one layer, two layers and three layers of silver nanowires (left). Scanning electron micoscope images of the electrodes obtained after one layer, two layers and three layers of silver nanowires

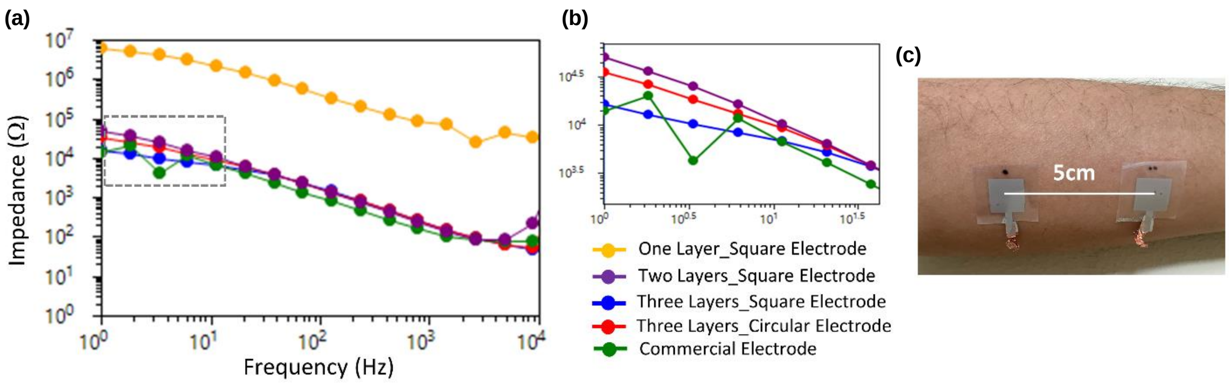

Figure S4: a, Impedance measurements of electrode-skin contact as a function of the frequency for different layers of silver nanowires screen-printed square electrodes, circular screen-printed electrodes, and commercial ECG electrodes. **b,** magnified graph segment shows the difference between the impedance of screen-printed square, screen-printed circular, and commercial ECG electrodes. **c,** a picture showing the position of the electrodes placed on a volunteer's arm. The electrodes center to center distance was 5cm. The impedance measurements were conducted using a potentiostat (Sensit Smart, Palmsens).

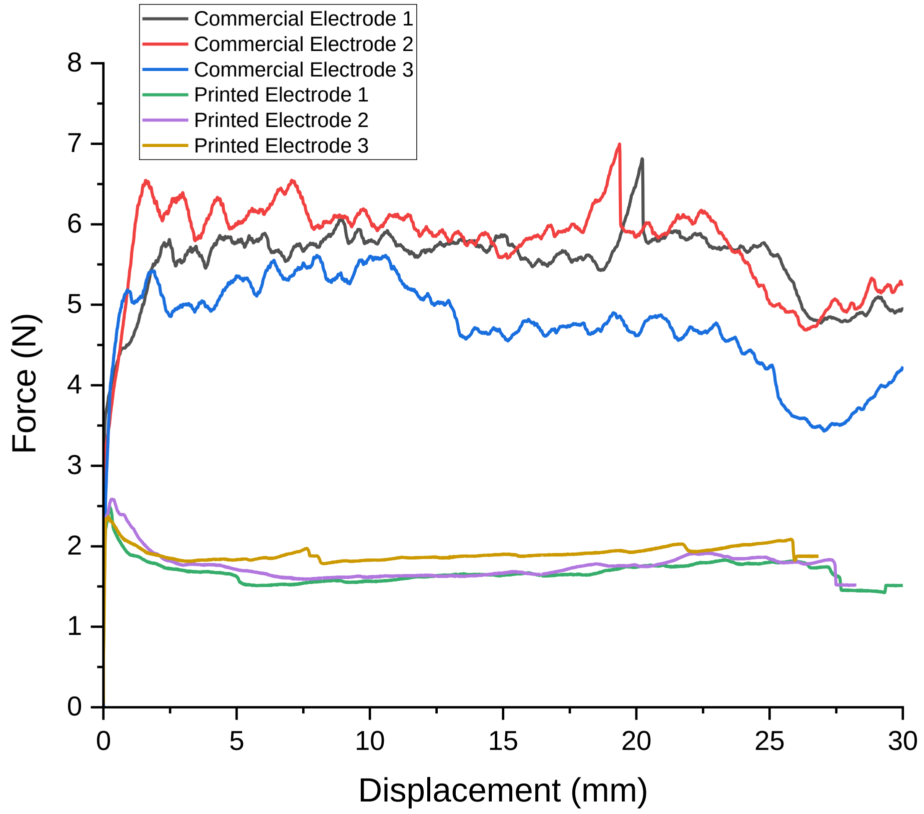

Figure S5: Peel test results of the commercial ECG electrode and screen-printed ECG electrode. The peeling angle was 90°. The peeling test was conducted using a universal testing machine (ZwickRoell Z0.5 TN) with a strain rate of 20 mm/min and using a load cell of 200 N.

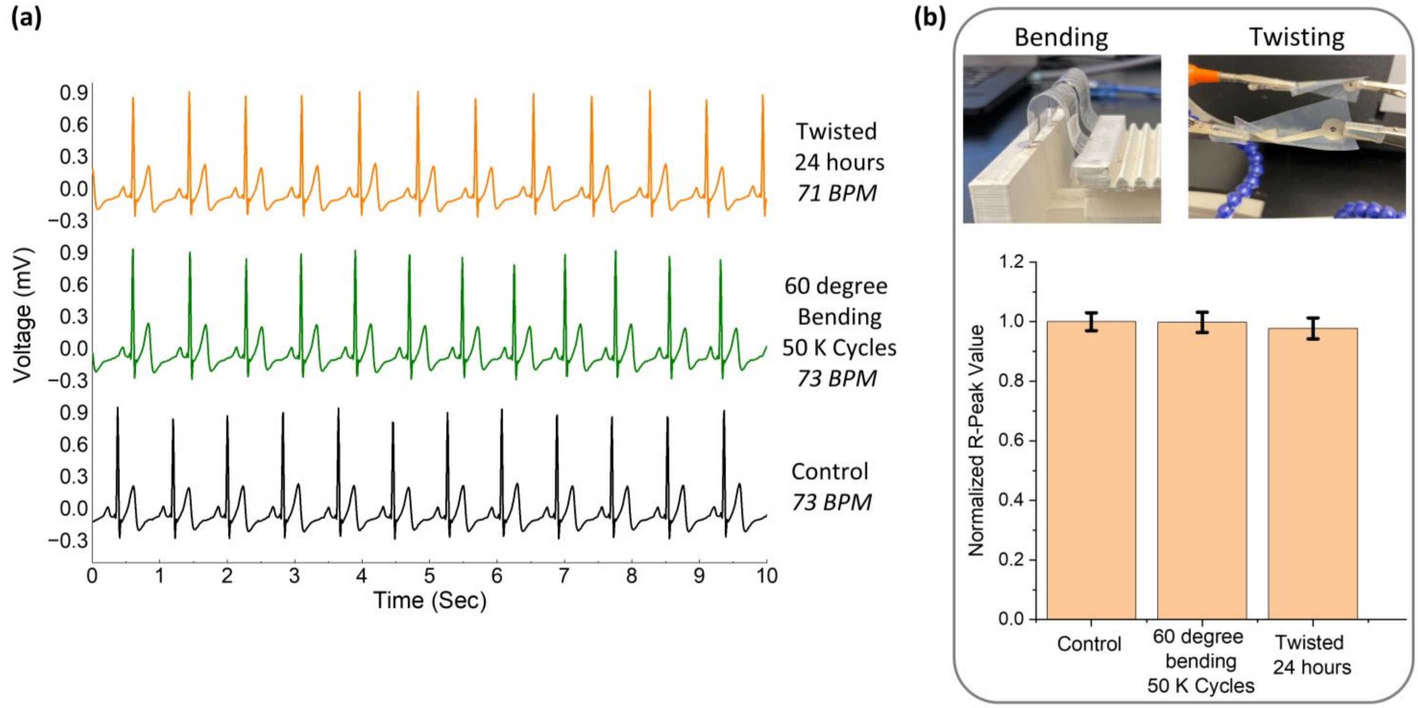

Figure S6: (a) Displays ECG data after bending cycles and twisting of printed ECG electrodes. In each case, ECG data were acquired for one minute, with only 10 seconds shown for clarity of the ECG spectra. The ECG electrode gel was not initially printed on the electrode to prevent its dryout during the test; instead, it was printed on top of the conductive part afterward. The figure also includes the average heart rate in BPM for each case. (b) Illustrates the effect of bending cycles and twisting of printed ECG electrodes on the ECG signal by plotting the normalized R-peak value in each case.

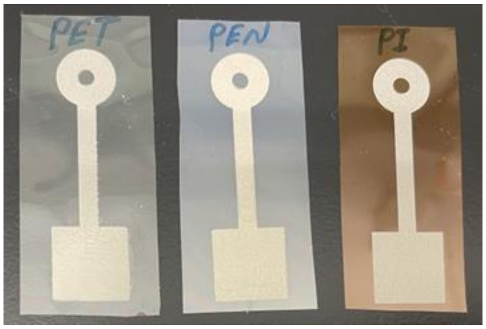

Figure S7: Screen printing of silver nanowire ink on different plastic substrates polyethylene terephthalate (PET), polyethylene naphthalate (PEN) and Polyimide (PI). ECG electrodes can be fabricated using all three plastic substrates.

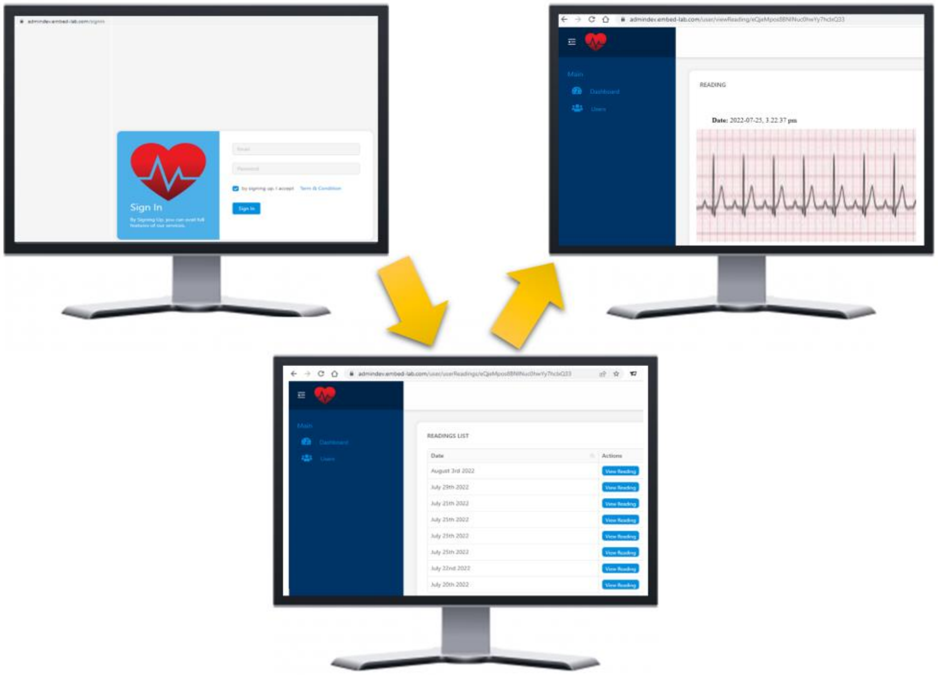

Figure S8: Proof-of-concept cloud server components.

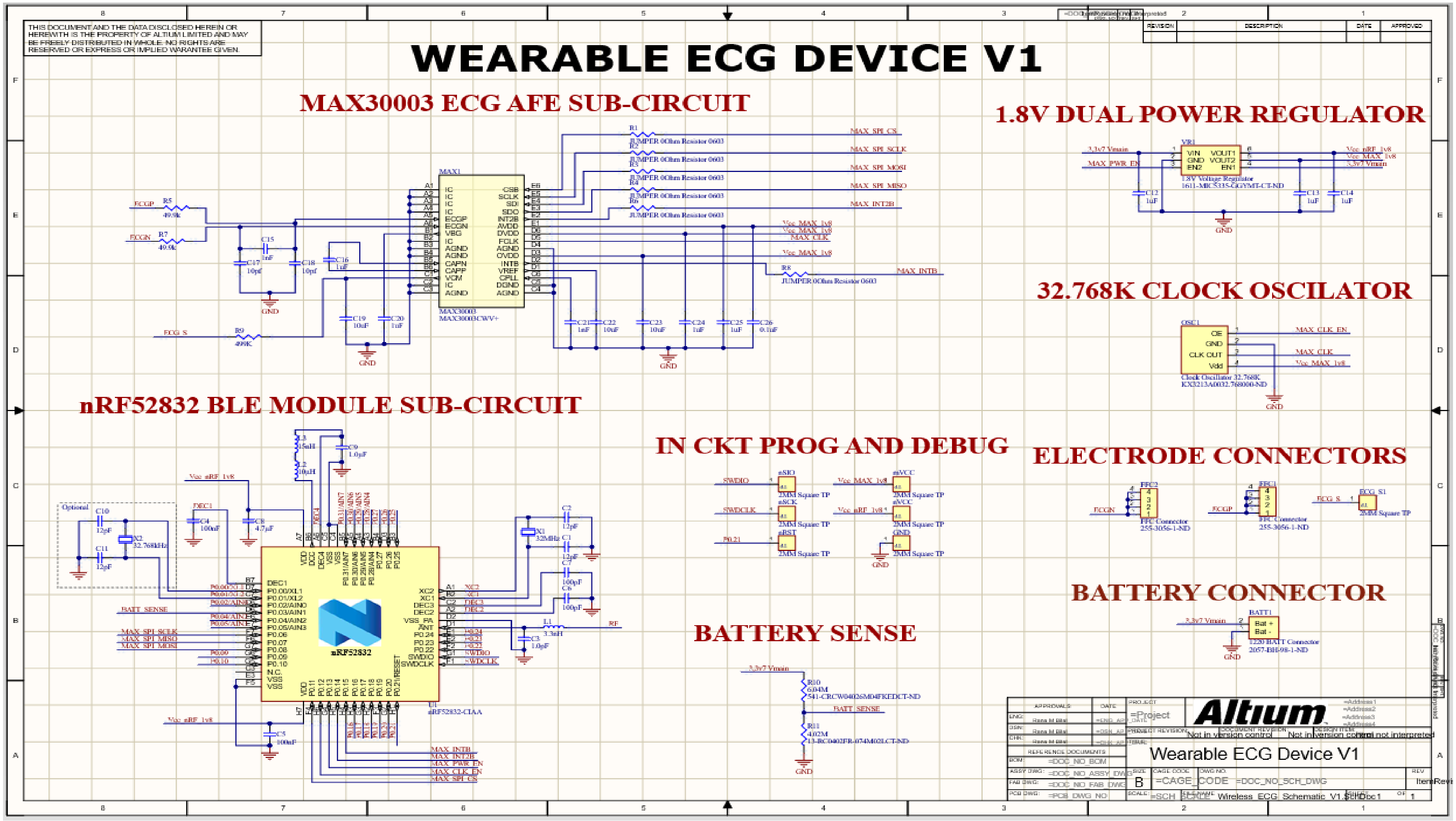

Figure S9: Schematic of the readout device for the wearable 2 electrode, single lead miniaturized wireless ECG system. Readout device consists of an Analog ECG front end formed by Maxim's MAX30003 system on chip, Bluetooth low energy wireless transmission module formed by Nordic semiconductor's nRF52832 system on chip, power regulation modules, clock oscillators and other support circuit elements such as battery sensing resistors, in circuit programming connectors and connectors for fully printed ECG electrodes.

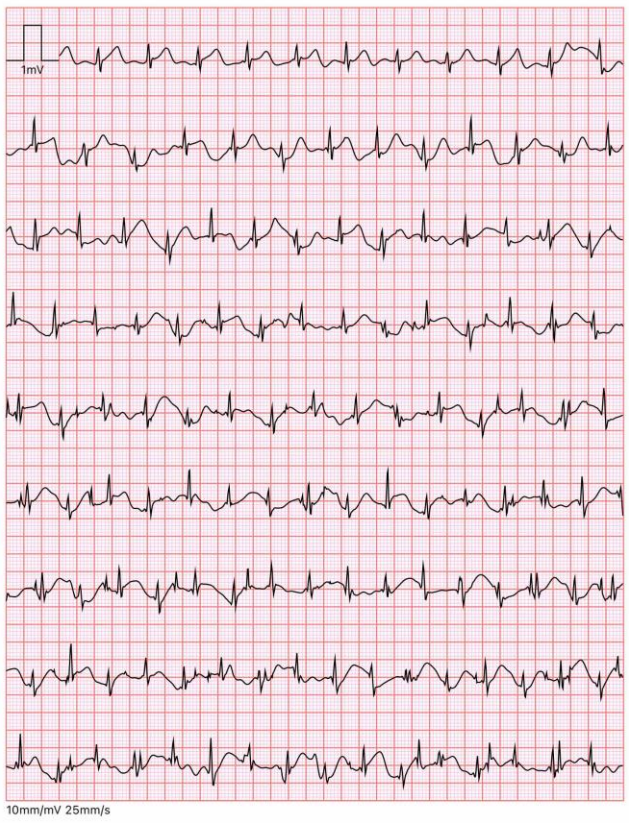

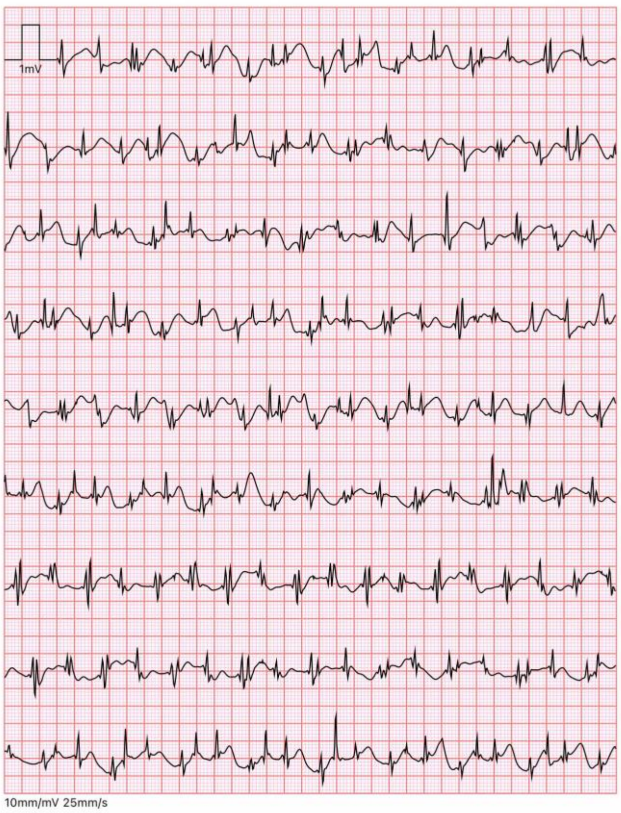

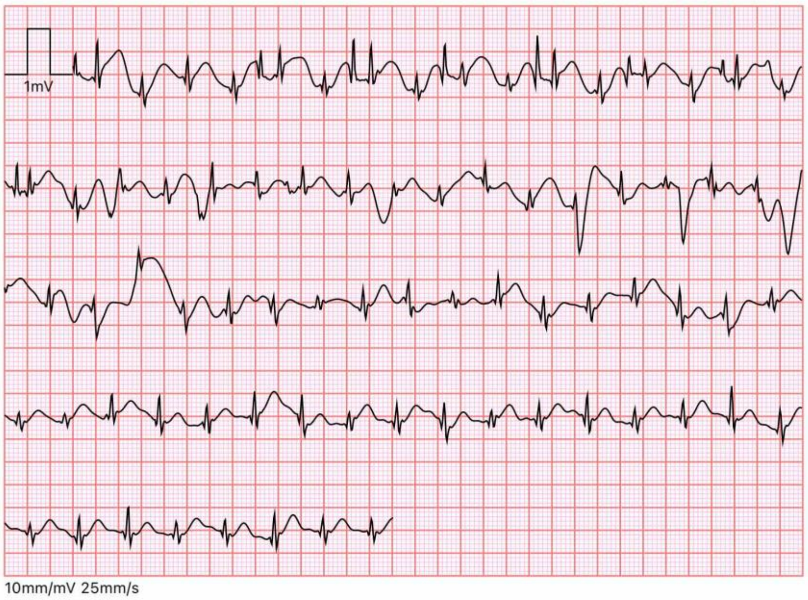

Figure S10: ECG data obtained during exercise using a commercially available wearable ECG monitor and commercial wet ECG electrodes. The baseline drift can be seen as the volunteer started the exercise.

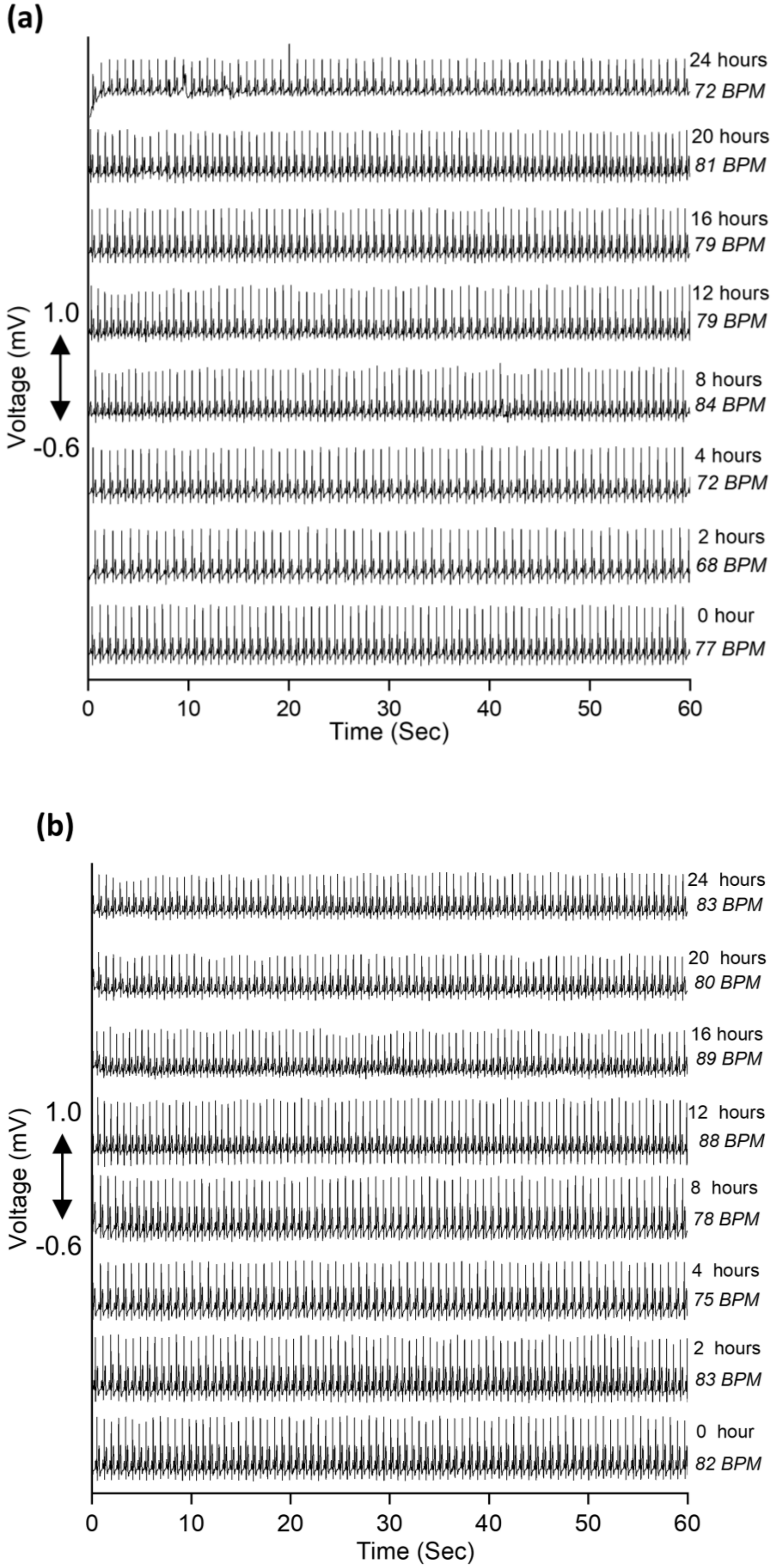

Figure S11: Comparison of the long-term wearability of (a) printed wet ECG electrodes and (b) commercial wet ECG electrodes.

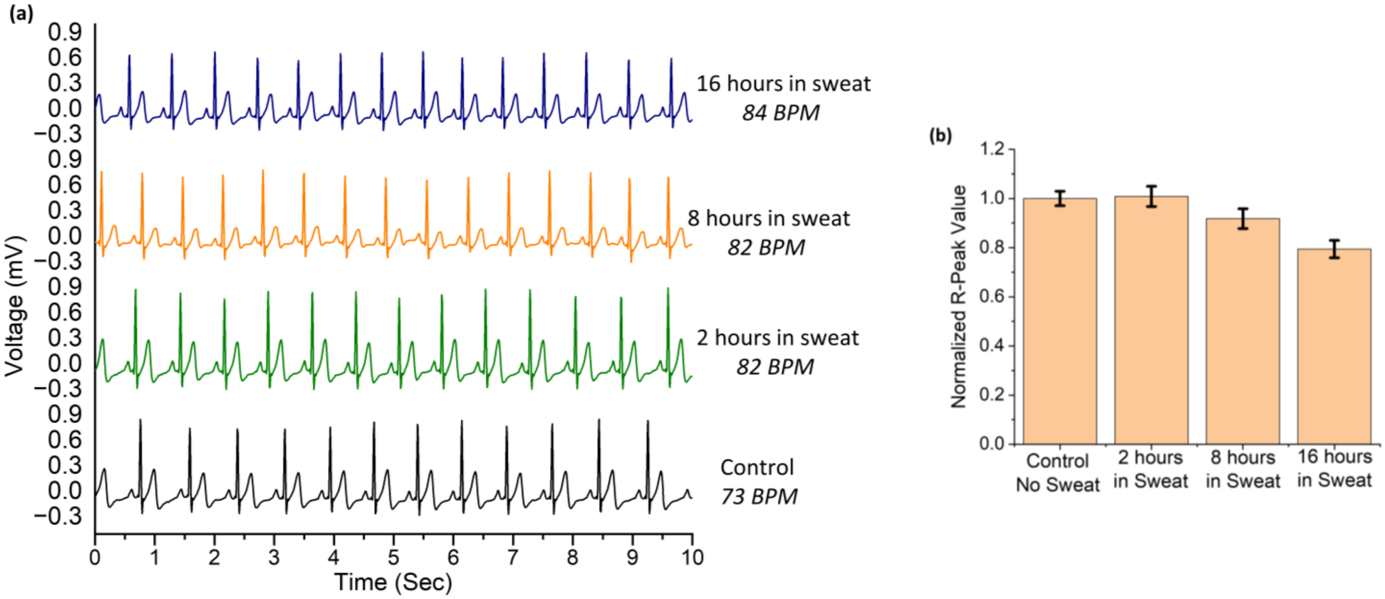

Figure S12: Sweat tolerance of the printed wet ECG electrodes: (a) ECG acquired after the addition of artificial sweat onto the ECG electrodes for different time intervals (2, 8, and 16 hours). In each case, ECG data were acquired for one minute. Only 10 seconds of data are shown for clarity of the ECG spectra. The figure also displays the average heart rate in BPM for each case. (b) The effect of sweat on the ECG signal is illustrated by plotting the normalized R-peak value in each case.

Cardiology and Family Medicine Consultants Review

Volunteer No	ECG Type	Leads	Reviewer 1 (Cardiology Consultant)	Reviewer 2 (Cardiology Consultant)	Reviewer 3 (FM Consultant)
V1	A	12	Normal Sinus rhythm, Normal PR interval, normal ST segements	Normal sinus rhythm, normal intervals, normal ST segments	Normal sinus rhythm, normal intervals, normal ST segments
	B	12	Normal Sinus rhythm, Normal PR interval, normal ST segements	Normal sinus rhythm, normal intervals, normal ST segments	Normal sinus rhythm, normal intervals, normal ST segments
	KAUST	1	Normal Sinus rhythm, Normal PR interval, normal ST segements	Normal sinus rhythm, normal intervals, normal ST segments	Normal sinus rhythm, normal intervals, normal ST segments
	Commercial	1	Normal Sinus rhythm, Normal PR interval, normal ST segements	Normal sinus rhythm, normal intervals, normal ST segments	Normal sinus rhythm, normal intervals, normal ST segments
	Summary		All 4 ECGS had similar interpretation	All 4 ECGS had similar interpretation	All 4 ECGS had similar interpretation

Volunteer 1_KAUST_Single lead ECG system

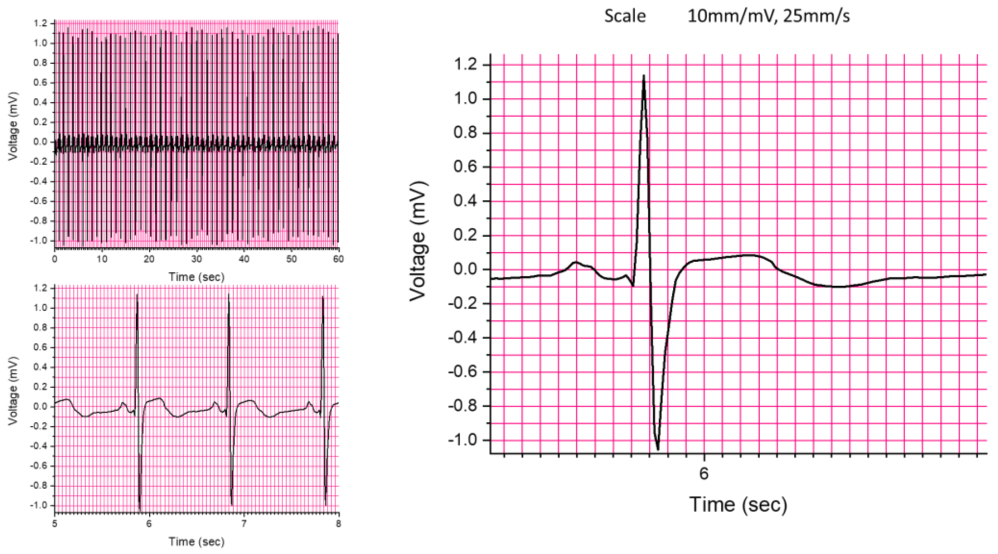

Volunteer 1_Commercial_Single lead ECG system

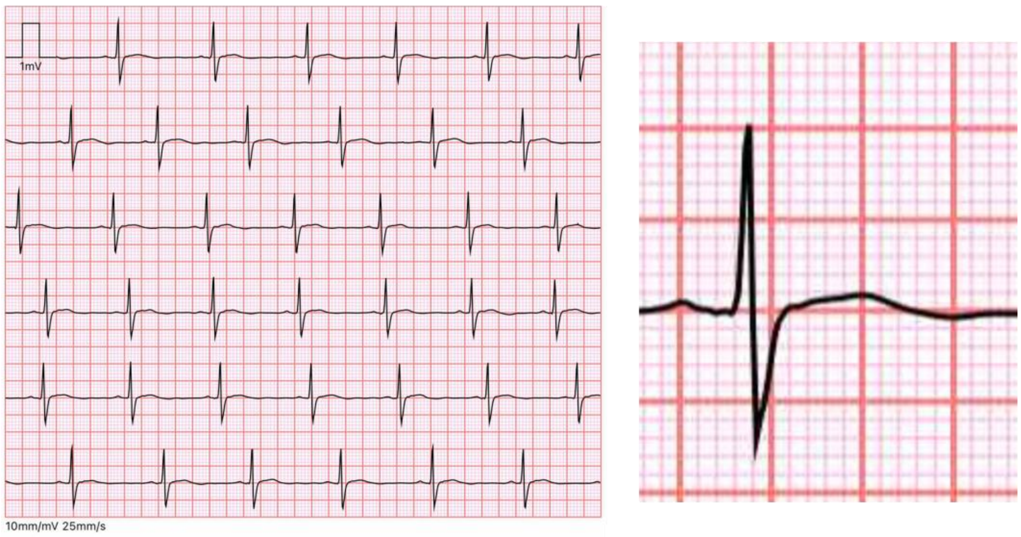

Figure S13a: Volunteer 1, KAUST single lead, and commercial single-lead ECG.

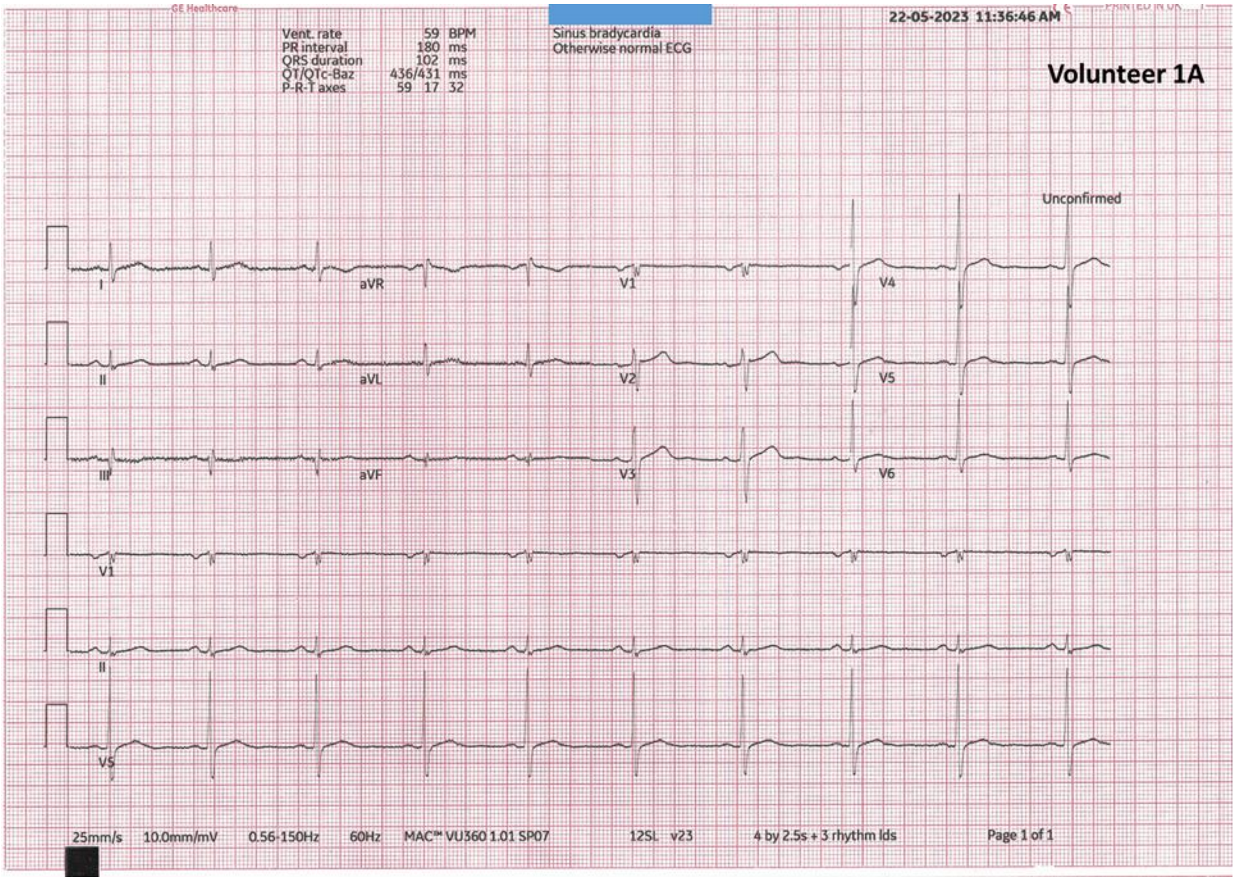

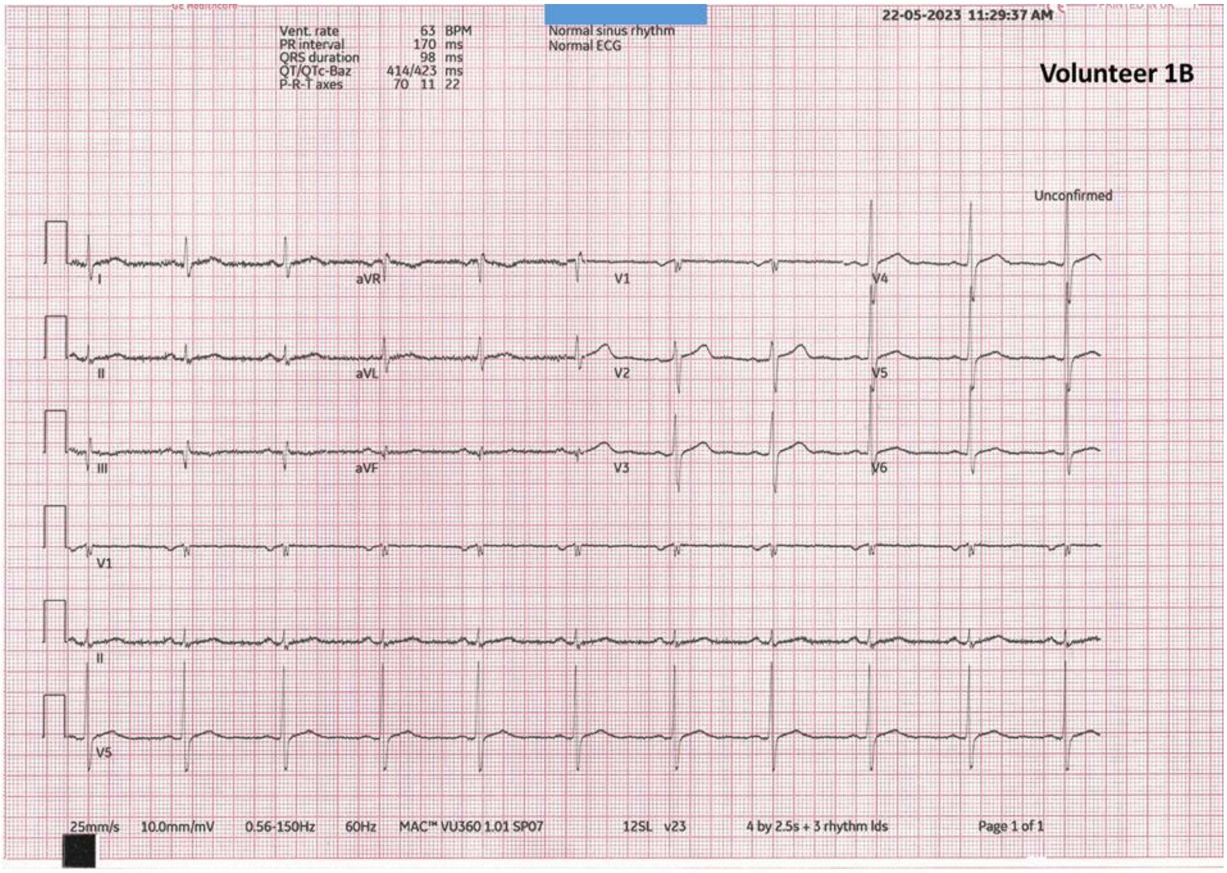

Figure S13b: Volunteer 1: Blinded review of 12 lead ECG using commercial ECG electrodes and gentle-to-skin screen printed ECG electrodes. The sample identification was hidden from the reviewers using a blue color square.

Volunteer No	ECG Type	Leads	Reviewer 1 (Cardiology Consultant)	Reviewer 2 (Cardiology Consultant)	Reviewer 3 (FM Consultant)
V2	A	12	Normal Sinus rhythm, Normal PR interval, normal ST segements	Normal SR, ST elevation consistent with early repolarization	Normal sinus rhythm, normal intervals, normal ST segments
	B	12	Normal Sinus rhythm, Normal PR interval, normal ST segements	Normal SR, ST elevation consistent with early repolarization	Normal sinus rhythm, normal intervals, normal ST segments
	KAUST	1	Normal Sinus rhythm, Normal PR interval, normal ST segements	Normal SR, Non-specefic ST-T changes. Inverted T wave	Normal sinus rhythm, normal intervals, normal ST segments
	Commercial	1	Normal sinus rhythm, normal intervals	Normal SR, Non-specefic ST-T changes. Inverted T wave	Normal sinus rhythm, normal intervals, normal ST segments
	Summary		All 4 ECGS had similar interpretation	Both 12 and single lead ECGs had similar interpretation	All 4 ECGS had similar interpretation

Volunteer 2_KAUST_Single lead ECG system

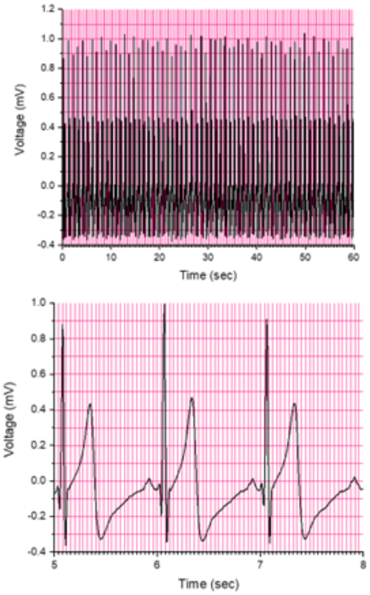

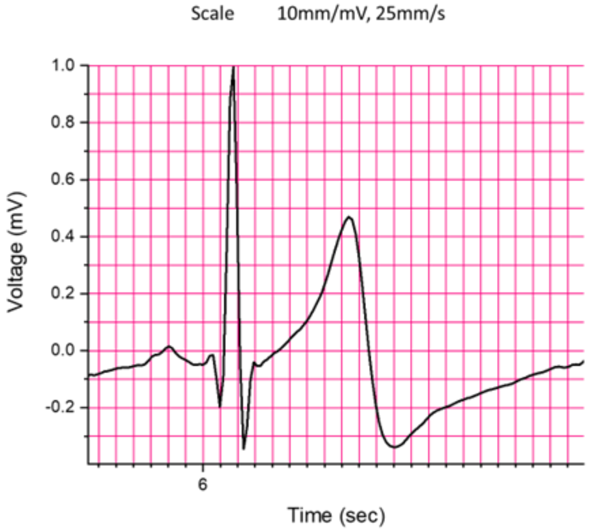

Volunteer 2_Commercial_Single lead ECG system

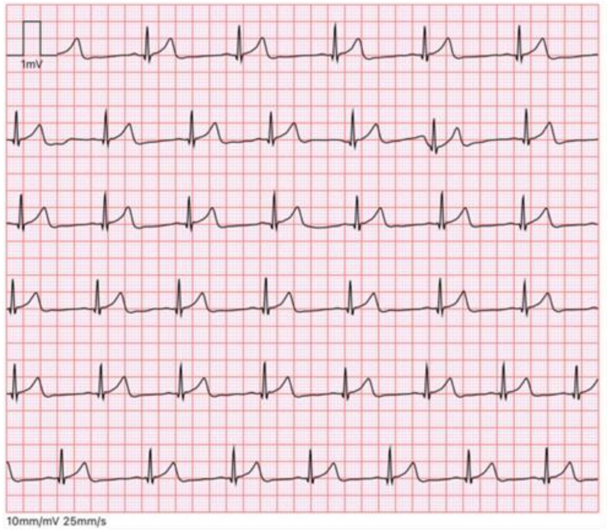

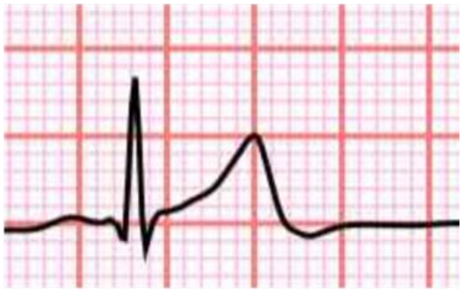

Figure S14a: Volunteer 2, KAUST single lead and commercial single-lead ECG.

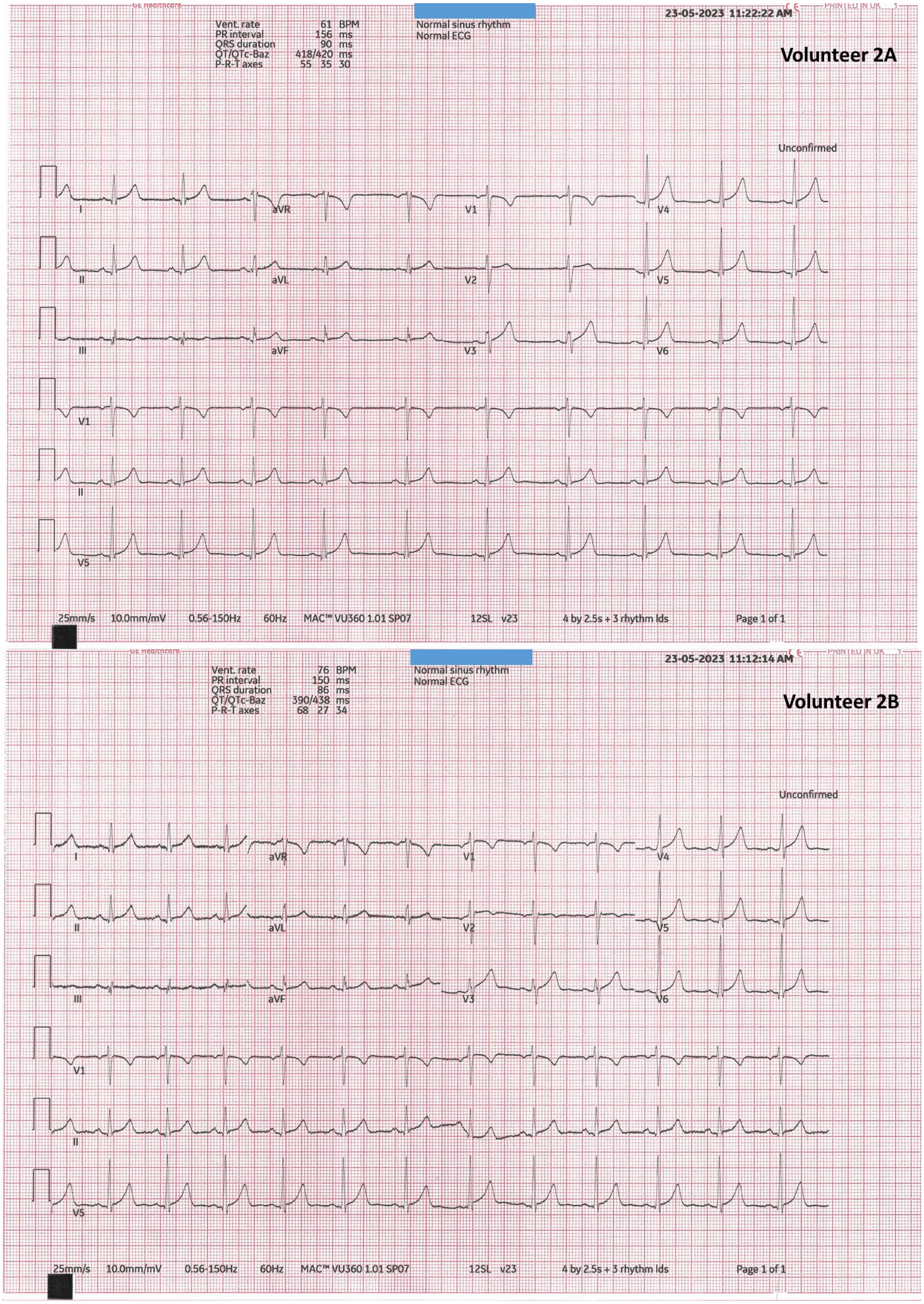

Figure S14b: Volunteer 2: Blinded review of 12 lead ECG using commercial ECG electrodes and gentle-to-skin screen printed ECG electrodes. The sample identification was hidden from the reviewers using a blue color square.

Volunteer No	ECG Type	Leads	Reviewer 1 (Cardiology Consultant)	Reviewer 2 (Cardiology Consultant)	Reviewer 3 (FM Consultant)
**V3**	A	12	Normal sinus rhythm, normal intervals	Sinus arrhythmia<br>Normal intervals and segments	Sinus arrhythmia with inverted T wave in V2
	B	12	Normal sinus rhythm, normal intervals	Sinus arrhythmia<br>Normal intervals and segments	Sinus arrhythmia with inverted T wave in V2
	KAUST	1	Normal sinus rhythm, normal intervals	Sinus arrhythmia<br>Non-specefic ST-T changes. Inverted T wave.	Sinus arrhythmia<br>Normal intervals and segments
	Commercial	1	Normal sinus rhythm, normal intervals	Sinus arrhythmia<br>Non-specefic ST-T changes. Inverted T wave.	Sinus arrhythmia<br>Normal intervals and segments
	Summary		All 4 ECGS had similar interpretation	Both 12 and single lead ECGs had similar interpretation	Both 12 and single lead ECGs had similar interpretation

Volunteer 3_KAUST_Single lead ECG system

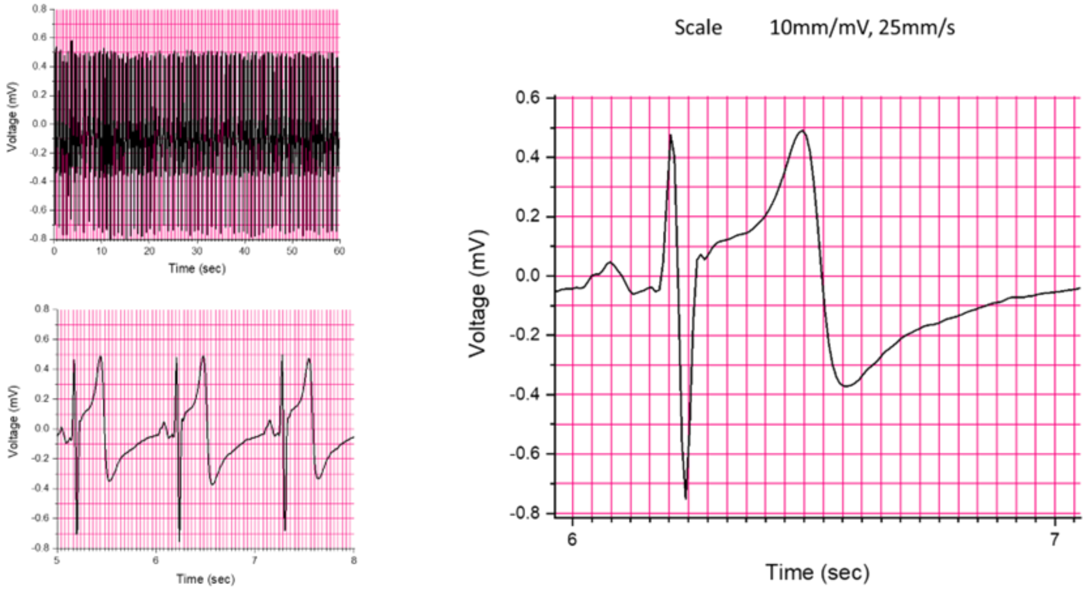

Volunteer 3_Commercial_Single lead ECG system

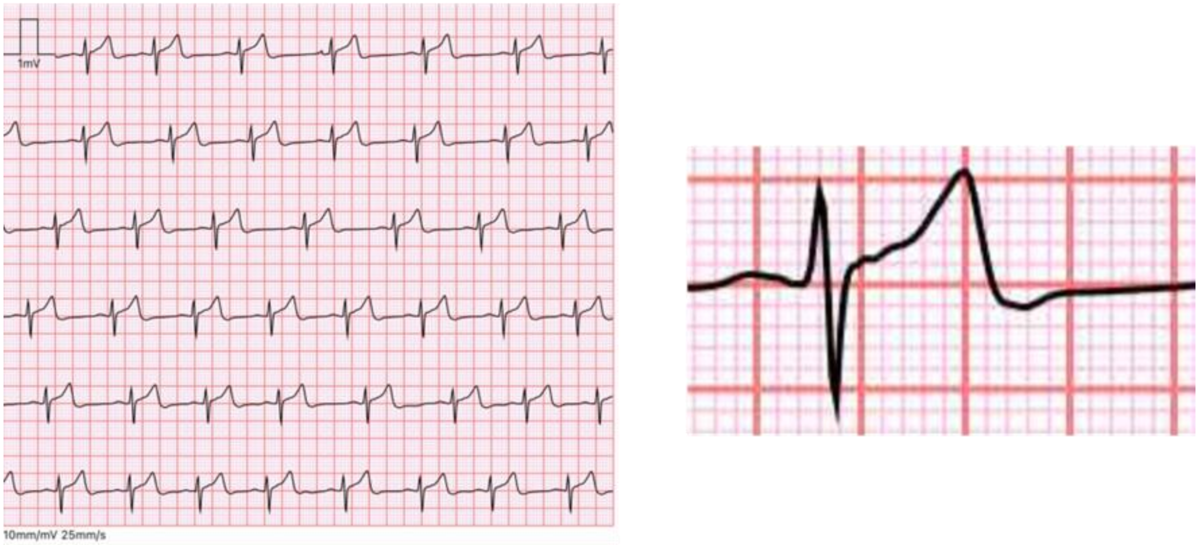

Figure S15a: Volunteer 3, KAUST single lead and commercial single-lead ECG.

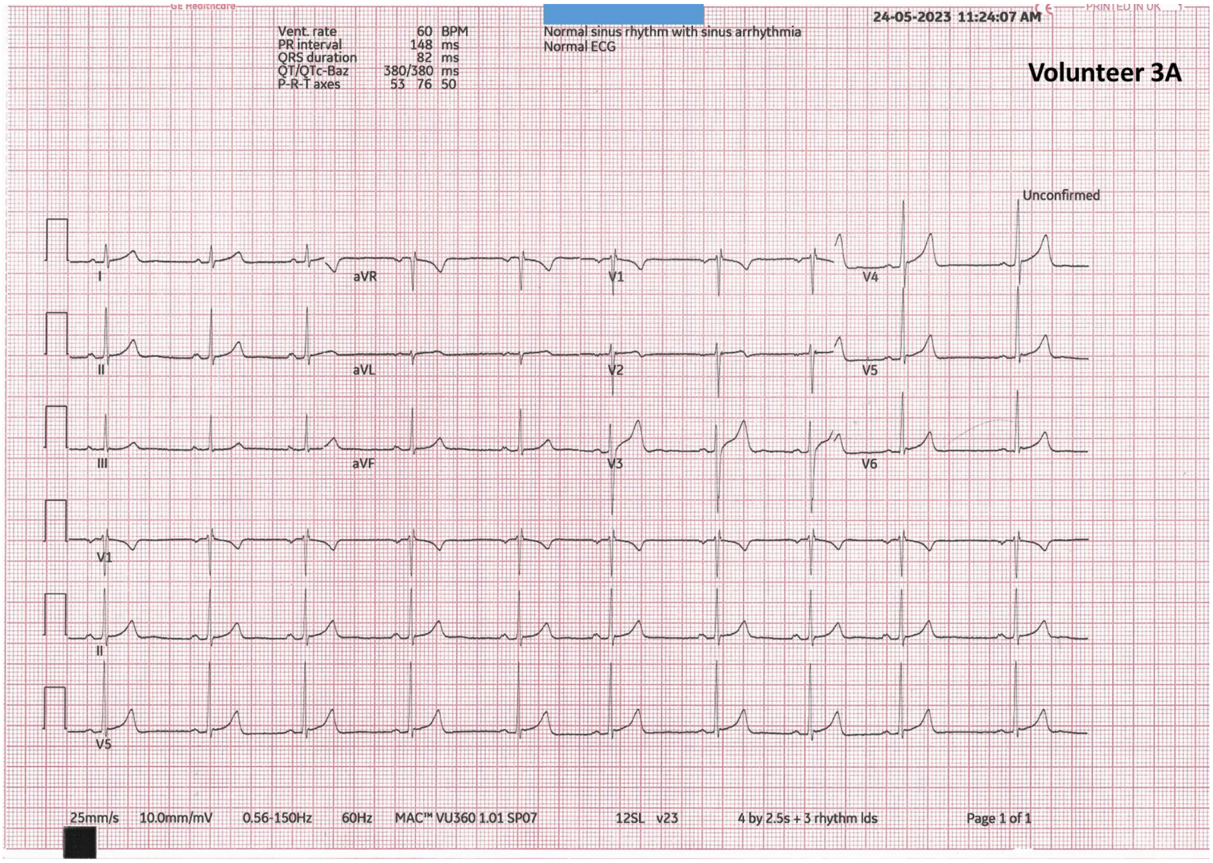

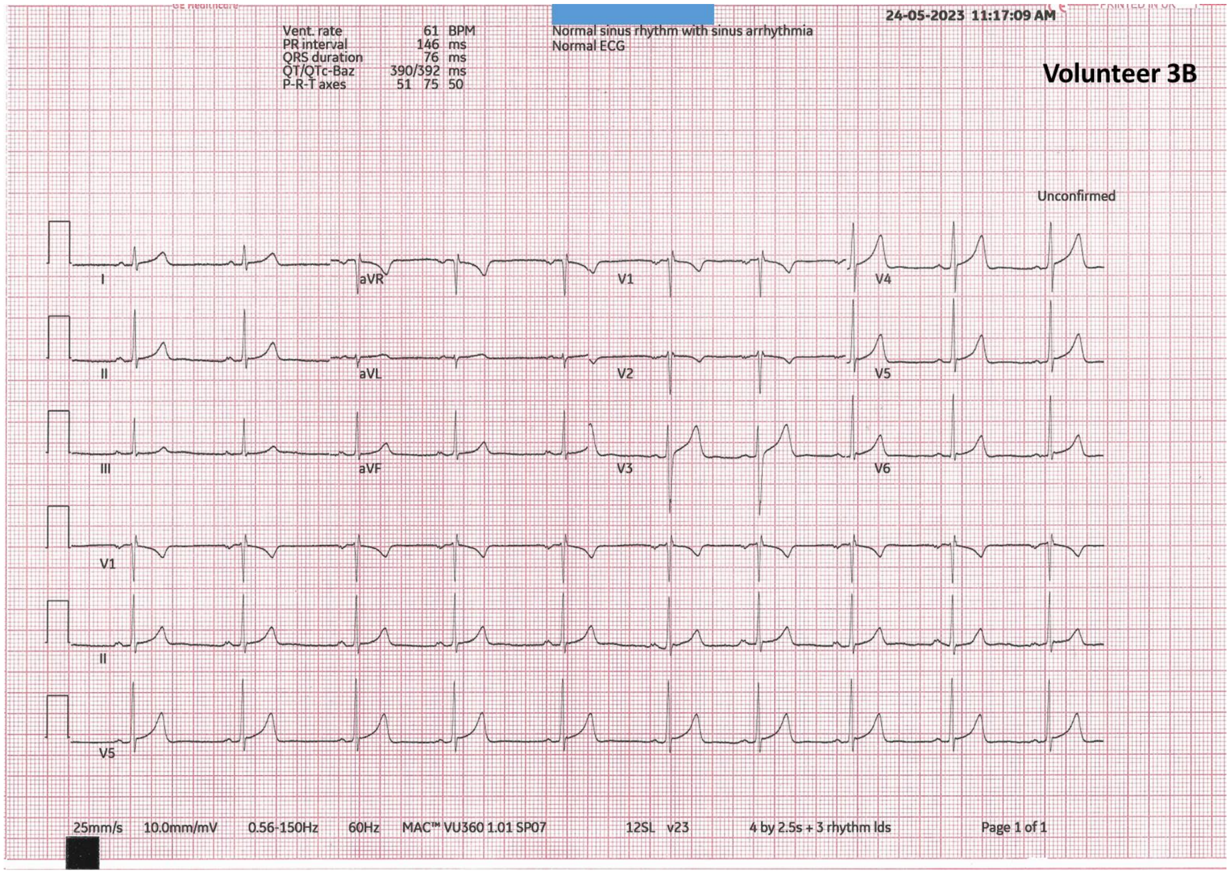

Figure S15b: Volunteer 3: Blinded review of 12 lead ECG using commercial ECG electrodes and gentle-to-skin screen printed ECG electrodes. The sample identification was hidden from the reviewers using a blue color square.

Volunteer No	ECG Type	Leads	Reviewer 1 (Cardiology Consultant)	Reviewer 2 (Cardiology Consultant)	Reviewer 3 (FM Consultant)
V4	A	12	Normal sinus rhythm, normal intervals	Normal sinus rhythm, normal intervals, normal ST segments	Normal sinus rhythm, normal intervals, normal ST segments
	B	12	Normal sinus rhythm, normal intervals	Normal sinus rhythm, normal intervals, normal ST segments	Normal sinus rhythm, normal intervals, normal ST segments
	KAUST	1	Normal sinus rhythm, normal intervals	Normal SR, ST-T changes, T wave inversion	Normal sinus rhythm, normal intervals, normal ST segments
	Commercial	1	Normal sinus rhythm, normal intervals	Normal SR, ST-T changes, T wave inversion	Normal sinus rhythm, normal intervals, normal ST segments
	Summary		All 4 ECGS had similar interpretation	Both 12 and single lead ECGs had similar interpretation	All 4 ECGS had similar interpretation

Volunteer 4_KAUST_Single lead ECG system

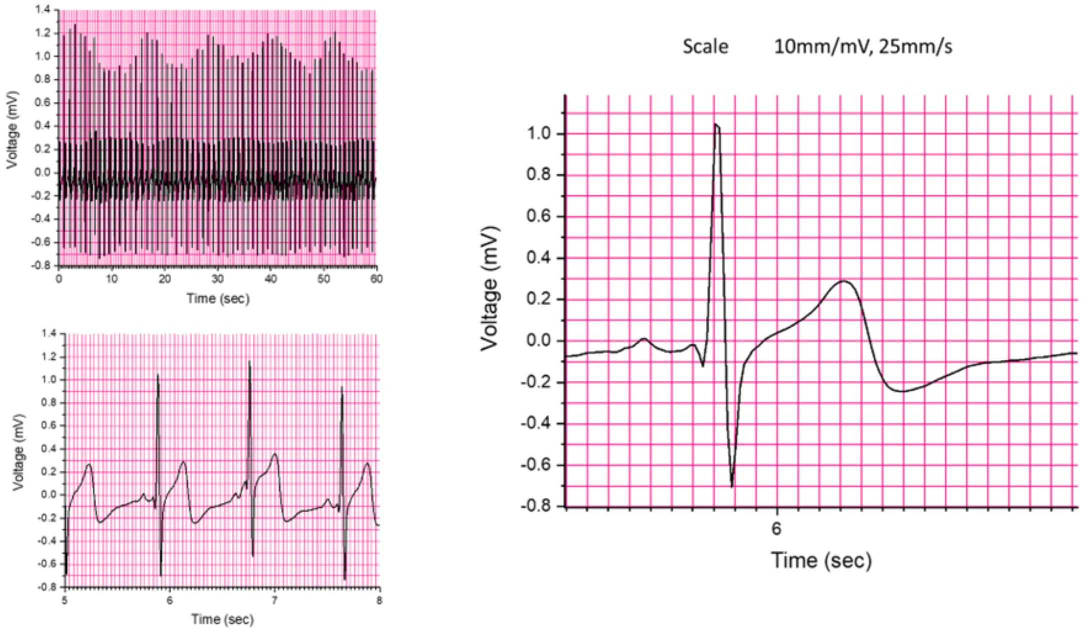

Volunteer 4_Commercial_Single lead ECG system

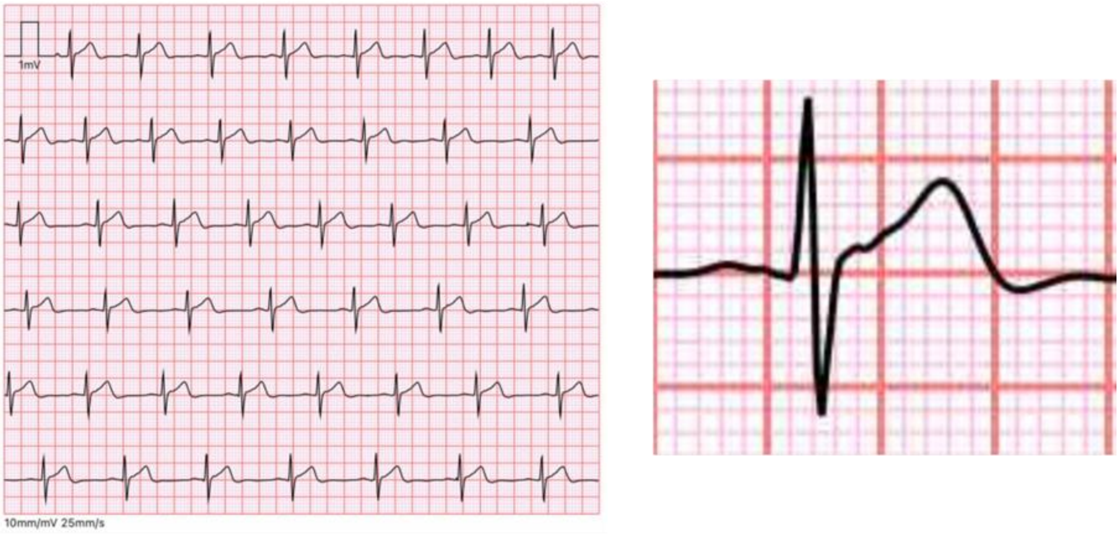

Figure S16a: Volunteer 4, KAUST single lead, and commercial single-lead ECG.

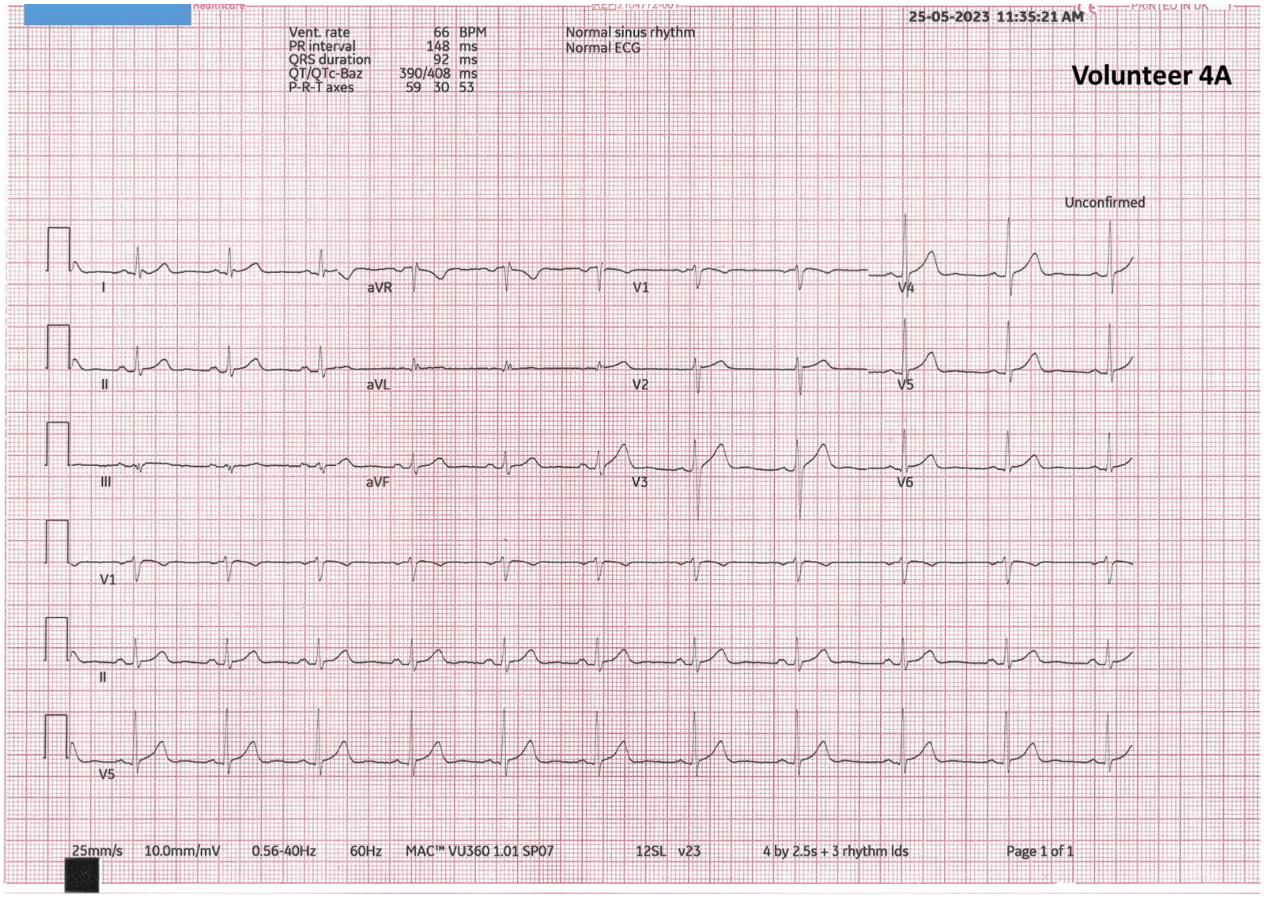

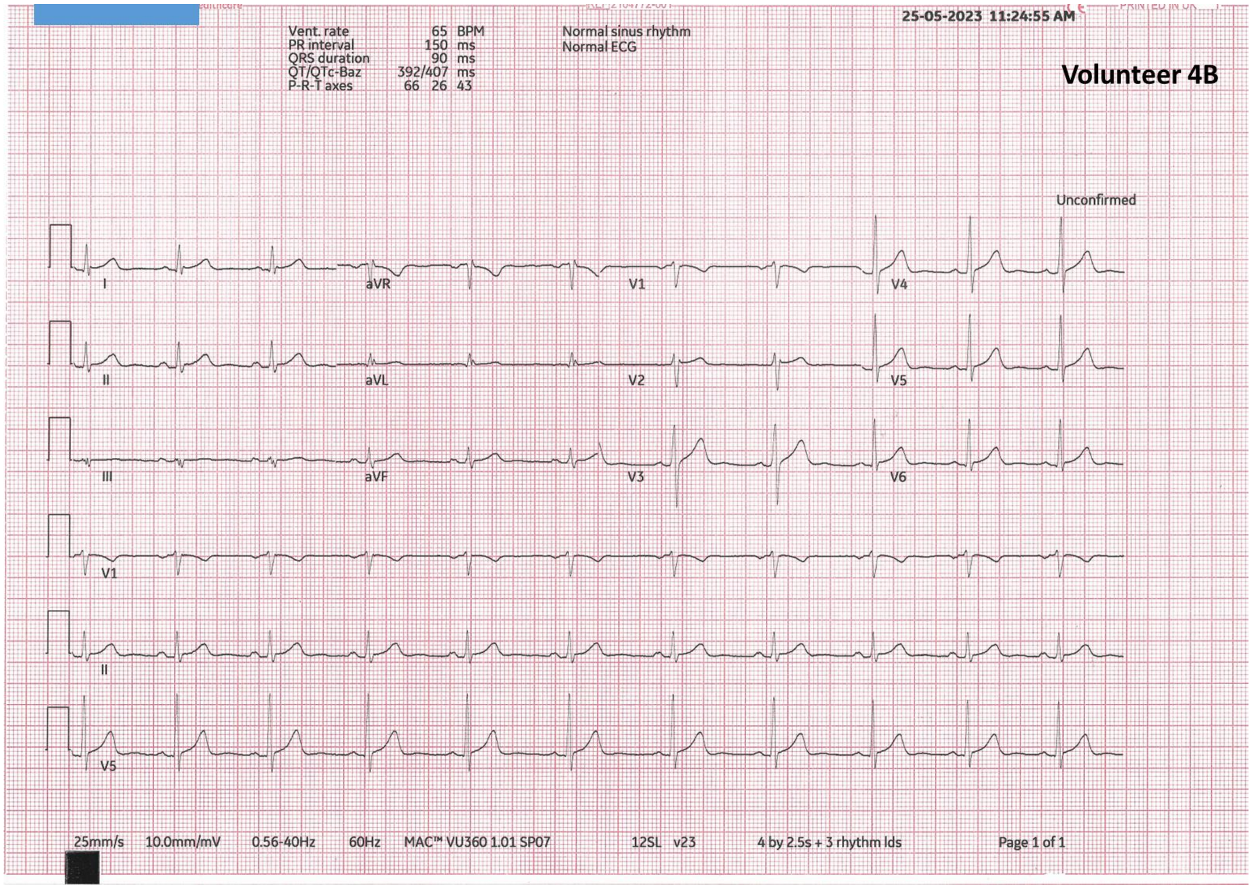

Figure S16b: Volunteer 4: Blinded review of 12 lead ECG using commercial ECG electrodes and gentle-to-skin screen printed ECG electrodes. The sample identification was hidden from the reviewers using a blue color square.

Volunteer No	ECG Type	Leads	Reviewer 1 (Cardiology Consultant)	Reviewer 2 (Cardiology Consultant)	Reviewer 3 (FM Consultant)
V5	A	12	Normal sinus rhythm, normal intervals, early repolarization pattern	Normal sinus rhythm, normal intervals, normal ST segments	Normal sinus rhythm, normal intervals, early repolarization pattern
	B	12	Normal sinus rhythm, normal intervals, early repolarization pattern	Normal sinus rhythm, normal intervals, normal ST segments	Normal sinus rhythm, normal intervals, early repolarization pattern
	KAUST	1	Normal sinus rhythm, normal intervals, early repolarization pattern	Normal sinus rhythm, ST depression, normal T wave	Normal sinus rhythm, normal intervals, early repolarization pattern
	Commercial	1	Normal sinus rhythm, normal intervals, early repolarization pattern	Normal sinus rhythm, ST depression, normal T wave	Normal sinus rhythm, normal intervals, early repolarization pattern
	Summary		All 4 ECGS had similar interpretation	Both 12 and single lead ECGs had similar interpretation	All 4 ECGS had similar interpretation

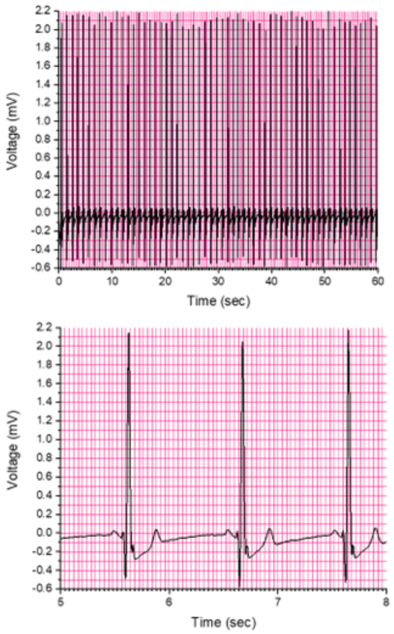

Volunteer 5_KAUST_Single lead ECG system

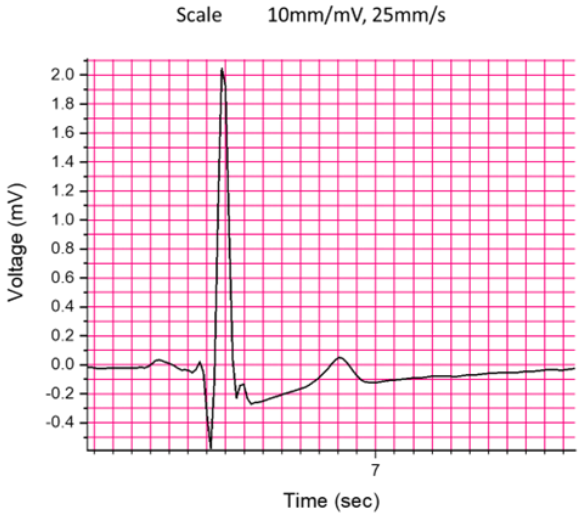

Volunteer 5_Commercial_Single lead ECG system

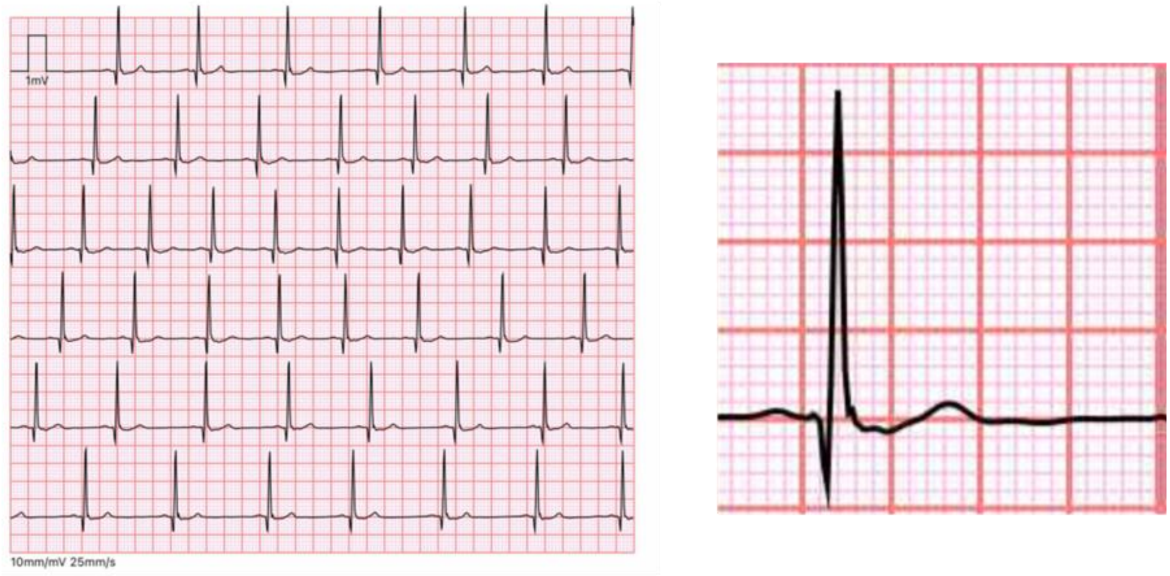

Figure S17a: Volunteer 5, KAUST single lead and commercial single-lead ECG.

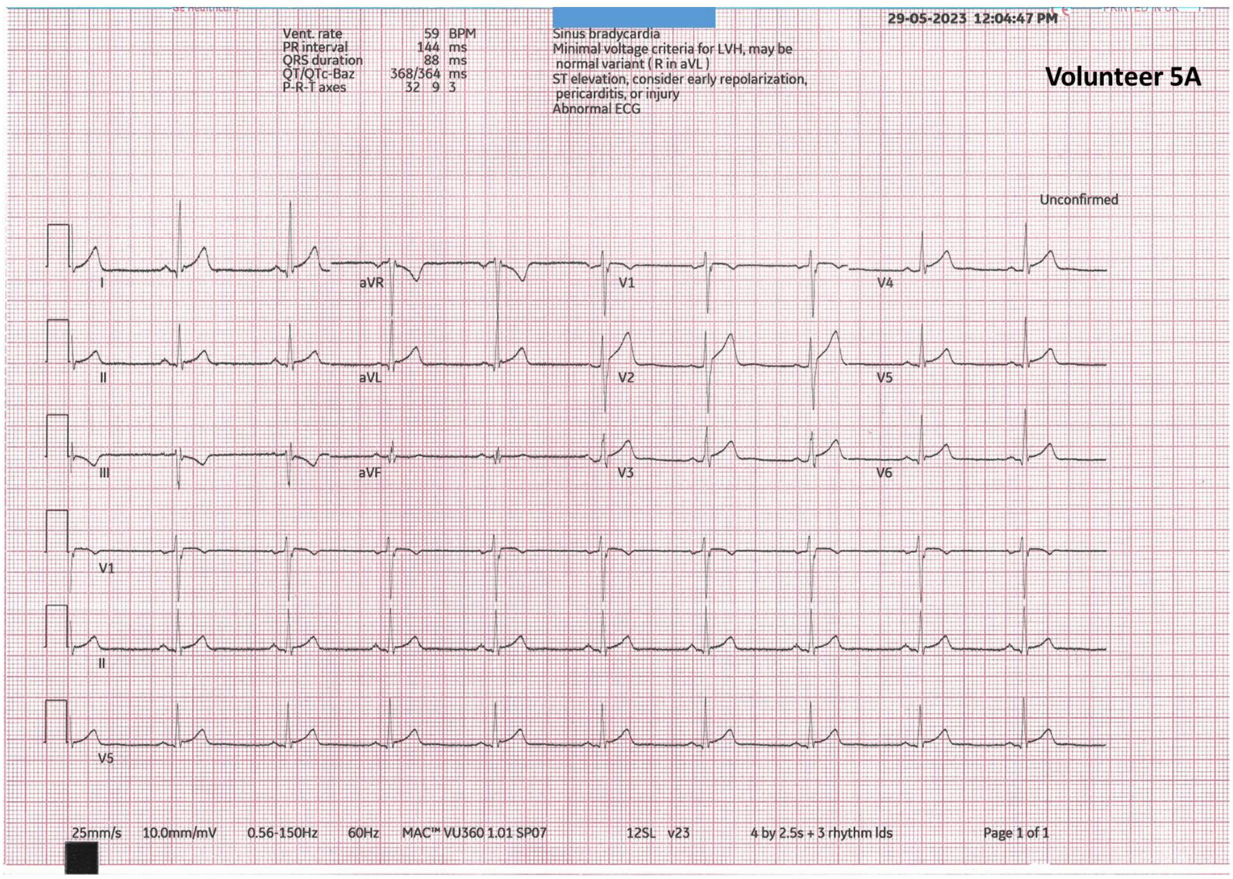

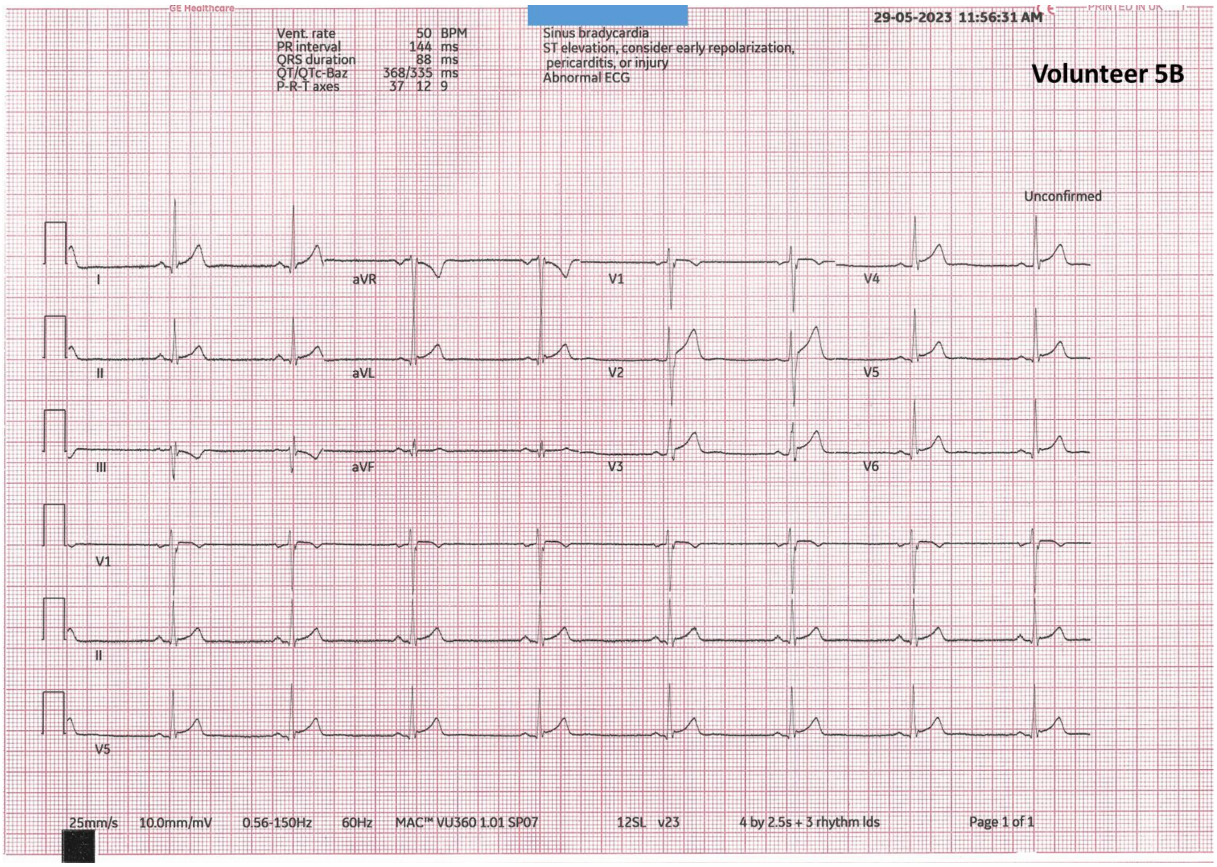

Figure S17b: Volunteer 5: Blinded review of 12 lead ECG using commercial ECG electrodes and gentle-to-skin screen printed ECG electrodes. The sample identification was hidden from the reviewers using a blue color square.

Volunteer No	ECG Type	Leads	Reviewer 1 (Cardiology Consultant)	Reviewer 2 (Cardiology Consultant)	Reviewer 3 (FM Consultant)
V6	A	12	Normal sinus rhythm, normal intervals, normal ST segments	Normal sinus rhythm, normal intervals, normal ST segments	Normal sinus rhythm, normal intervals, normal ST segments
	B	12	Normal sinus rhythm, normal intervals, normal ST segments	Normal sinus rhythm, normal intervals, normal ST segments	Normal sinus rhythm, normal intervals, normal ST segments
	KAUST	1	Normal sinus rhythm, normal intervals, normal ST segments	Normal sinus rhythm, normal intervals, normal ST segments	Normal sinus rhythm, normal intervals, normal ST segments
	Commercial	1	Normal sinus rhythm, normal intervals, normal ST segments	Normal sinus rhythm, normal intervals, normal ST segments	Normal sinus rhythm, normal intervals, normal ST segments
	Summary		All 4 ECGS had similar interpretation	All 4 ECGS had similar interpretation	All 4 ECGS had similar interpretation

Volunteer 6_KAUST_Single lead ECG system

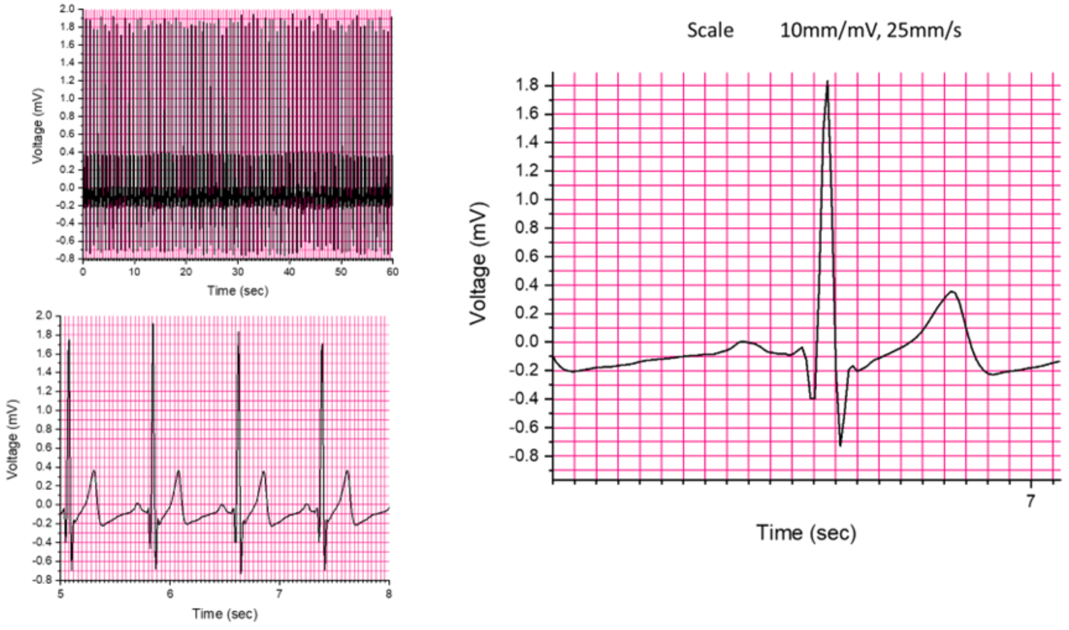

Volunteer 6_Commercial_Single lead ECG system

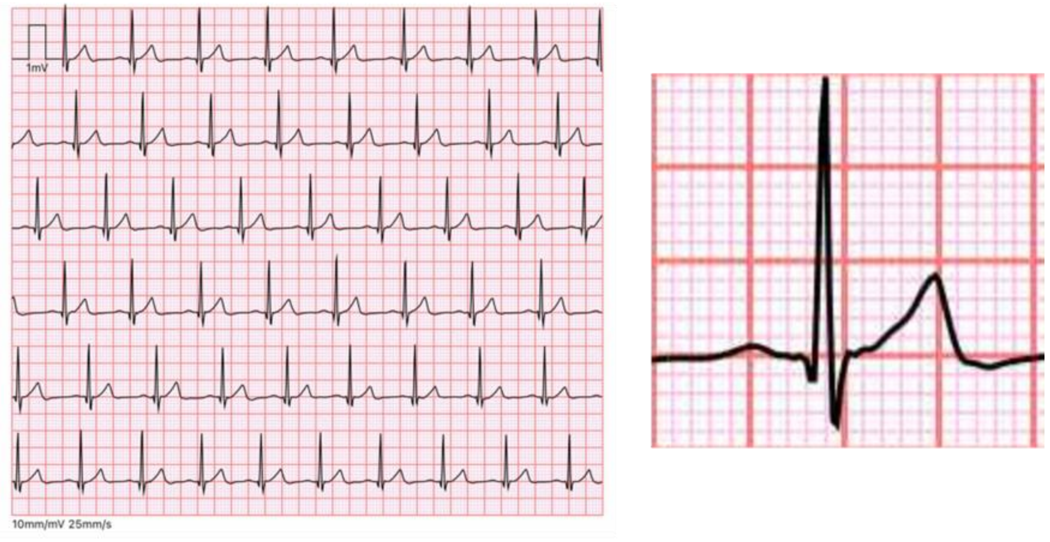

Figure S18a: Volunteer 6, KAUST single lead, and commercial single-lead ECG.

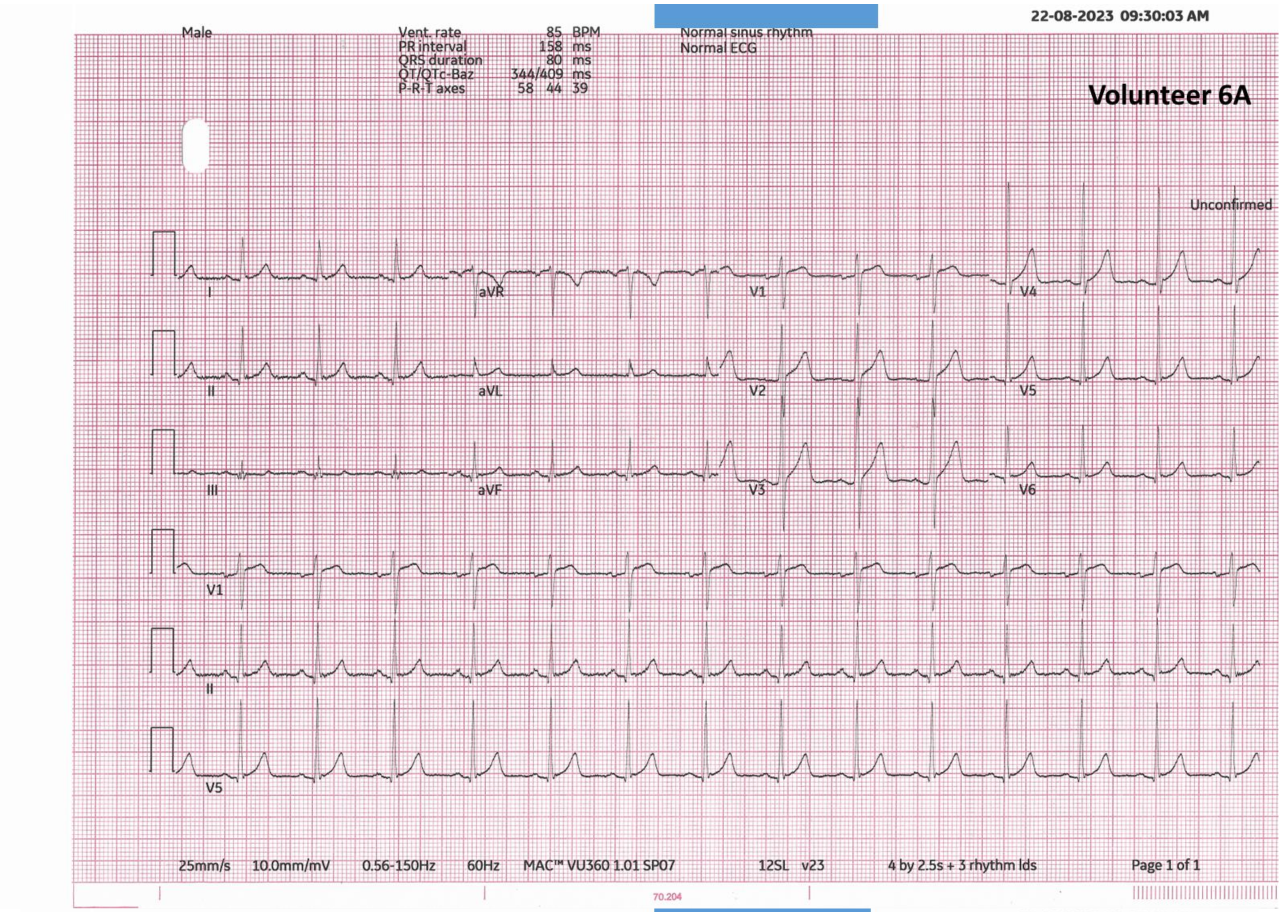

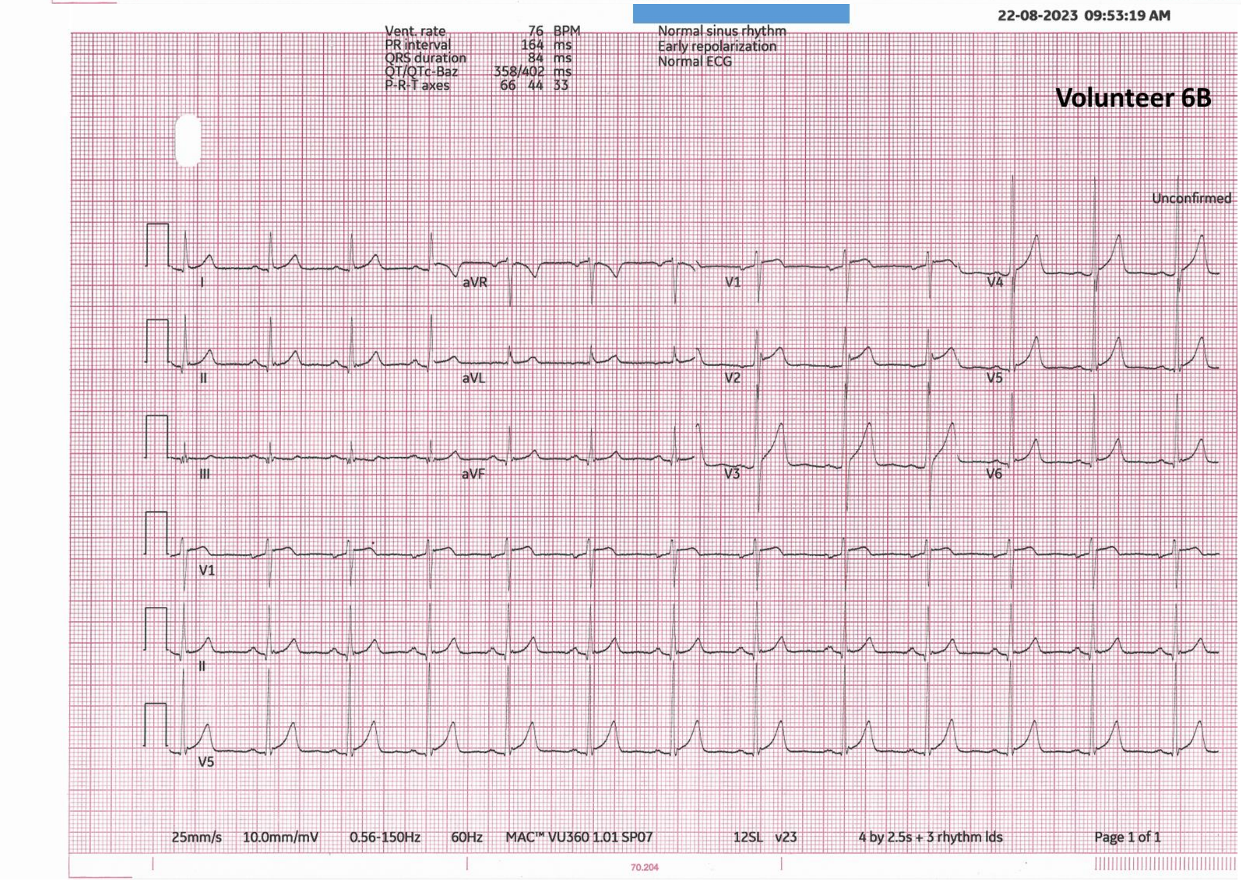

Figure S18b: Volunteer 6: Blinded review of 12 lead ECG using commercial ECG electrodes and gentle-to-skin screen printed ECG electrodes. The sample identification was hidden from the reviewers using a blue color square.

Volunteer No	ECG Type	Leads	Reviewer 1 (Cardiology Consultant)	Reviewer 2 (Cardiology Consultant)	Reviewer 3 (FM Consultant)
V7	A	12	Normal sinus rhythm, normal intervals	Normal sinus rhythm, normal intervals	Normal sinus rhythm, normal intervals
	B	12	Normal sinus rhythm, normal intervals	Normal sinus rhythm, normal intervals	Normal sinus rhythm, normal intervals
	KAUST	1	Normal sinus rhythm, normal intervals	Normal sinus rhythm, ST segment elevation, T wave inversion	Normal sinus rhythm, normal intervals
	Commercial	1	Normal sinus rhythm, normal intervals	Normal sinus rhythm, ST segment elevation, T wave inversion	Normal sinus rhythm, normal intervals
	Summary		All 4 ECGS had similar interpretation	Both 12 and single lead ECGs had similar interpretation	All 4 ECGS had similar interpretation

Volunteer 7_KAUST_Single lead ECG system

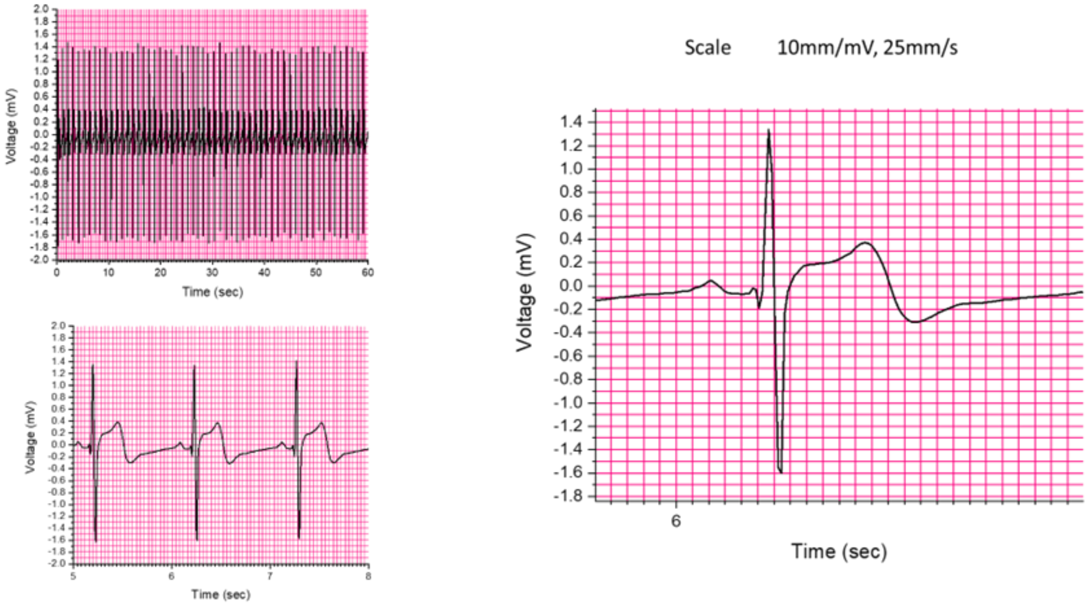

Volunteer 7_Commercial_Single lead ECG system

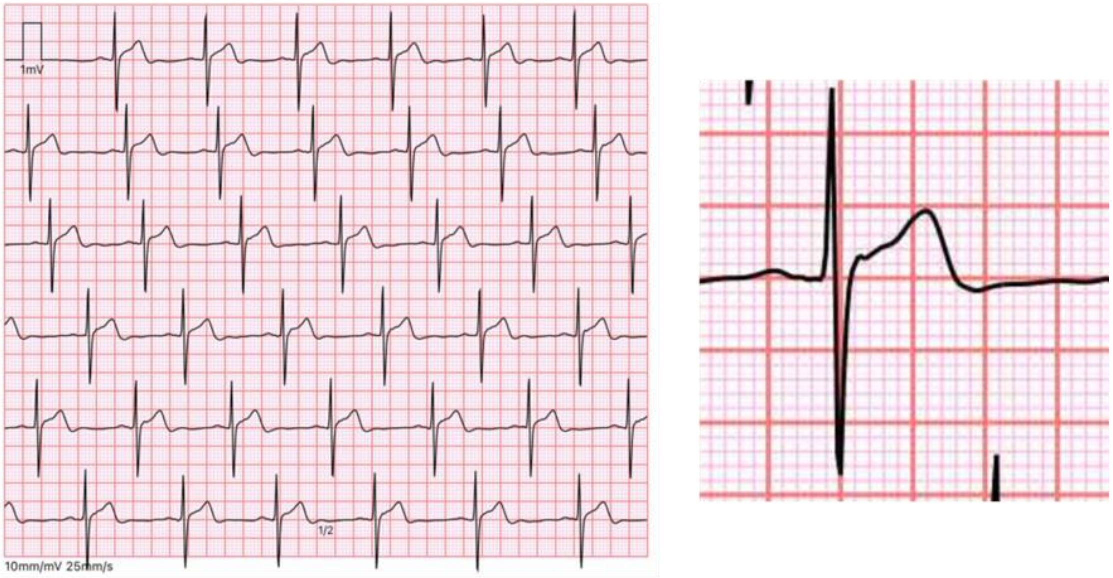

Figure S19a: Volunteer 7, KAUST single lead, and commercial single-lead ECG.

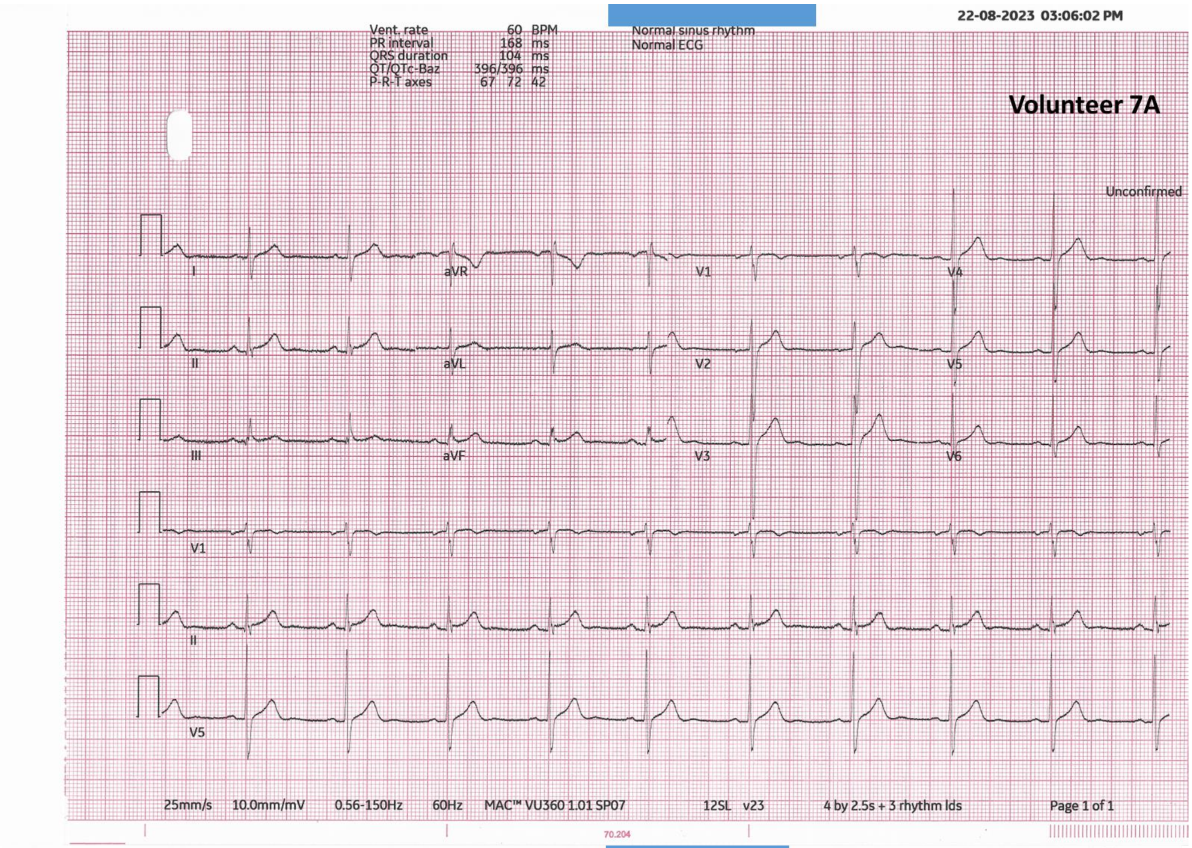

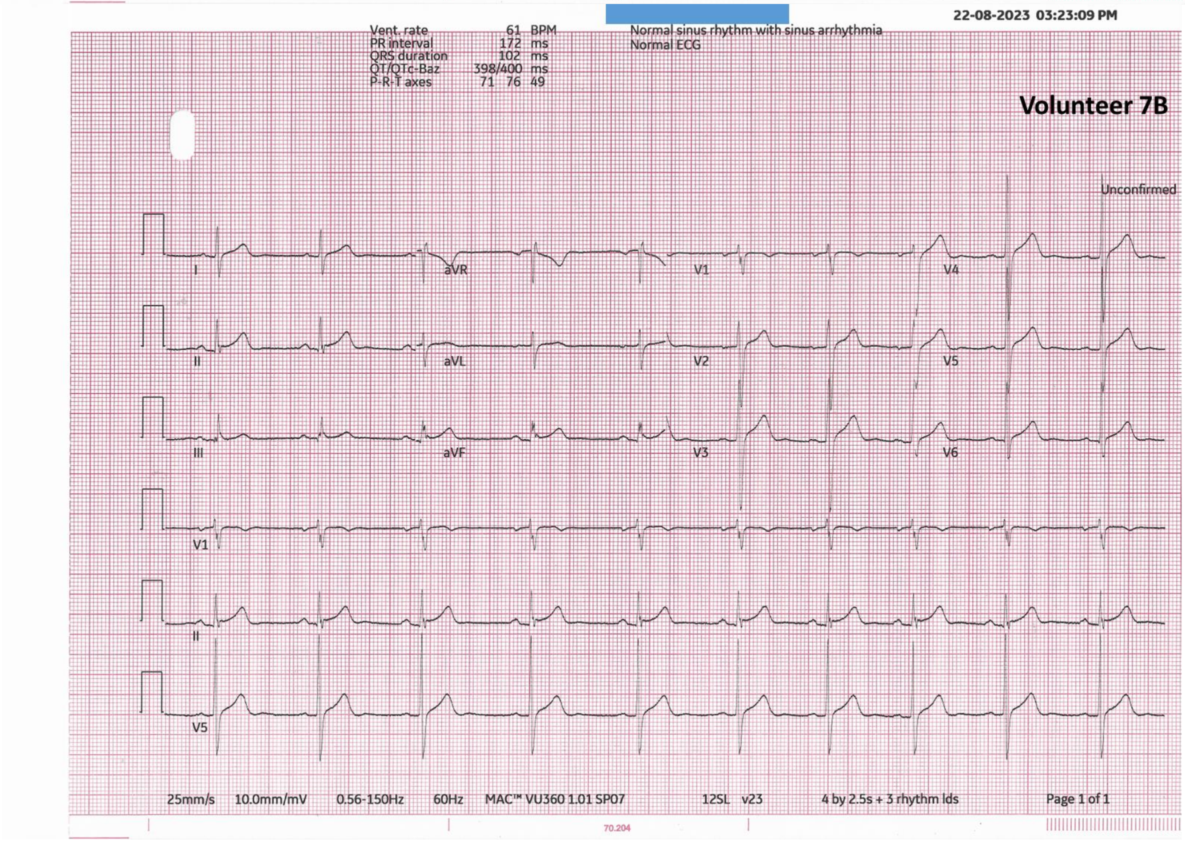

Figure S19b: Volunteer 7: Blinded review of 12 lead ECG using commercial ECG electrodes and gentle-to-skin screen printed ECG electrodes. The sample identification was hidden from the reviewers using a blue color square.

Volunteer No	ECG Type	Leads	Reviewer 1 (Cardiology Consultant)	Reviewer 2 (Cardiology Consultant)	Reviewer 3 (FM Consultant)
V8	A	12	Normal sinus rhythm, normal intervals, early repolarization pattern	Normal sinus rhythm, inferior Q waves, normal intervals and segments	Normal sinus rhythm, normal intervals, early repolarization pattern
	B	12	Normal sinus rhythm, normal intervals, early repolarization pattern	Normal sinus rhythm, inferior Q waves, normal intervals and segments	Normal sinus rhythm, normal intervals, early repolarization pattern
	KAUST	1	Normal sinus rhythm, normal intervals, early repolarization pattern	Normal sinus rhythm, ST changes, T wave inversion	Normal sinus rhythm, normal intervals, early repolarization pattern
	Commercial	1	Normal sinus rhythm, normal intervals, early repolarization pattern	Normal sinus rhythm, ST changes, T wave inversion	Normal sinus rhythm, normal intervals, early repolarization pattern
	Summary		All 4 ECGS had similar interpretation	Both 12 and single lead ECGs had similar interpretation	All 4 ECGS had similar interpretation

Volunteer 8_KAUST_Single lead ECG system

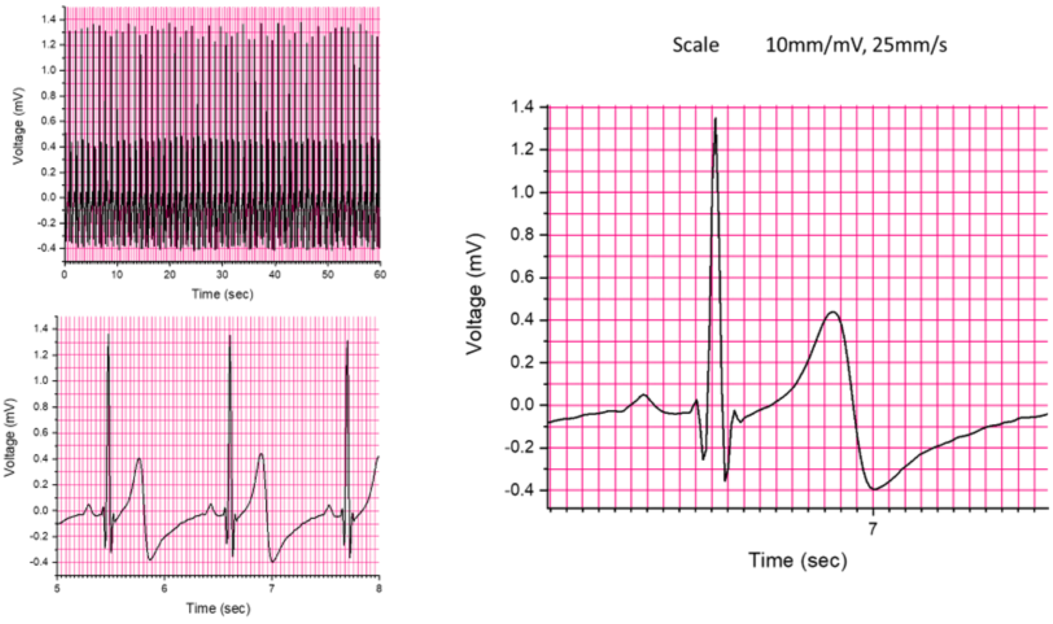

Volunteer 8_Commercial_Single lead ECG system

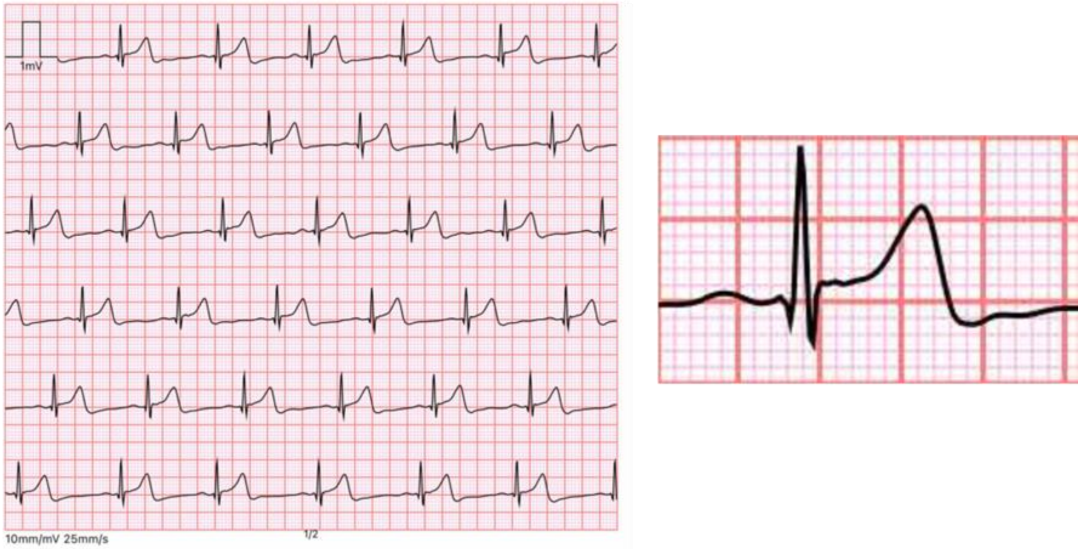

Figure S20a: Volunteer 8, KAUST single lead, and commercial single-lead ECG.

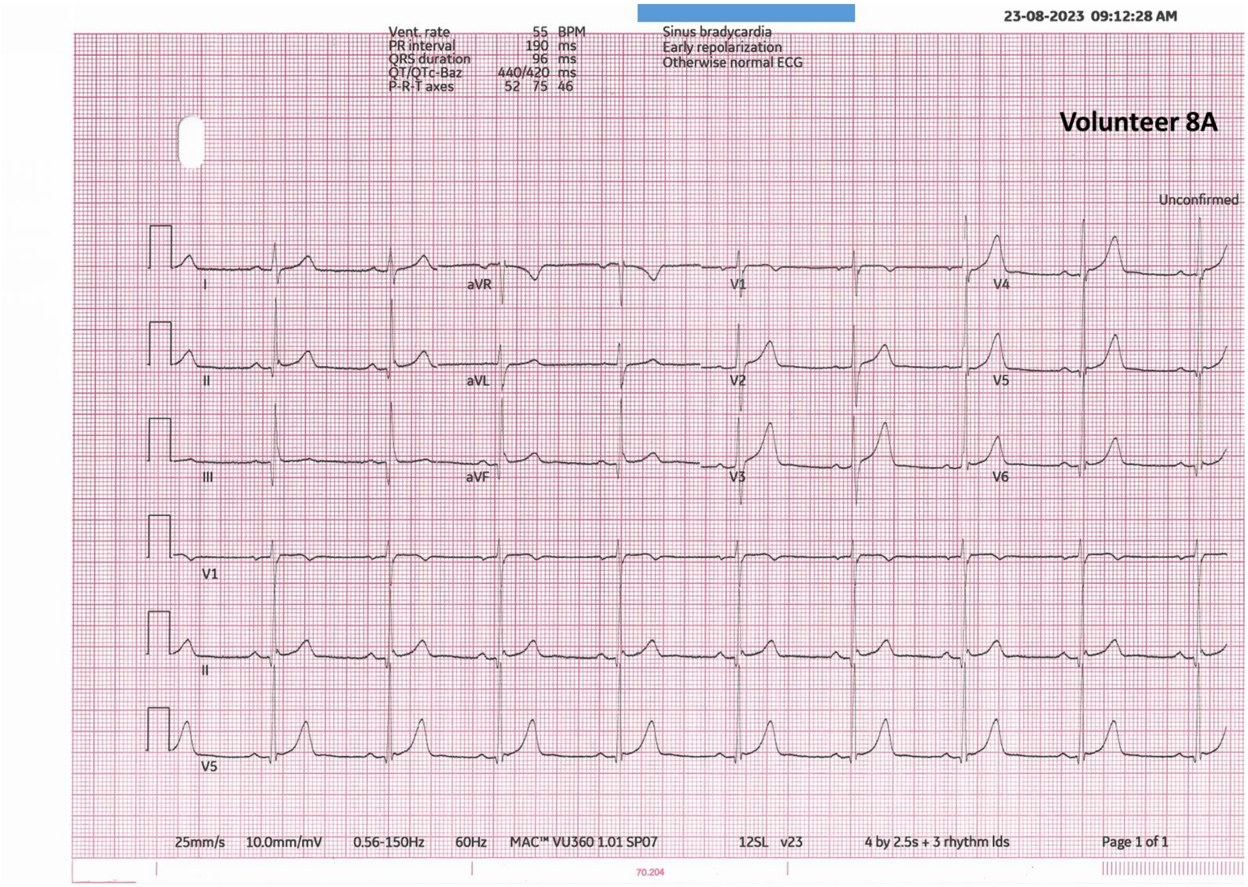

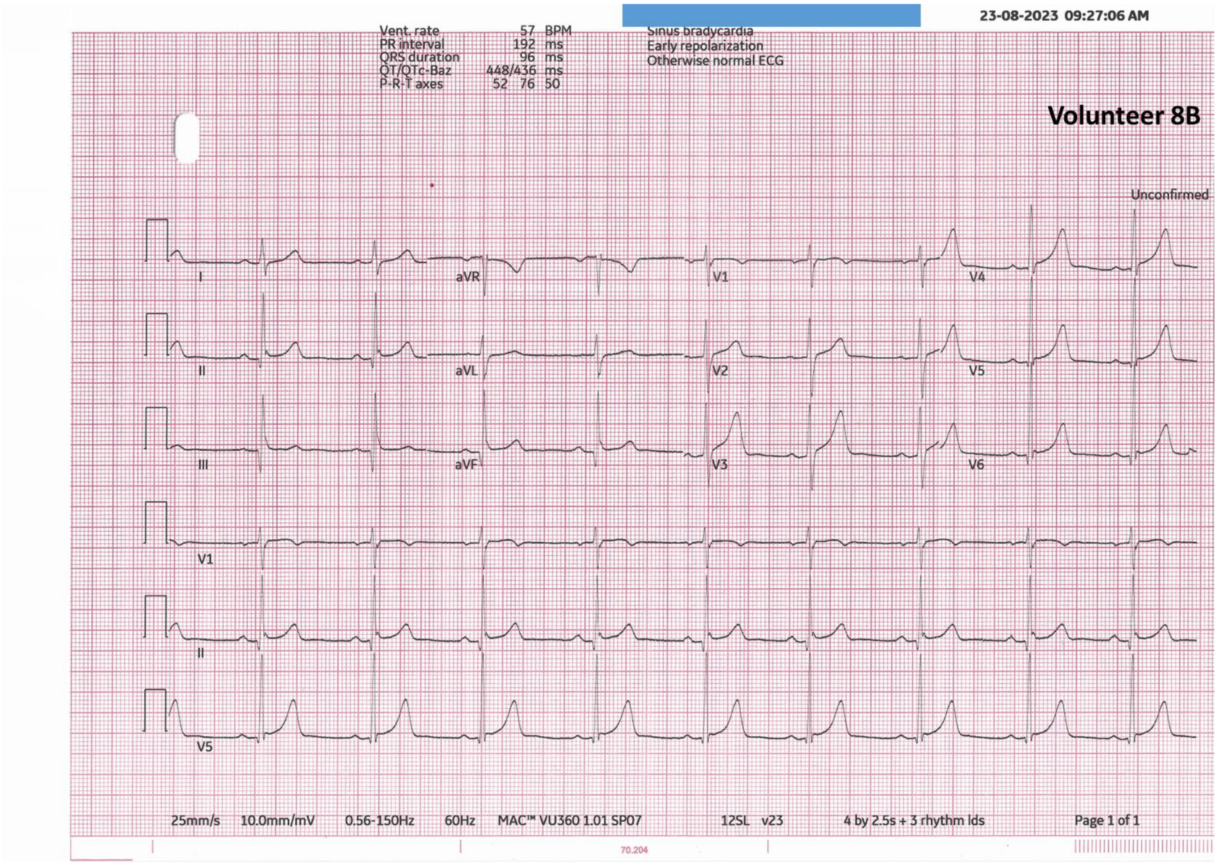

Figure S20b: Volunteer 8: Blinded review of 12 lead ECG using commercial ECG electrodes and gentle-to-skin screen printed ECG electrodes. The sample identification was hidden from the reviewers using a blue color square.

Volunteer No	ECG Type	Leads	Reviewer 1 (Cardiology Consultant)	Reviewer 2 (Cardiology Consultant)	Reviewer 3 (FM Consultant)
V9	A	12	Normal sinus rhythm, normal intervals, normal ST segments	Normal sinus rhythm, normal intervals, normal ST segments	Normal sinus rhythm, normal intervals, normal ST segments
	B	12	Normal sinus rhythm, normal intervals, normal ST segments	Normal sinus rhythm, normal intervals, normal ST segments	Normal sinus rhythm, normal intervals, normal ST segments
	KAUST	1	Normal sinus rhythm, normal intervals, normal ST segments	Normal sinus rhythm, Non specific ST - T changes	Normal sinus rhythm, normal intervals, normal ST segments
	Commercial	1	Normal sinus rhythm, normal intervals, normal ST segments	Normal sinus rhythm, Non specific ST - T changes	Normal sinus rhythm, normal intervals, normal ST segments
	Summary		All 4 ECGS had similar interpretation	Both 12 and single lead ECGs had similar interpretation	All 4 ECGS had similar interpretation

Volunteer 9_KAUST_Single lead ECG system

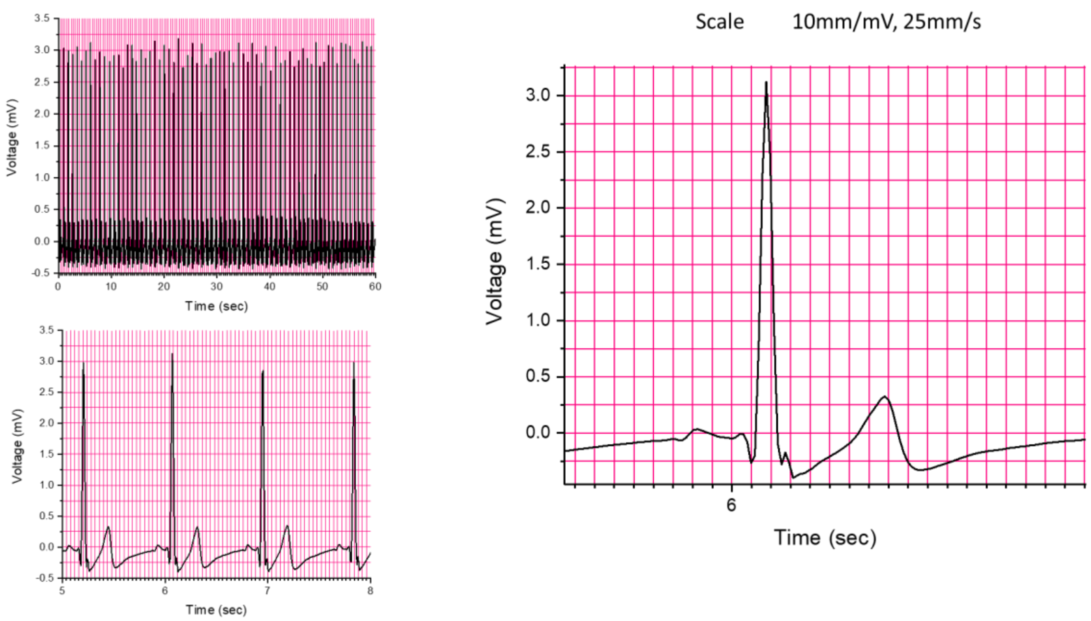

Volunteer 9_Commercial_Single lead ECG system

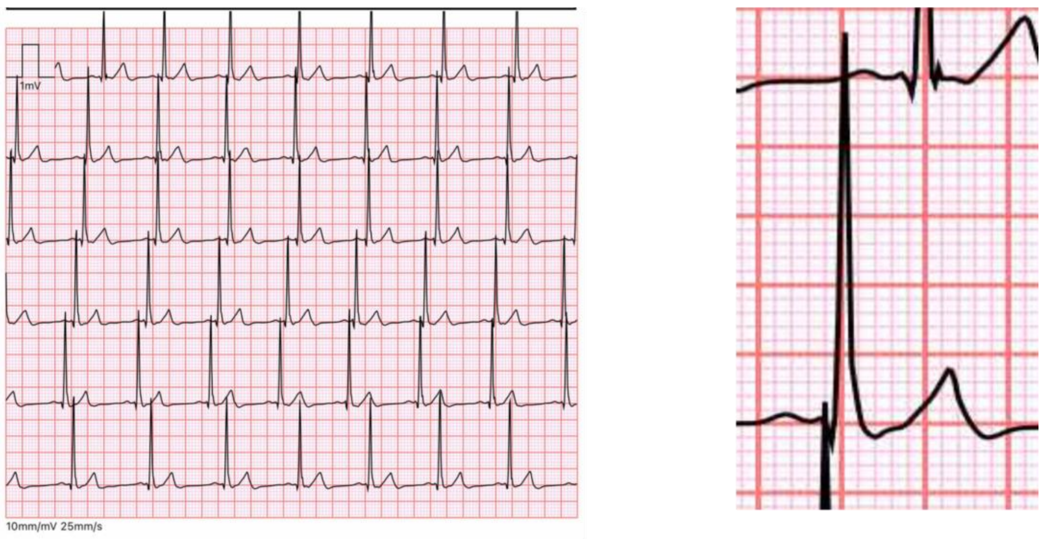

Figure S21a: Volunteer 9, KAUST single lead, and commercial single-lead ECG.

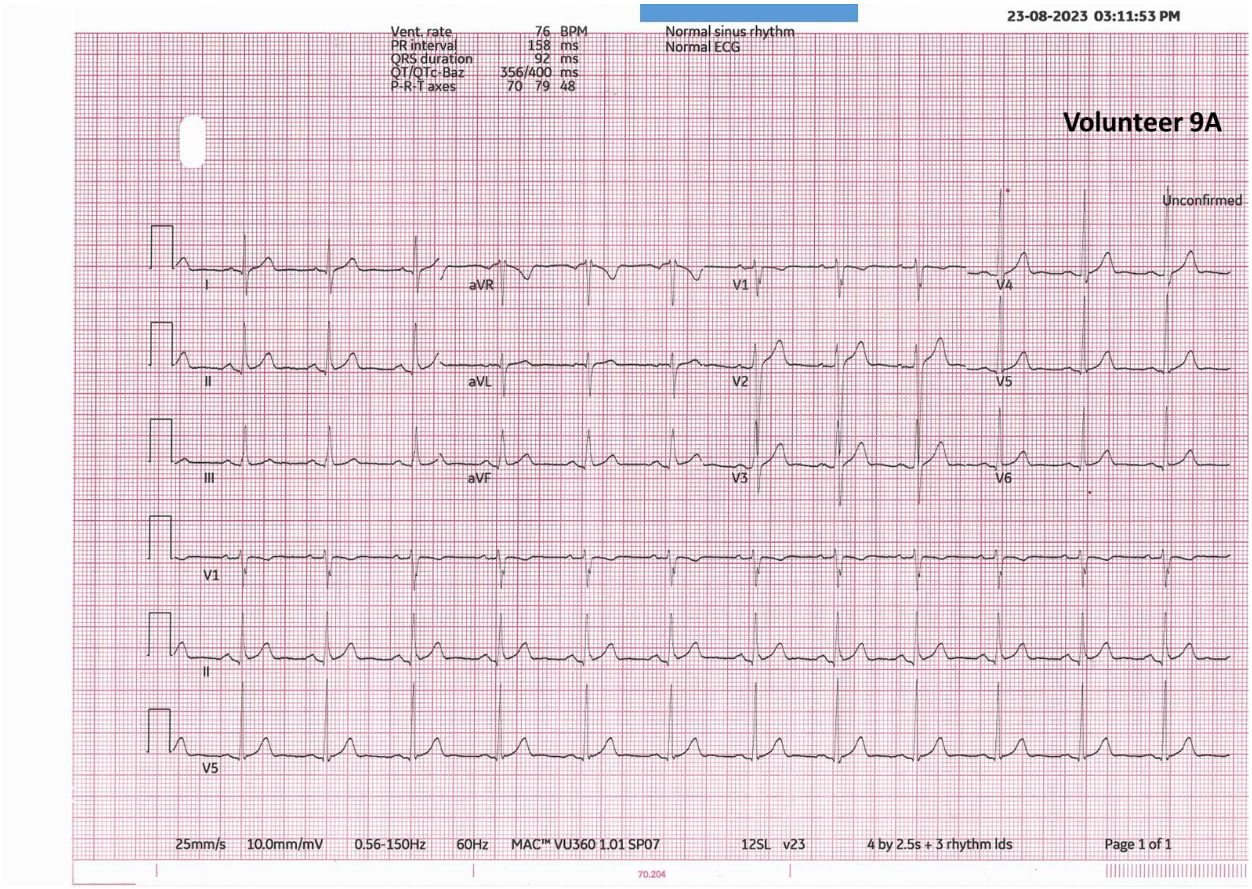

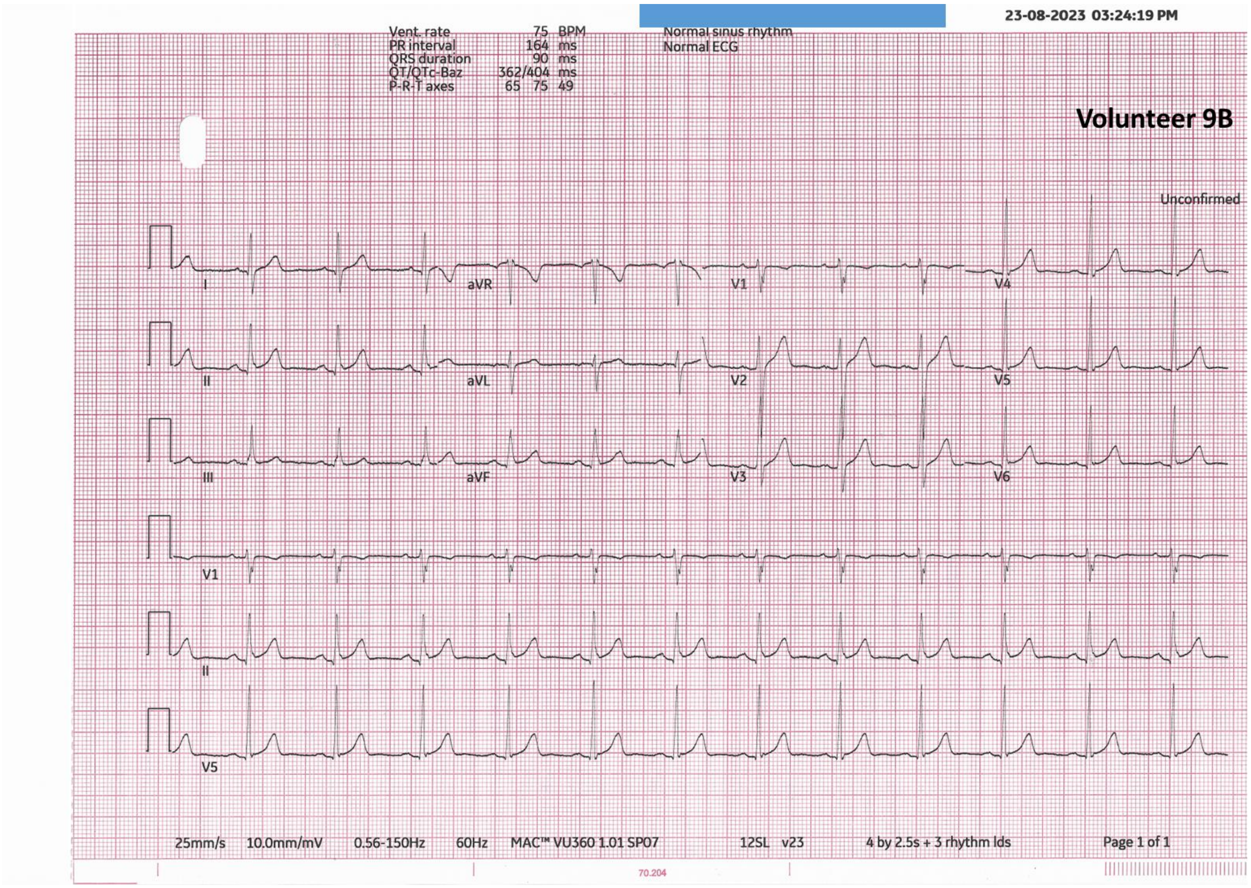

Figure S21b: Volunteer 9: Blinded review of 12 lead ECG using commercial ECG electrodes and gentle-to-skin screen printed ECG electrodes. The sample identification was hidden from the reviewers using a blue color square.

Volunteer No	ECG Type	Leads	Reviewer 1 (Cardiology Consultant)	Reviewer 2 (Cardiology Consultant)	Reviewer 3 (FM Consultant)
V10	A	12	Normal sinus rhythm, normal intervals, early repolarization pattern	Normal sinus rhythm, normal intervals, normal ST segments	Normal sinus rhythm, normal intervals, early repolarization pattern
	B	12	Normal sinus rhythm, normal intervals, early repolarization pattern	Normal sinus rhythm, normal intervals, normal ST segments	Normal sinus rhythm, normal intervals, early repolarization pattern
	KAUST	1	Normal sinus rhythm, normal intervals, early repolarization pattern	Normal sinus rhythm, ST changes, T wave inversion	Normal sinus rhythm, normal intervals, early repolarization pattern
	Commercial	1	Normal sinus rhythm, normal intervals, early repolarization pattern	Normal sinus rhythm, ST changes, T wave inversion	Normal sinus rhythm, normal intervals, early repolarization pattern
	Summary		All 4 ECGS had similar interpretation	Both 12 and single lead ECGs had similar interpretation	All 4 ECGS had similar interpretation

Volunteer 10_KAUST_Single lead ECG system

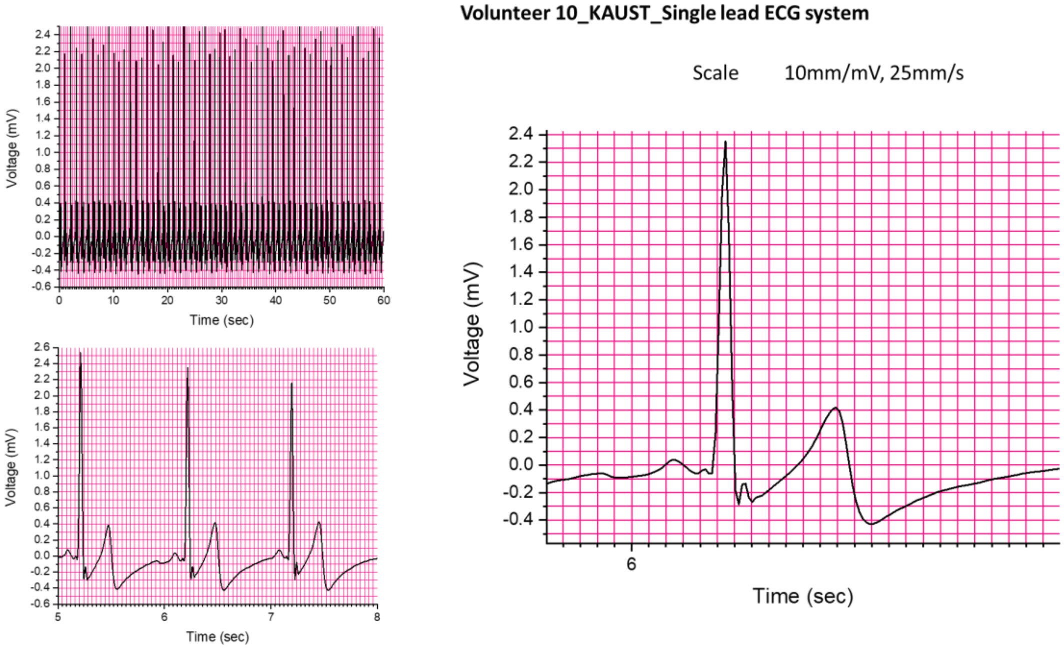

Volunteer 10_Commercial_Single lead ECG system

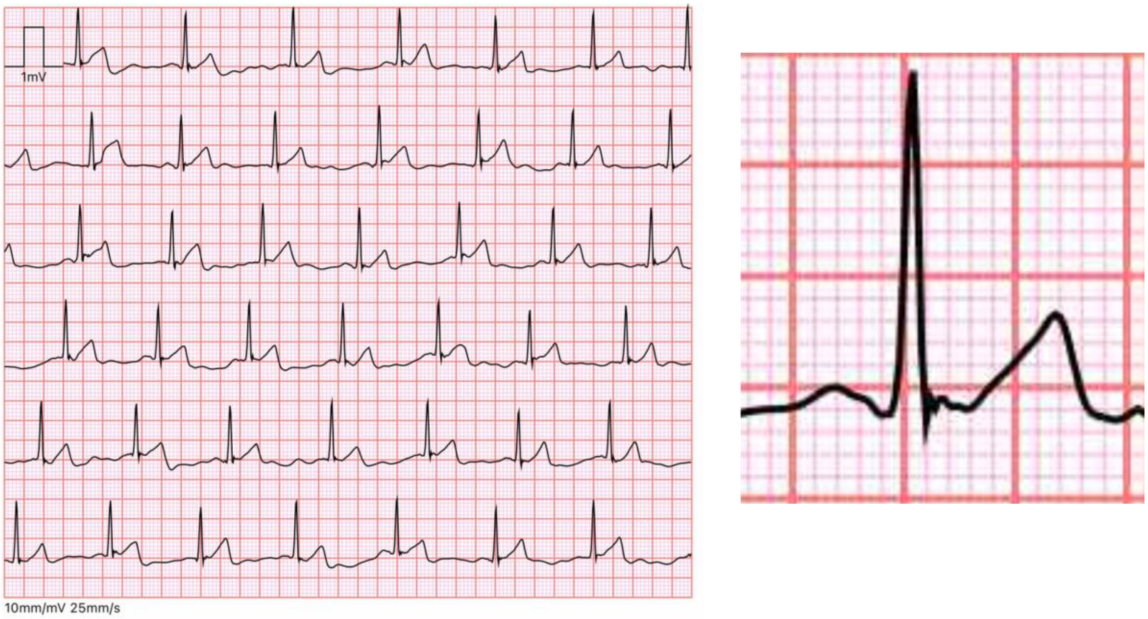

Figure S22a: Volunteer 10, KAUST single lead, and commercial single-lead ECG.

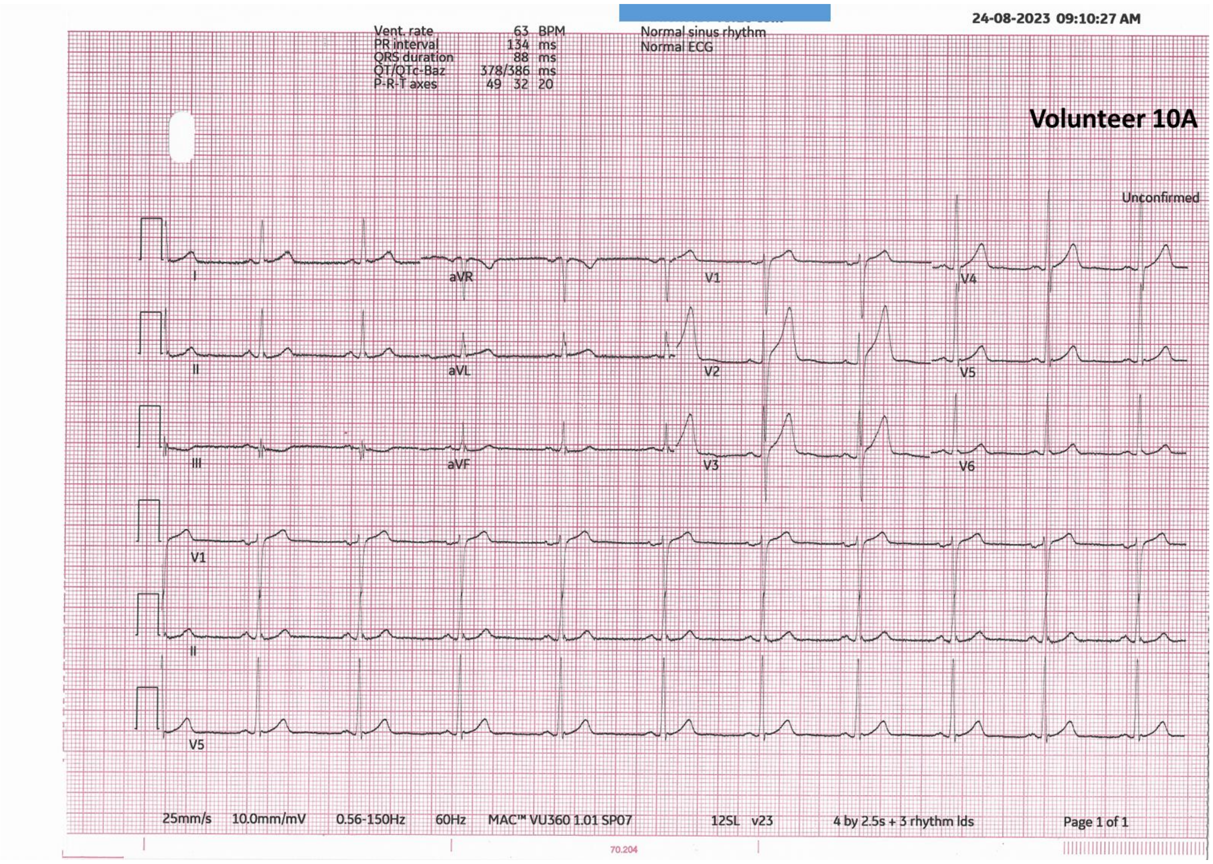

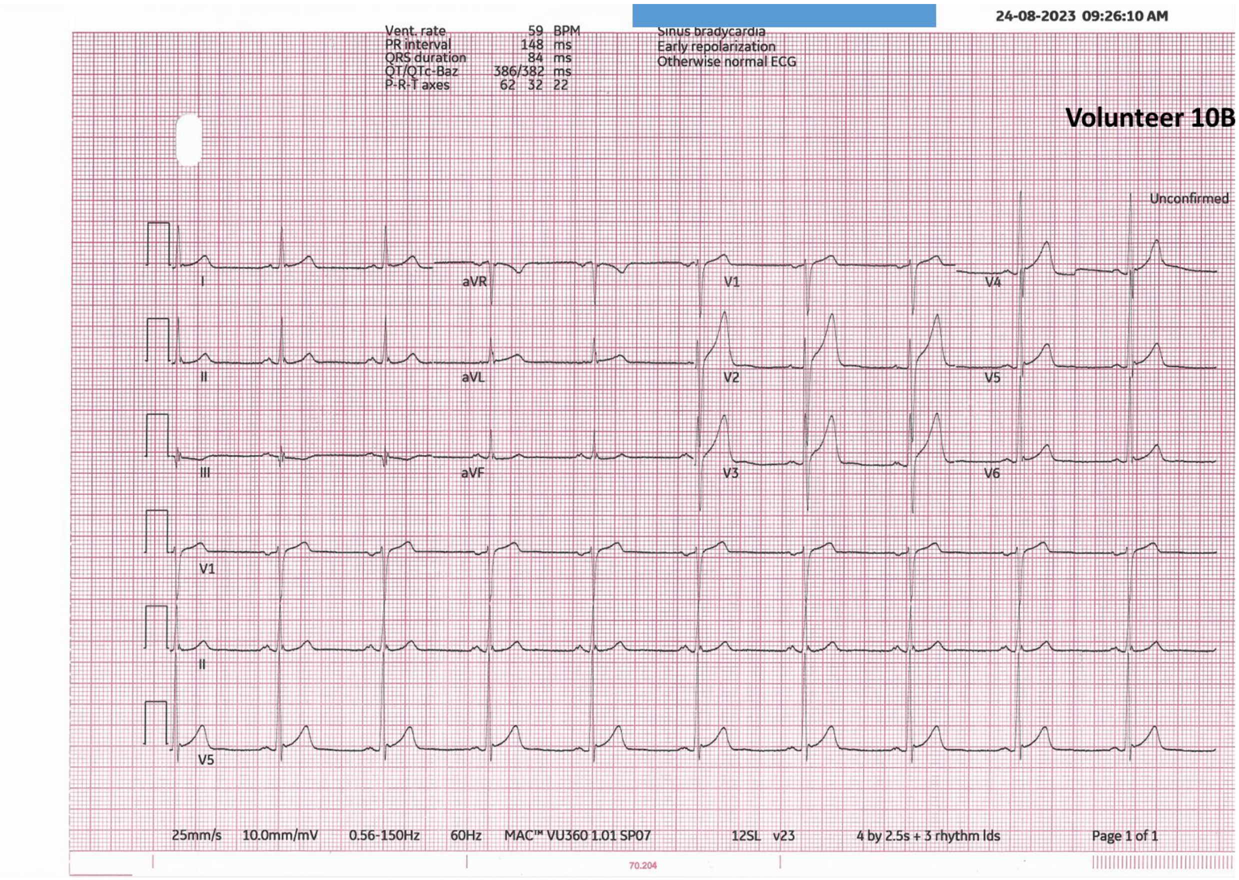

Figure S22b: Volunteer 10: Blinded review of 12 lead ECG using commercial ECG electrodes and gentle-to-skin screen printed ECG electrodes. The sample identification was hidden from the reviewers using a blue color square.

Volunteer No	ECG Type	Leads	Reviewer 1 (Cardiology Consultant)	Reviewer 2 (Cardiology Consultant)	Reviewer 3 (FM Consultant)
V11	A	12	Normal sinus rhythm, short PR interval, otherwise normal ECG	Sinus arrhythmia<br>Normal intervals and segments<br>U wave is noted (normal variant)	Sinus arrythmia with Short PR interval, normal segments
	B	12	Normal sinus rhythm, short PR interval, otherwise normal ECG	Sinus arrhythmia<br>Normal intervals and segments<br>U wave is noted (normal variant)	Sinus arrythmia with Short PR interval, normal segments
	KAUST	1	Normal sinus rhythm, short PR interval, otherwise normal ECG	Sinus arrhythmia<br>Normal intervals and segments	Sinus arrythmia with Short PR interval, normal segments
	Commercial	1	Normal sinus rhythm, short PR interval, otherwise normal ECG	Sinus arrhythmia<br>Normal intervals and segments	Sinus arrythmia with Short PR interval, normal segments
	Summary		All 4 ECGS had similar interpretation	Both 12 and single lead ECGs had similar interpretation	All 4 ECGS had similar interpretation

Volunteer 11_KAUST_Single lead ECG system

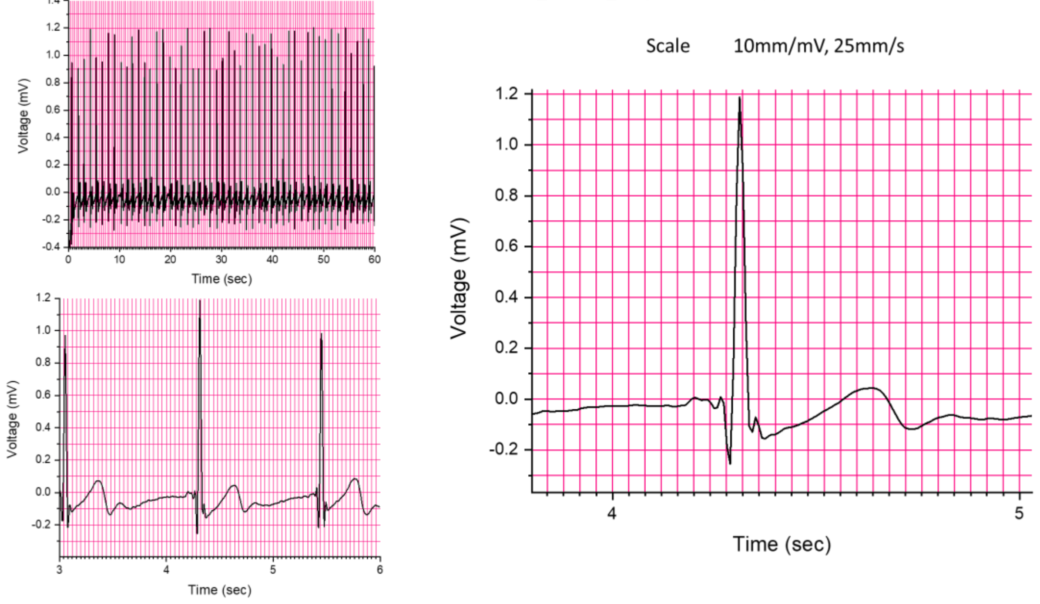

Volunteer 11_Commercial_Single lead ECG system

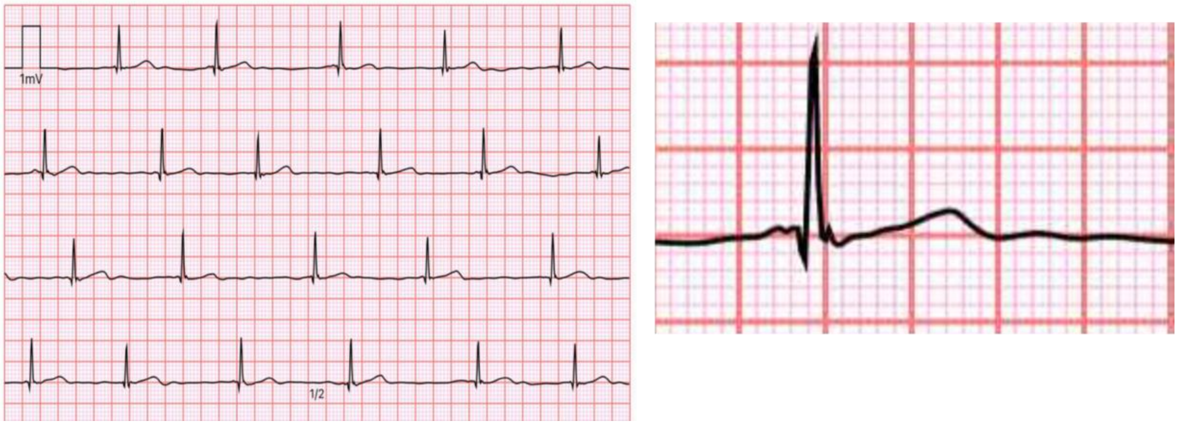

Figure S23a: Volunteer 11, KAUST single lead and commercial single-lead ECG.

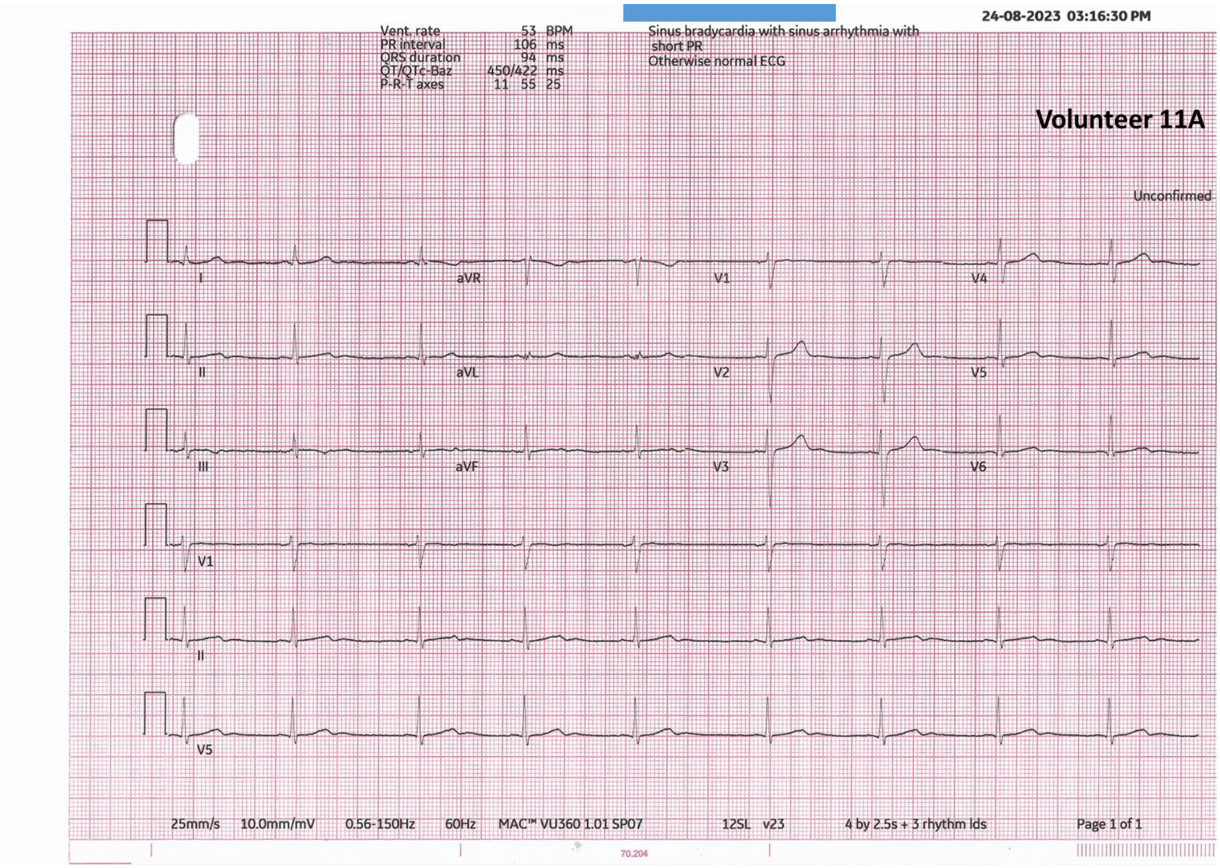

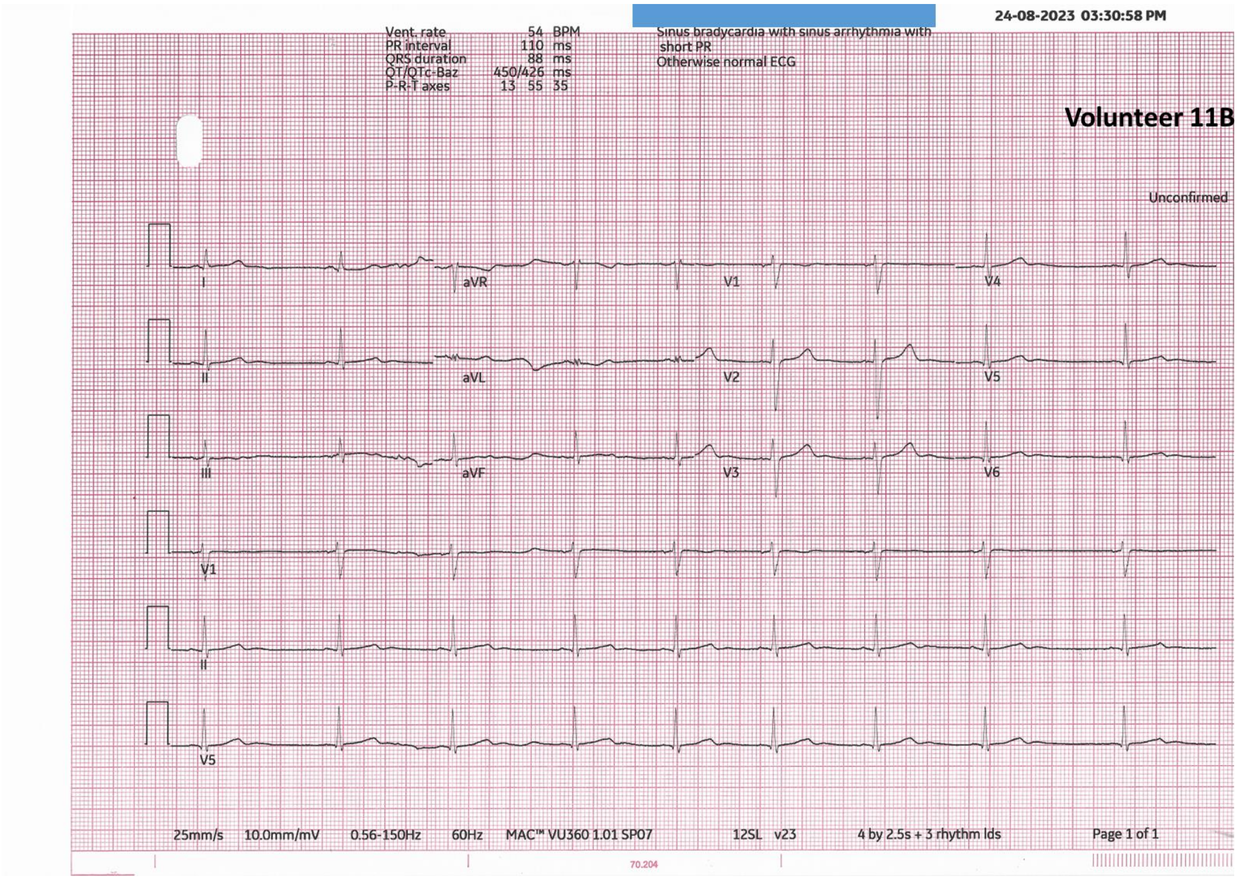

Figure S23b: Volunteer 11: Blinded review of 12 lead ECG using commercial ECG electrodes and gentle-to-skin screen printed ECG electrodes. The sample identification was hidden from the reviewers using a blue color square.

Volunteer No	ECG Type	Leads	Reviewer 1 (Cardiology Consultant)	Reviewer 2 (Cardiology Consultant)	Reviewer 3 (FM Consultant)
**V12**	A	12	Normal sinus rhythm, normal intervals	Normal sinus rhythm, normal intervals, normal ST segments	Normal sinus rhythm, normal intervals, normal ST segments
	B	12	Normal sinus rhythm, normal intervals	Normal sinus rhythm, normal intervals, normal ST segments	Normal sinus rhythm, normal intervals, normal ST segments
	KAUST	1	Normal sinus rhythm, normal intervals	NSR, T wave inversion, Artifact is noted	Normal sinus rhythm, normal intervals, normal ST segments
	Commercial	1	Normal sinus rhythm, normal intervals	NSR, T wave inversion, Artifact is noted	Normal sinus rhythm, normal intervals, normal ST segments
	Summary		All 4 ECGS had similar interpretation	Both 12 and single lead ECGs had similar interpretation	All 4 ECGS had similar interpretation

Volunteer 12_KAUST_Single lead ECG system

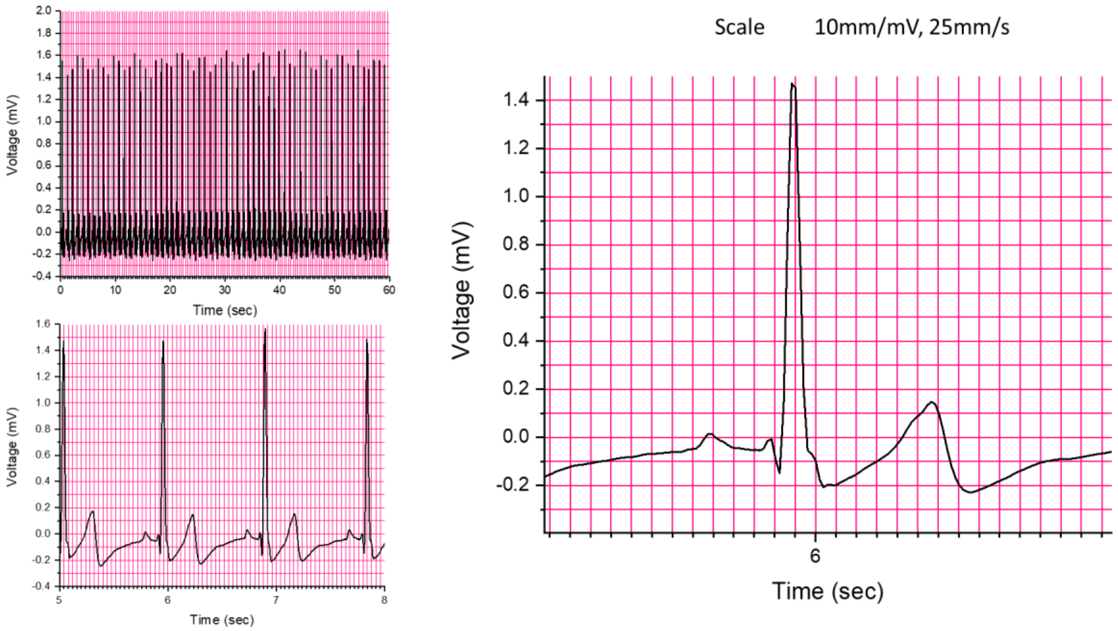

Volunteer 12_Commercial_Single lead ECG system

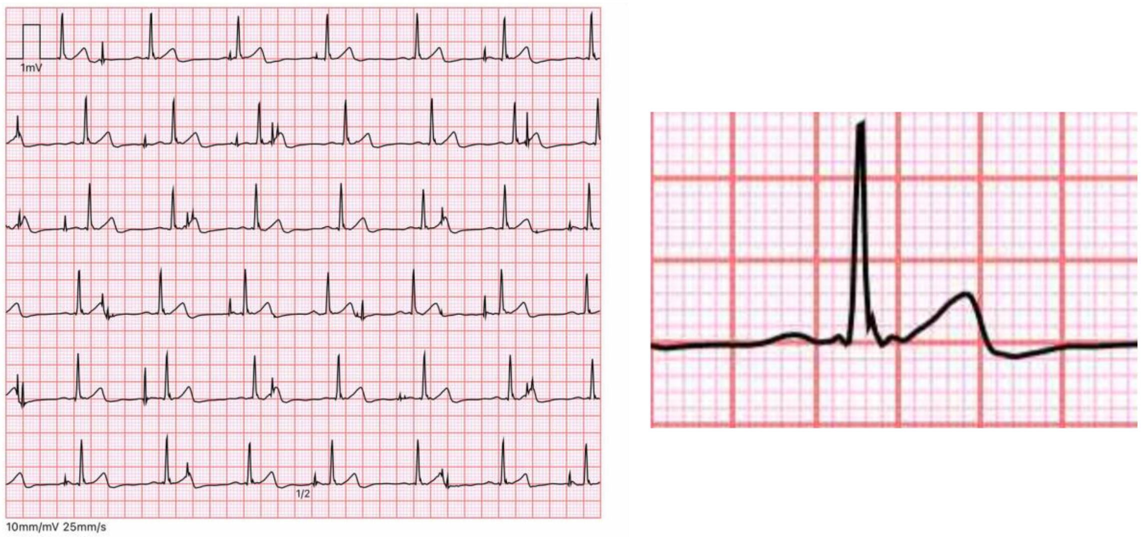

Figure S24a: Volunteer 12, KAUST single lead, and commercial single-lead ECG.

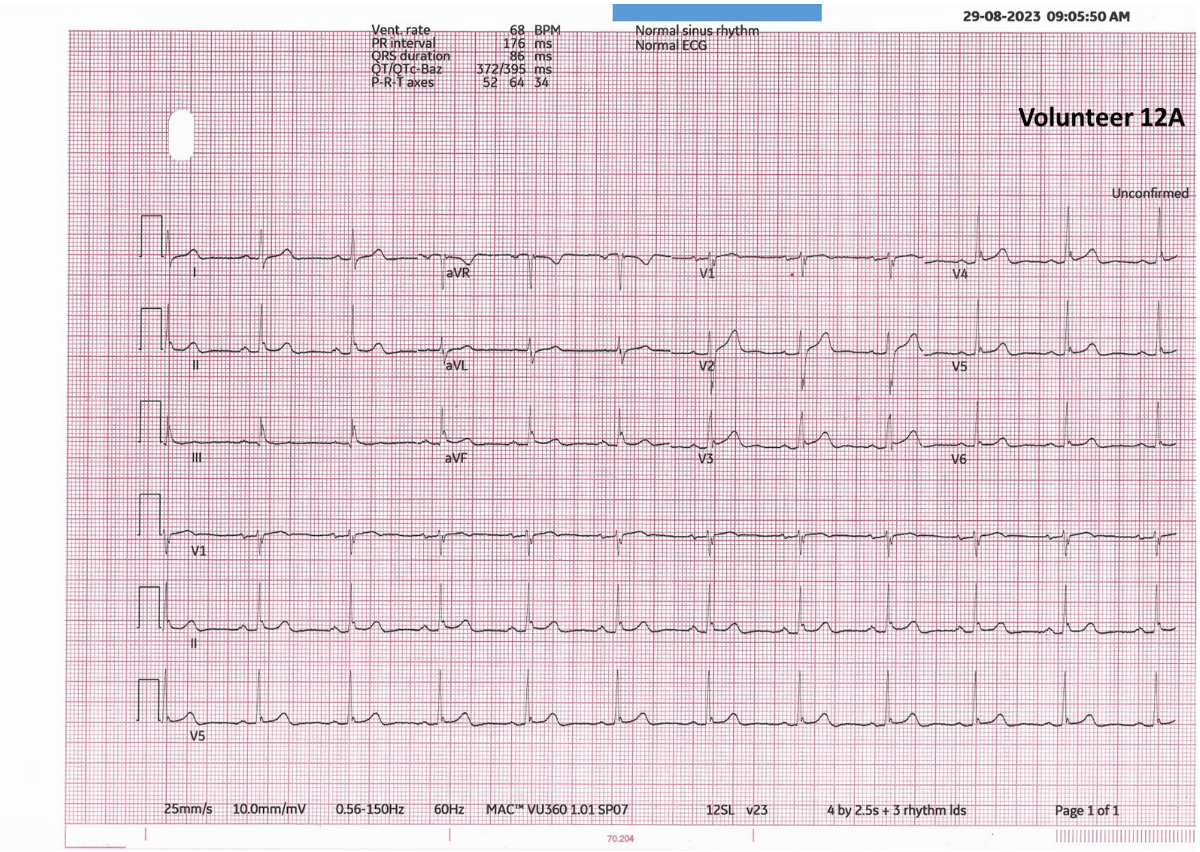

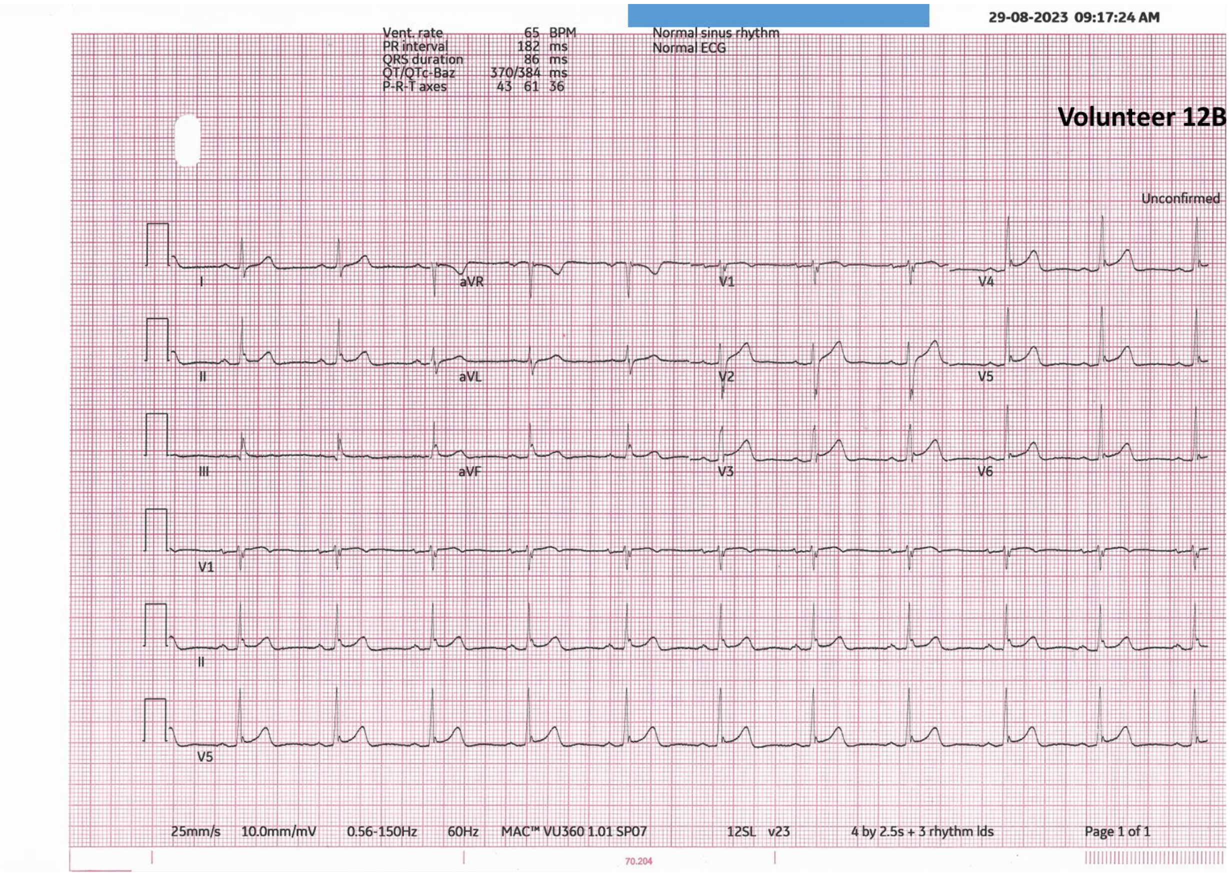

Figure S24b: Volunteer 12: Blinded review of 12 lead ECG using commercial ECG electrodes and gentle-to-skin screen printed ECG electrodes. The sample identification was hidden from the reviewers using a blue color square.

Volunteer No	ECG Type	Leads	Reviewer 1 (Cardiology Consultant)	Reviewer 2 (Cardiology Consultant)	Reviewer 3 (FM Consultant)
V13	A	12	Normal sinus ryhtm, nonspecific interventricular conduction delay/ may be normal variant	NSR, ST depression inferior leads with T wave inversion. RSR patgern in V1, Normal intervals	Normal sinus ryhtm, nonspecific interventricular conduction delay, T inversion in III and aVf,
	B	12	Normal sinus ryhtm, nonspecific interventricular conduction delay/ may be normal variant	NSR, ST depression inferior leads with T wave inversion. RSR patgern in V1, Normal intervals	Normal sinus ryhtm, nonspecific interventricular conduction delay, T inversion in III and aVf,
	KAUST	1	Normal sinus rhythm, normal intervals, normal ST segments	NSR, Fractionated QRS, Normal ST segment, Normal T wave	Normal sinus rhythm, normal intervals, normal ST segments
	Commercial	1	Normal sinus rhythm, normal intervals, normal ST segments	NSR, Fractionated QRS, Normal ST segment, Normal T wave	Normal sinus rhythm, normal intervals, normal ST segments
	Summary		All 4 ECGS had similar interpretation clinically, both single channel recordings were nearly identical	Both 12 and single lead ECGs had similar interpretation	Both 12 and single lead ECGs had similar interpretation

Volunteer 13_KAUST_Single lead ECG system

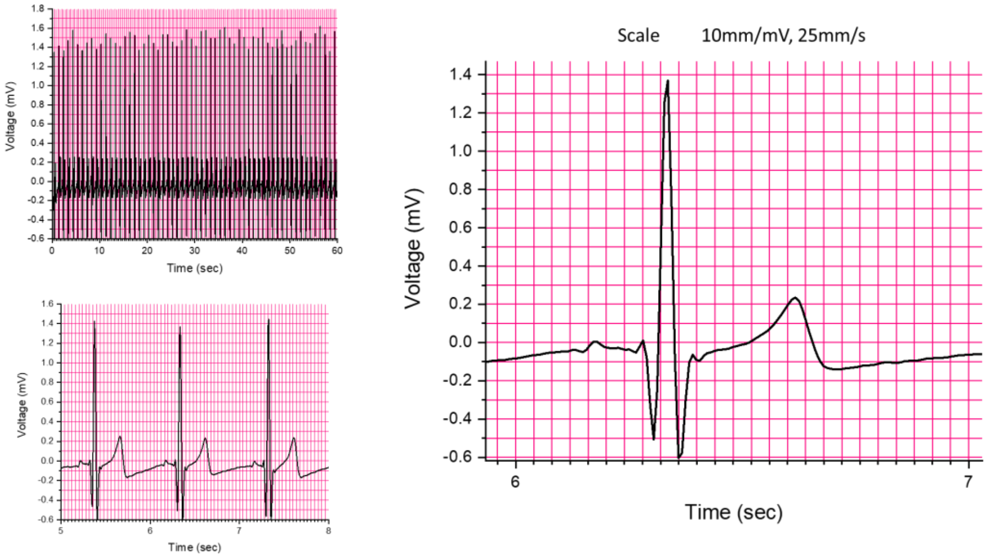

Volunteer 13_Commercial_Single lead ECG system

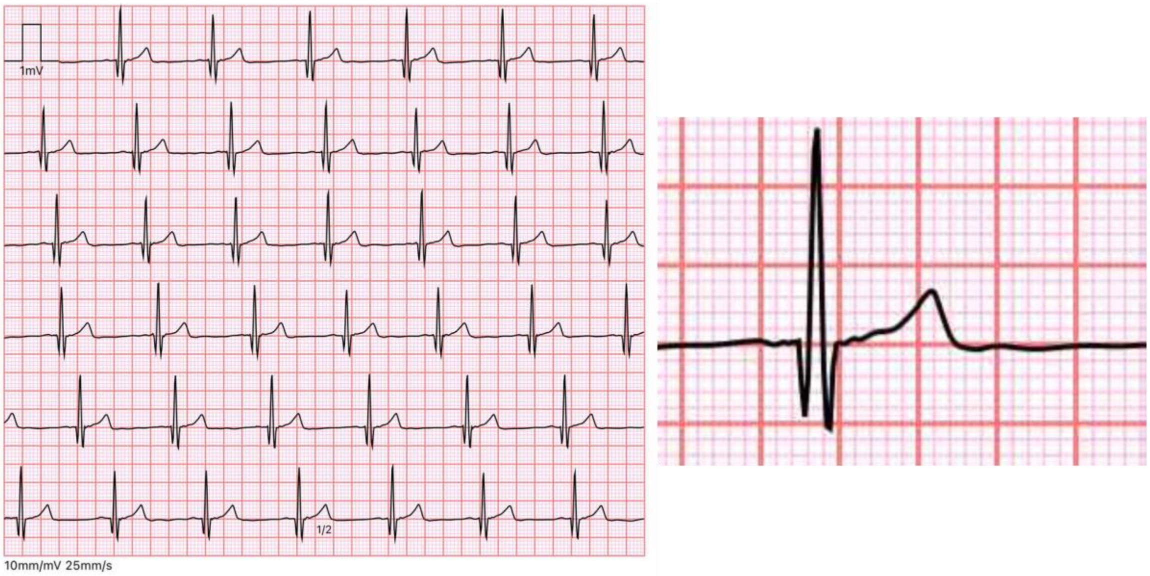

Figure S25a: Volunteer 13, KAUST single lead, and commercial single-lead ECG.

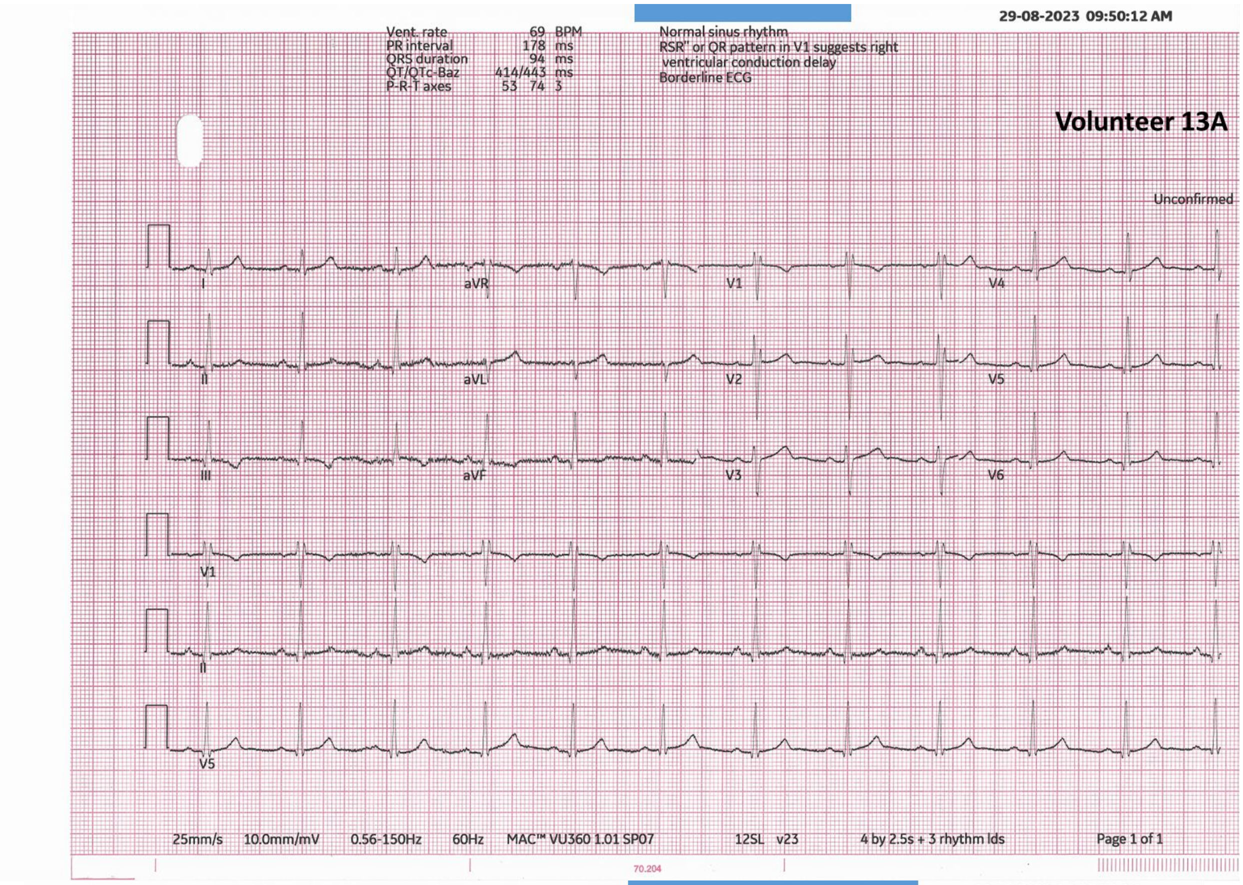

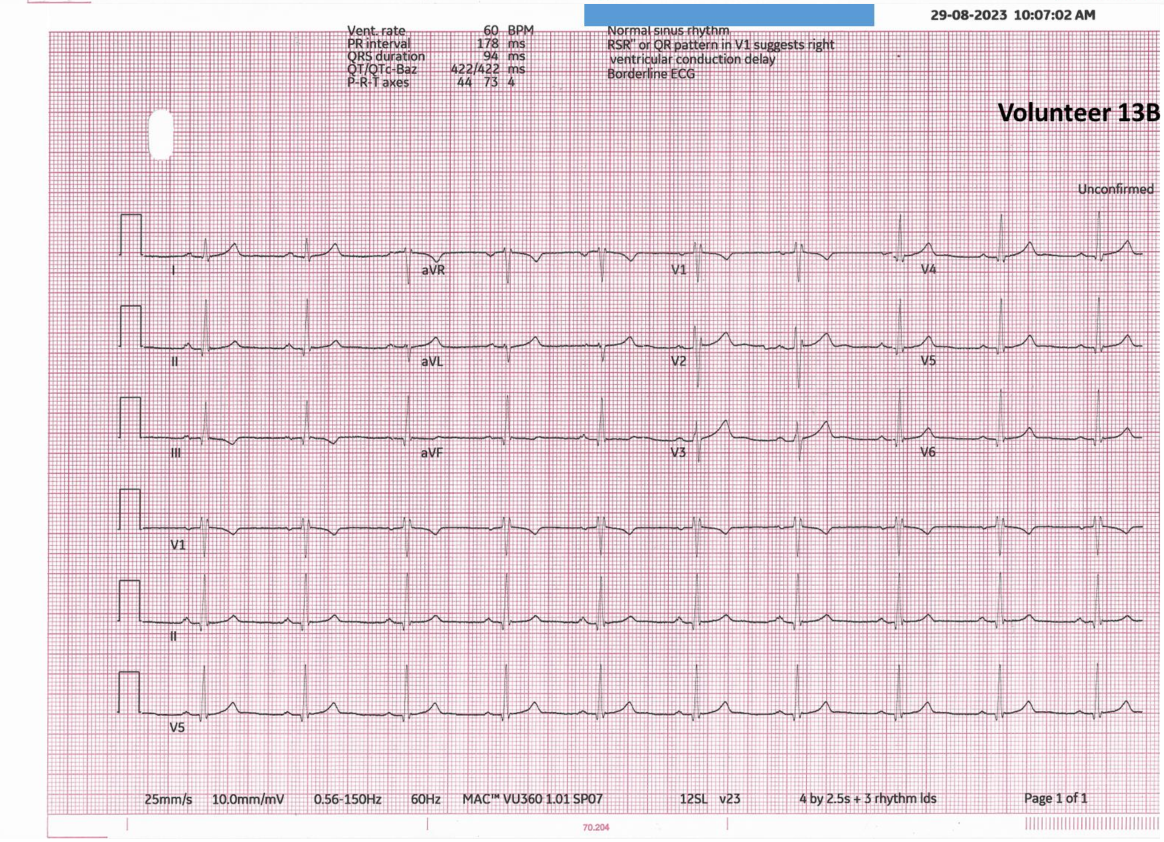

Figure S25b: Volunteer 13: Blinded review of 12 lead ECG using commercial ECG electrodes and gentle-to-skin screen printed ECG electrodes. The sample identification was hidden from the reviewers using a blue color square.

Volunteer No	ECG Type	Leads	Reviewer 1 (Cardiology Consultant)	Reviewer 2 (Cardiology Consultant)	Reviewer 3 (FM Consultant)
V14	A	12	Normal sinus rhythm, normal intervals, normal ST segments	Normal sinus rhythm, normal intervals, normal ST segments	Normal sinus rhythm, normal intervals, normal ST segments
	B	12	Normal sinus rhythm, normal intervals, normal ST segments	Normal sinus rhythm, normal intervals, normal ST segments	Normal sinus rhythm, normal intervals, normal ST segments
	KAUST	1	Normal sinus rhythm, normal intervals, normal ST segments	Normal sinus rhythm, normal intervals, normal ST segments	Normal sinus rhythm, normal intervals, normal ST segments
	Commercial	1	Normal sinus rhythm, normal intervals, normal ST segments	Normal sinus rhythm, normal intervals, normal ST segments	Normal sinus rhythm, normal intervals, normal ST segments
	Summary		All 4 ECGS had similar interpretation	Both 12 and single lead ECGs had similar interpretation	Both 12 and single lead ECGs had similar interpretation

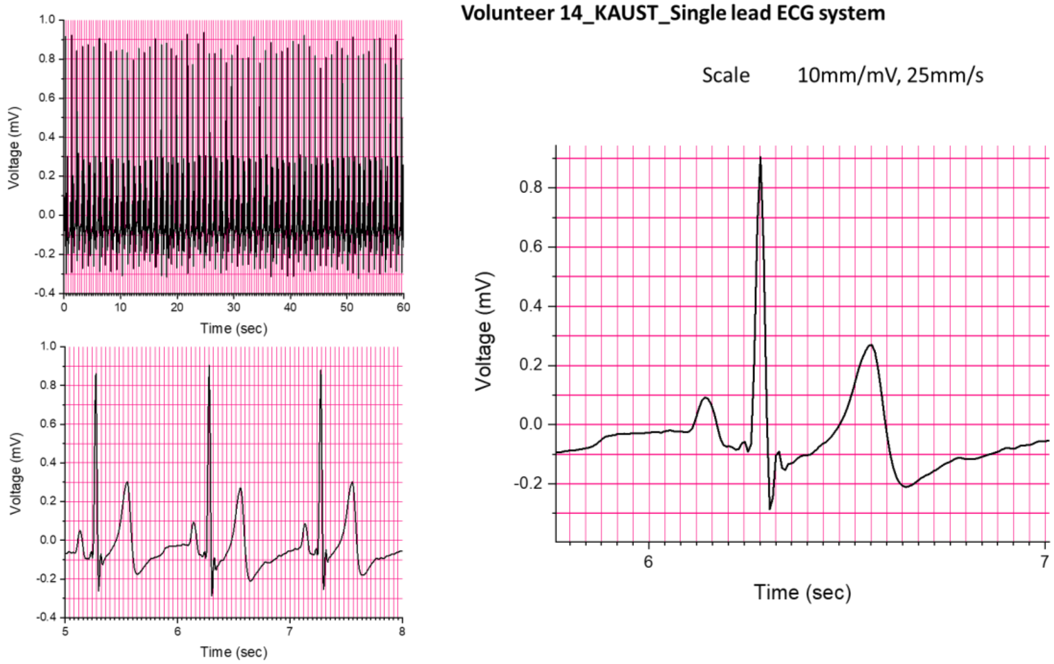

Volunteer 14_Commercial_Single lead ECG system

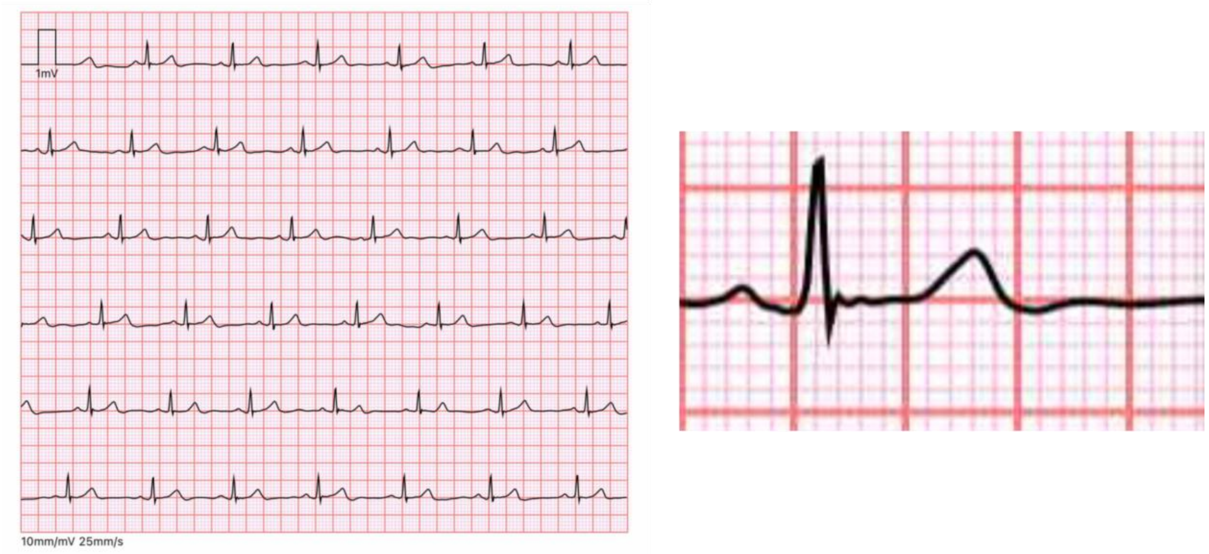

Figure S26a: Volunteer 14, KAUST single lead and commercial single-lead ECG.

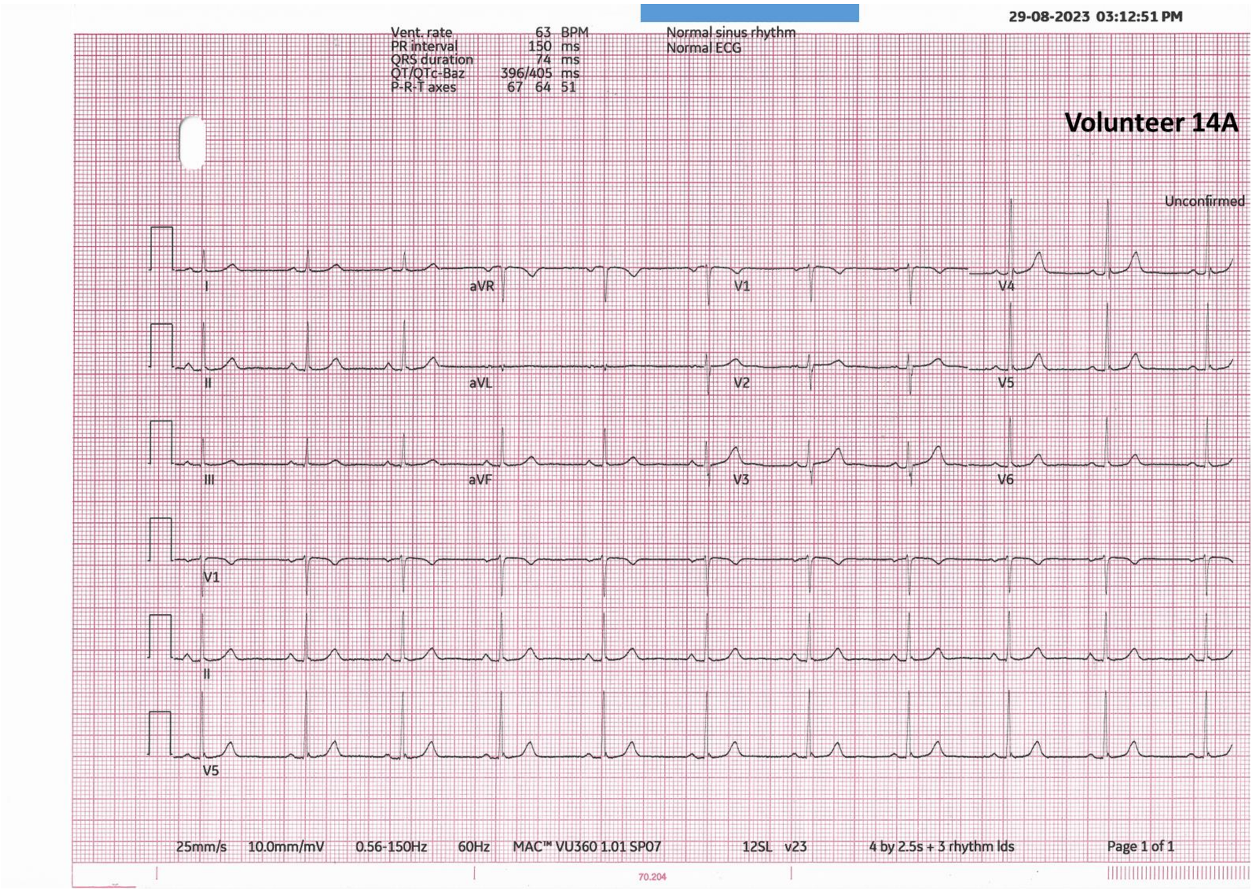

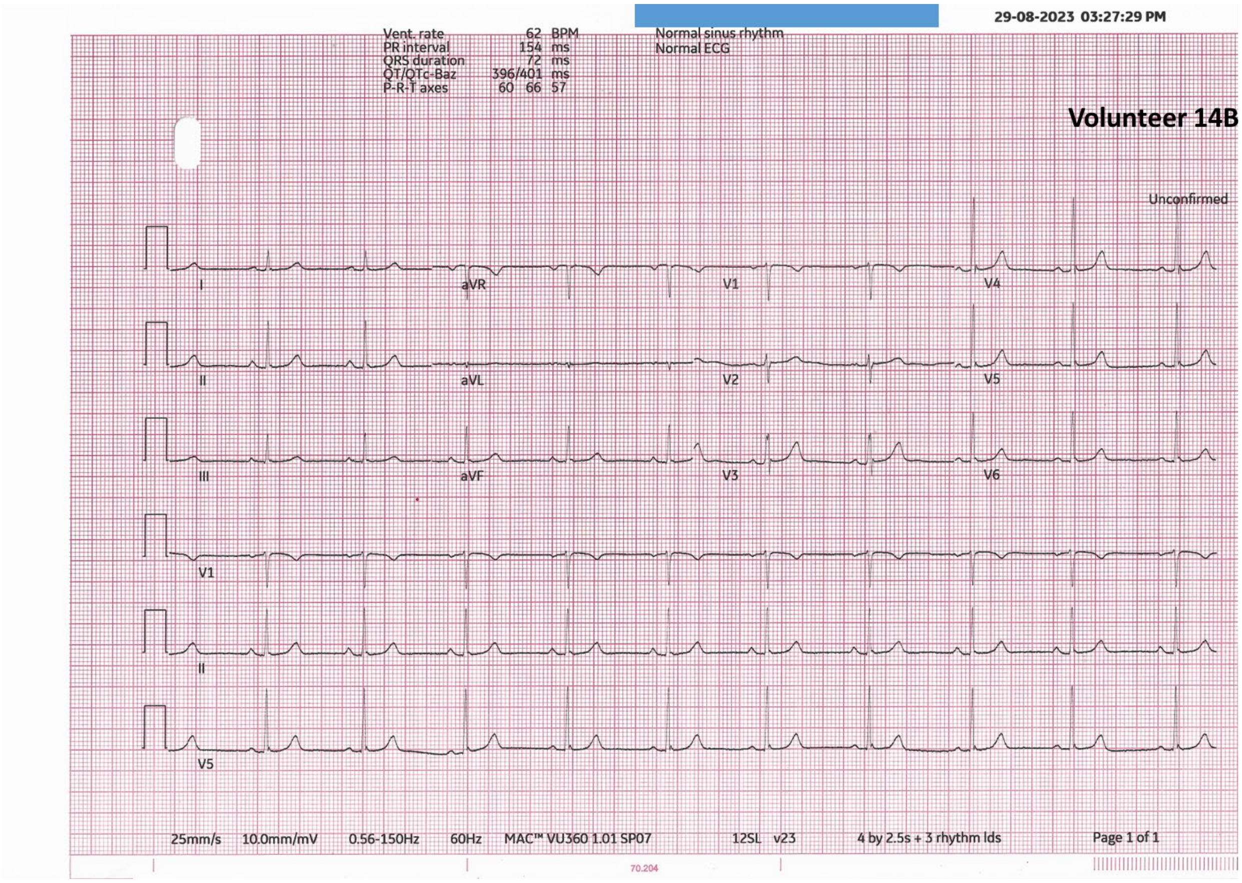

Figure S26b: Volunteer 14: Blinded review of 12 lead ECG using commercial ECG electrodes and gentle-to-skin screen printed ECG electrodes. The sample identification was hidden from the reviewers using a blue color square.

Volunteer No	ECG Type	Leads	Reviewer 1 (Cardiology Consultant)	Reviewer 2 (Cardiology Consultant)	Reviewer 3 (FM Consultant)
V15	A	12	Normal sinus rhythm, normal intervals, normal ST segments	Normal sinus rhythm, normal intervals, normal ST segments	Normal sinus rhythm, normal intervals, normal ST segments
	B	12	Normal sinus rhythm, normal intervals, normal ST segments	Normal sinus rhythm, normal intervals, normal ST segments	Normal sinus rhythm, normal intervals, normal ST segments
	KAUST	1	Normal sinus rhythm, normal intervals, normal ST segments	Normal sinus rhythm, normal intervals, normal ST segments	Normal sinus rhythm, normal intervals, normal ST segments
	Commercial	1	Normal sinus rhythm, normal intervals, normal ST segments	Normal sinus rhythm, normal intervals, normal ST segments	Normal sinus rhythm, normal intervals, normal ST segments
	Summary		All 4 ECGS had similar interpretation	All 4 ECGS had similar interpretation	All 4 ECGS had similar interpretation

Volunteer 15_KAUST_Single lead ECG system

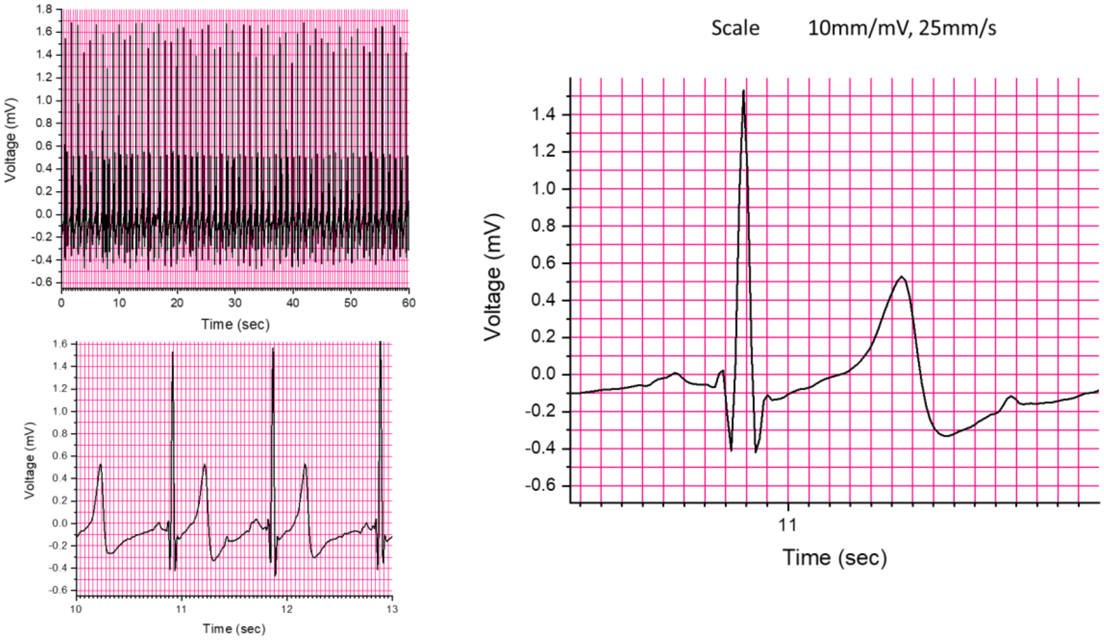

Volunteer 15_Commercial_Single lead ECG system

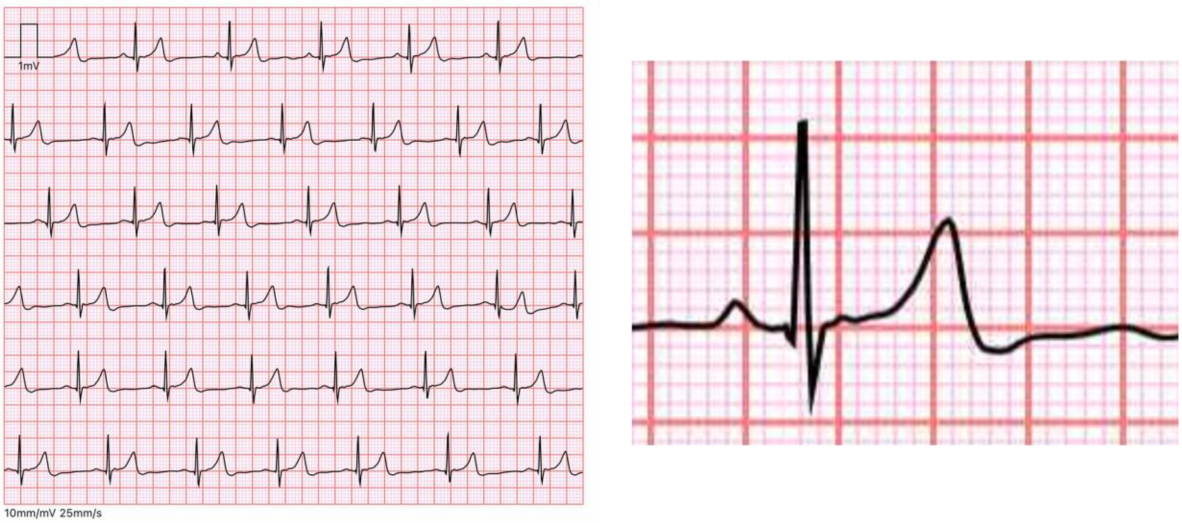

Figure S27a: Volunteer 15, KAUST single lead and commercial single-lead ECG.

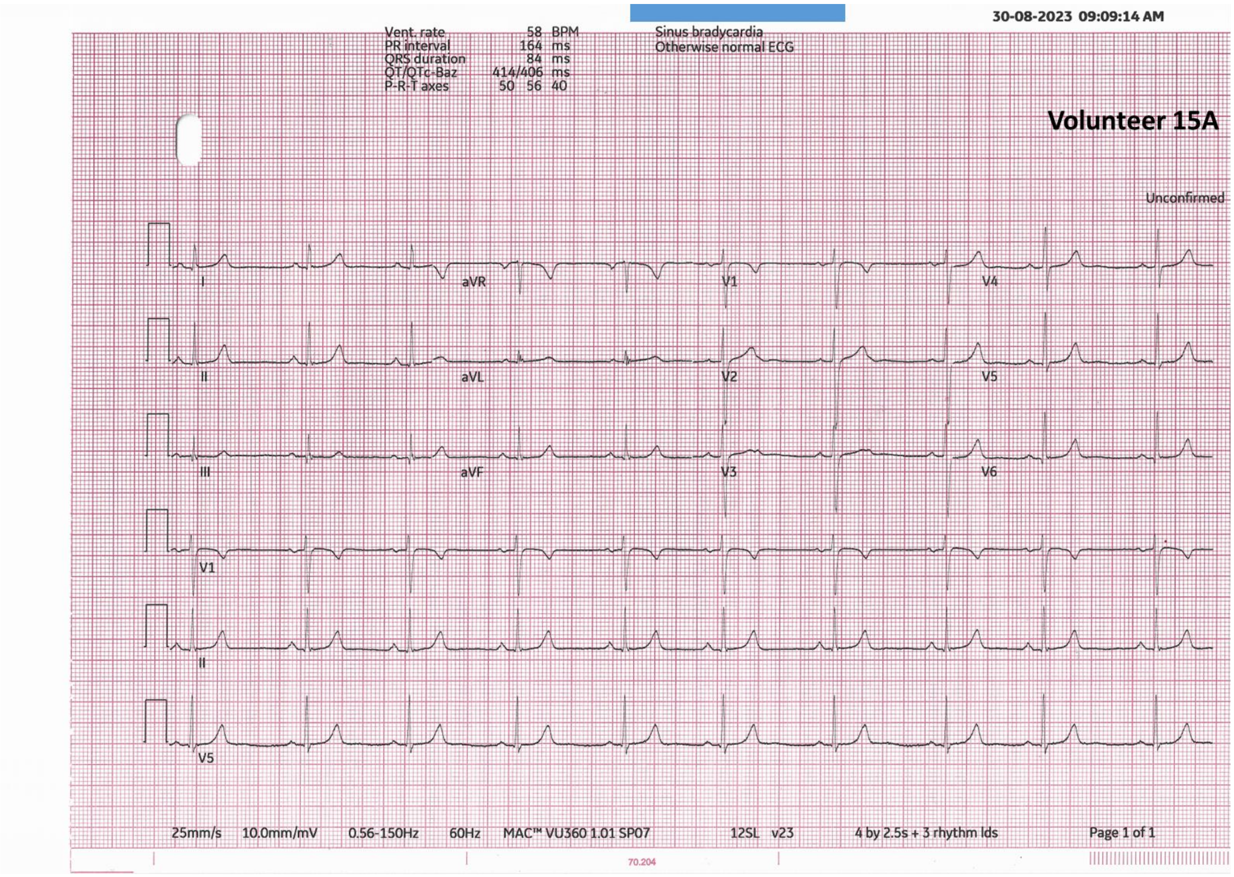

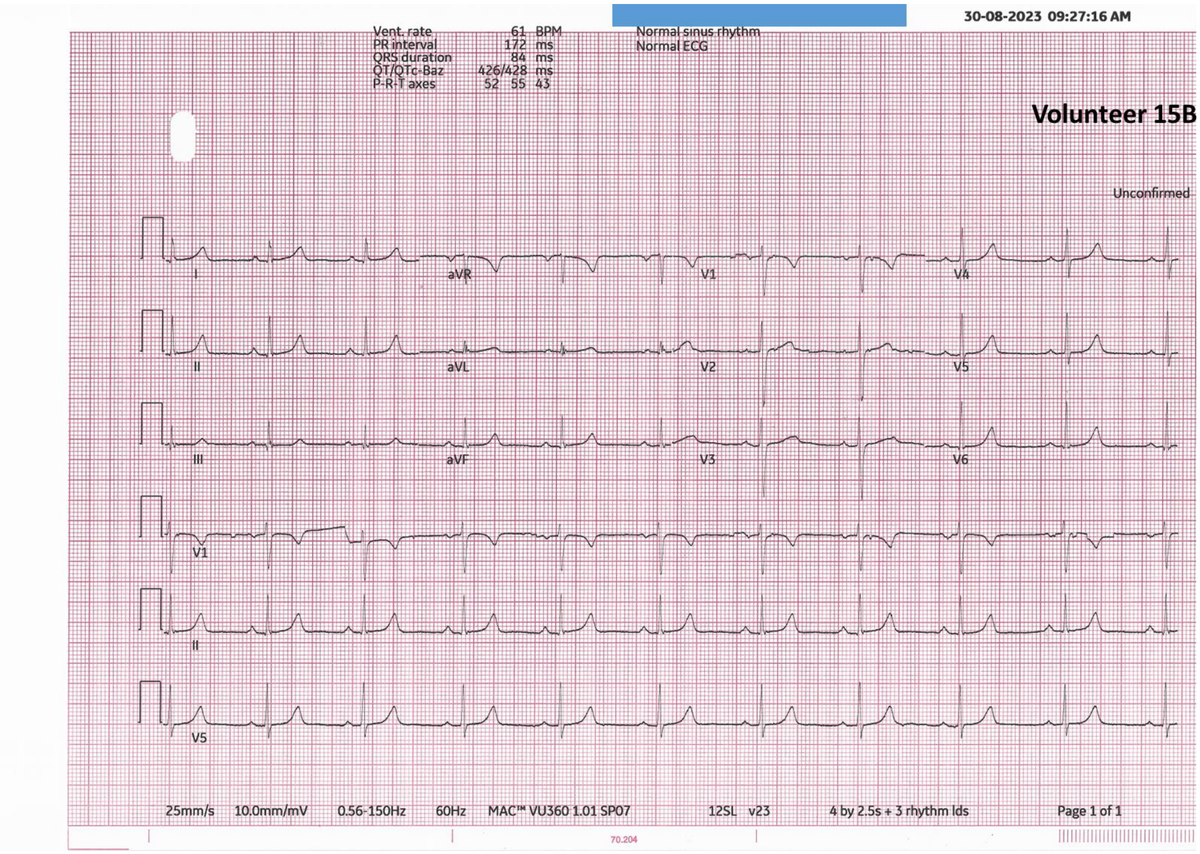

Figure S27b: Volunteer 15: Blinded review of 12 lead ECG using commercial ECG electrodes and gentle-to-skin screen printed ECG electrodes. The sample identification was hidden from the reviewers using a blue color square.

Volunteer No	ECG Type	Leads	Reviewer 1 (Cardiology Consultant)	Reviewer 2 (Cardiology Consultant)	Reviewer 3 (FM Consultant)
V16	A	12	Normal sinus rhythm, normal intervals, normal ST segments	Normal sinus rhythm, normal intervals, normal ST segments	Normal sinus rhythm, normal intervals, normal ST segments
	B	12	Normal sinus rhythm, normal intervals, normal ST segments	Normal sinus rhythm, normal intervals, normal ST segments	Normal sinus rhythm, normal intervals, normal ST segments
	KAUST	1	Normal sinus rhythm, normal intervals, early repolarization pattern	NSR, Normal intervals and segments, Few artifacts, Low amplitude	Normal sinus rhythm, normal intervals, early repolarization pattern
	Commercial	1	Normal sinus rhythm, normal intervals, early repolarization pattern	NSR, Normal intervals and segments, Few artifacts, Low amplitude	Normal sinus rhythm, normal intervals, early repolarization pattern
	Summary		Both 12 and single lead ECGs had similar interpretation	Both 12 and single lead ECGs had similar interpretation	Both 12 and single lead ECGs had similar interpretation

Volunteer 16_KAUST_Single lead ECG system

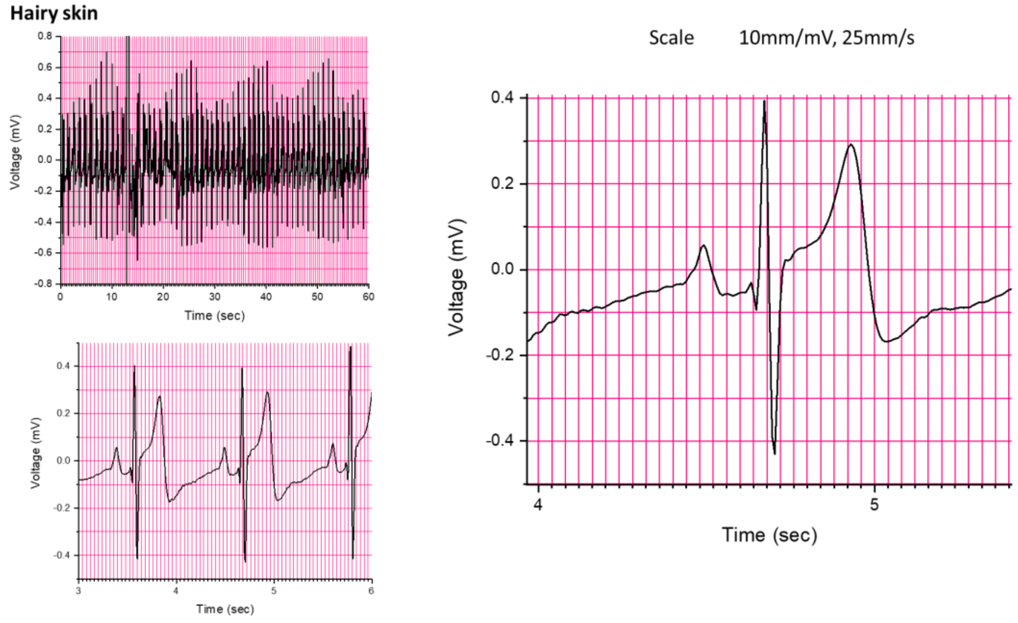

Volunteer 16_Commercial_Single lead ECG system

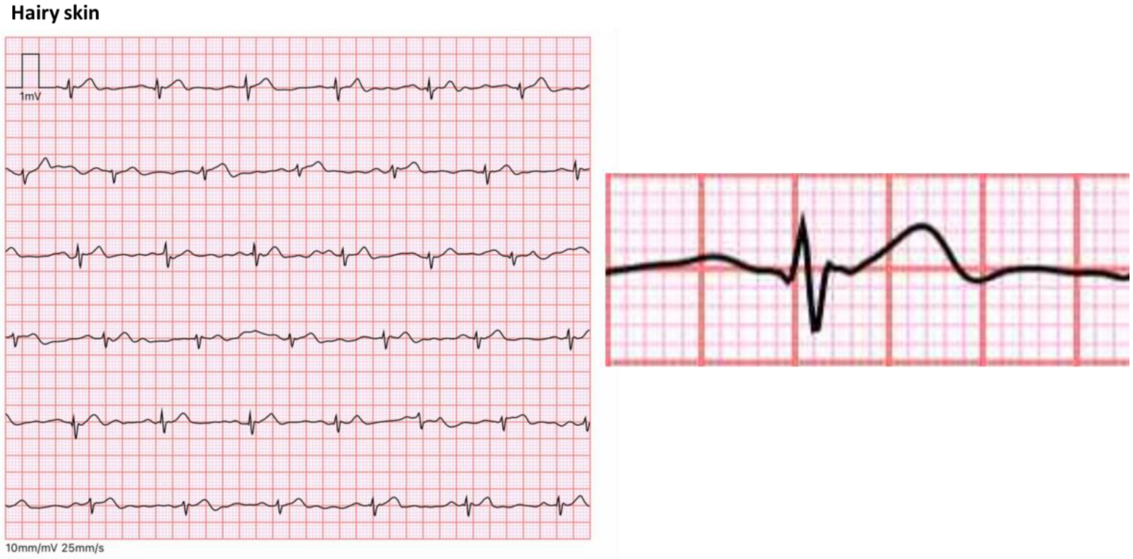

Figure S28a: Volunteer 16, KAUST single lead, and commercial single-lead ECG.

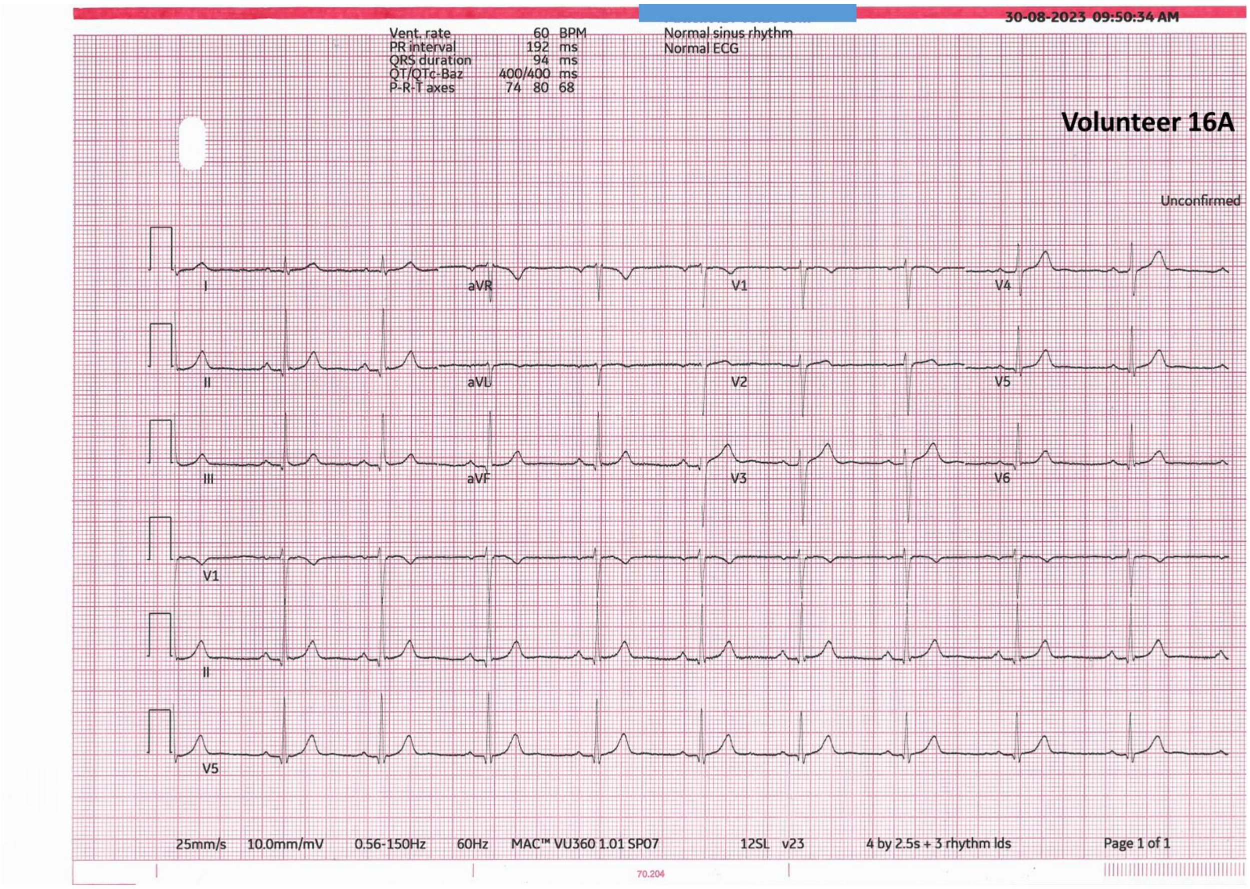

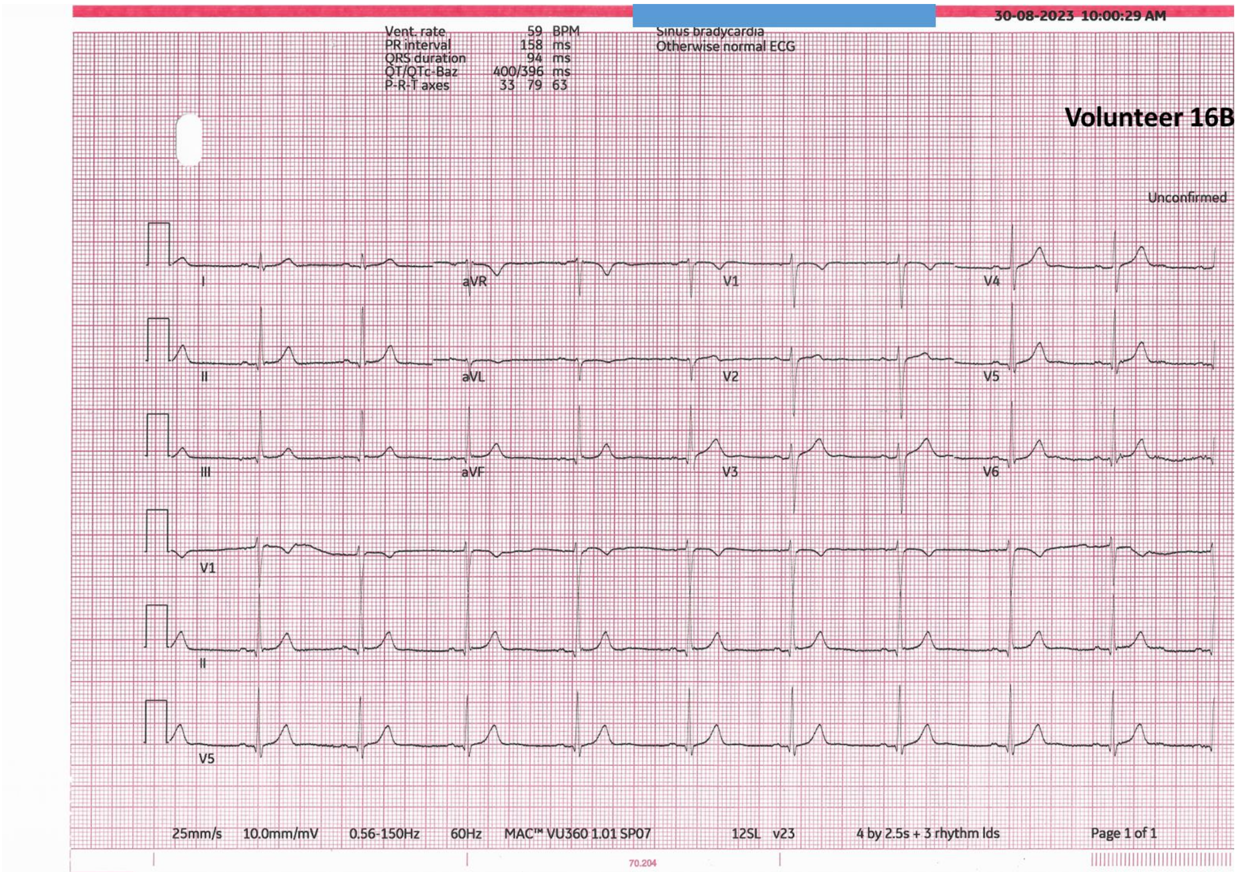

Figure S28b: Volunteer 16: Blinded review of 12 lead ECG using commercial ECG electrodes and gentle-to-skin screen printed ECG electrodes. The sample identification was hidden from the reviewers using a blue color square.

Volunteer No	ECG Type	Leads	Reviewer 1 (Cardiology Consultant)	Reviewer 2 (Cardiology Consultant)	Reviewer 3 (FM Consultant)
V17	A	12	Normal sinus rhythm, normal intervals, poor R wave progression	NSR, Right axis deviation, Anterior Q waves. Late R/S transition, Boderline low amplitude in the limb leads, Abnormal ECG	Normal sinus rhythm, normal intervals, anterior Q waves, poor R wave progression
	B	12	Normal sinus rhythm, normal intervals, poor R wave progression	NSR, Right axis deviation, Anterior Q waves. Late R/S transition, Boderline low amplitude in the limb leads, Abnormal ECG	Normal sinus rhythm, normal intervals, anterior Q waves, poor R wave progression
	KAUST	1	Normal sinus rhythm, normal intervals, normal ST segments	Normal sinus rhythm, normal intervals, normal ST segments	Normal sinus rhythm, normal intervals, normal ST segments
	Commercial	1	Normal sinus rhythm, normal intervals, normal ST segments	Normal sinus rhythm, normal intervals, normal ST segments	Normal sinus rhythm, normal intervals, normal ST segments
	Summary		R wave progression can only be detected on multilead recordings and was not detected on both single lead recordings, Both 12 and single lead ECGs had similar interpretation	Both 12 and single lead ECGs had similar interpretation	Anterior Q waves only seen in V1-V3 were not detected on both single lead recordings, Both 12 and single lead ECGs had similar interpretation

Volunteer 17_KAUST_Single lead ECG system

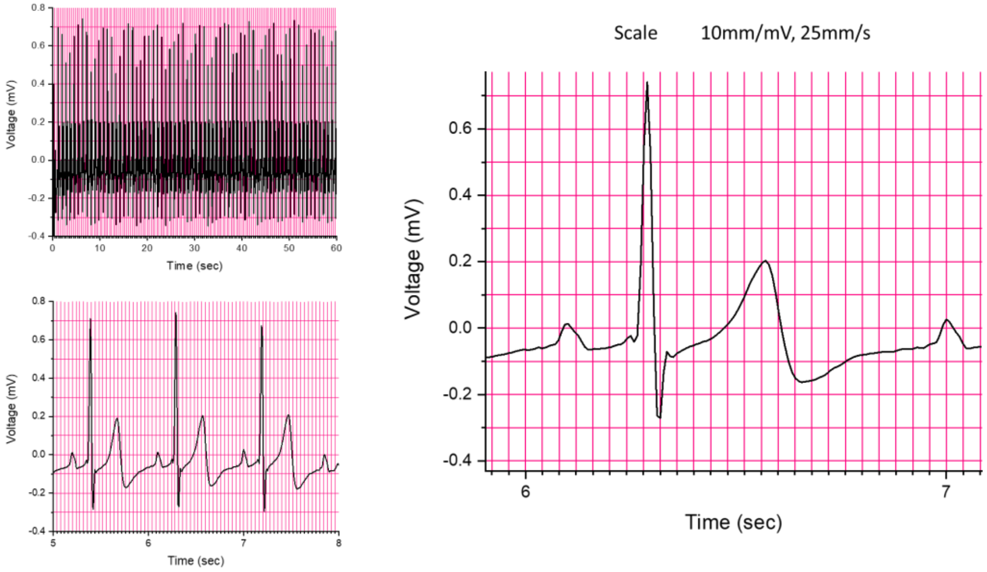

Volunteer 17_Commercial_Single lead ECG system

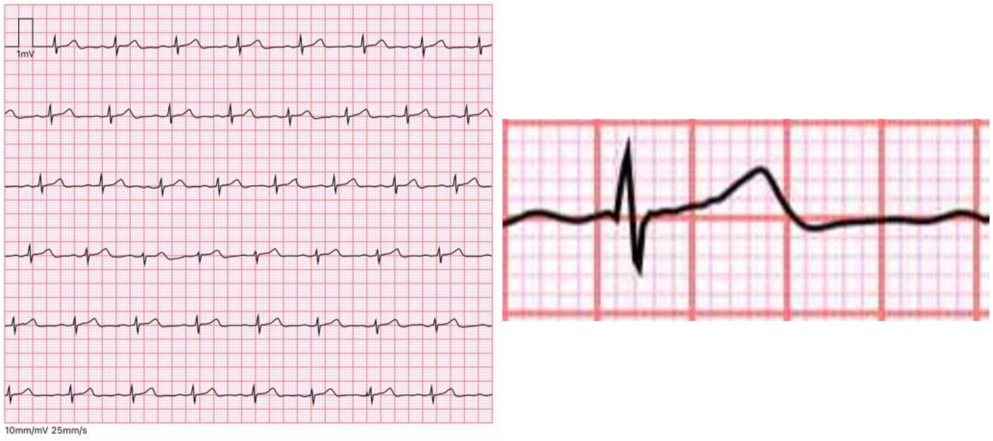

Figure S29a: Volunteer 17, KAUST single lead, and commercial single-lead ECG.

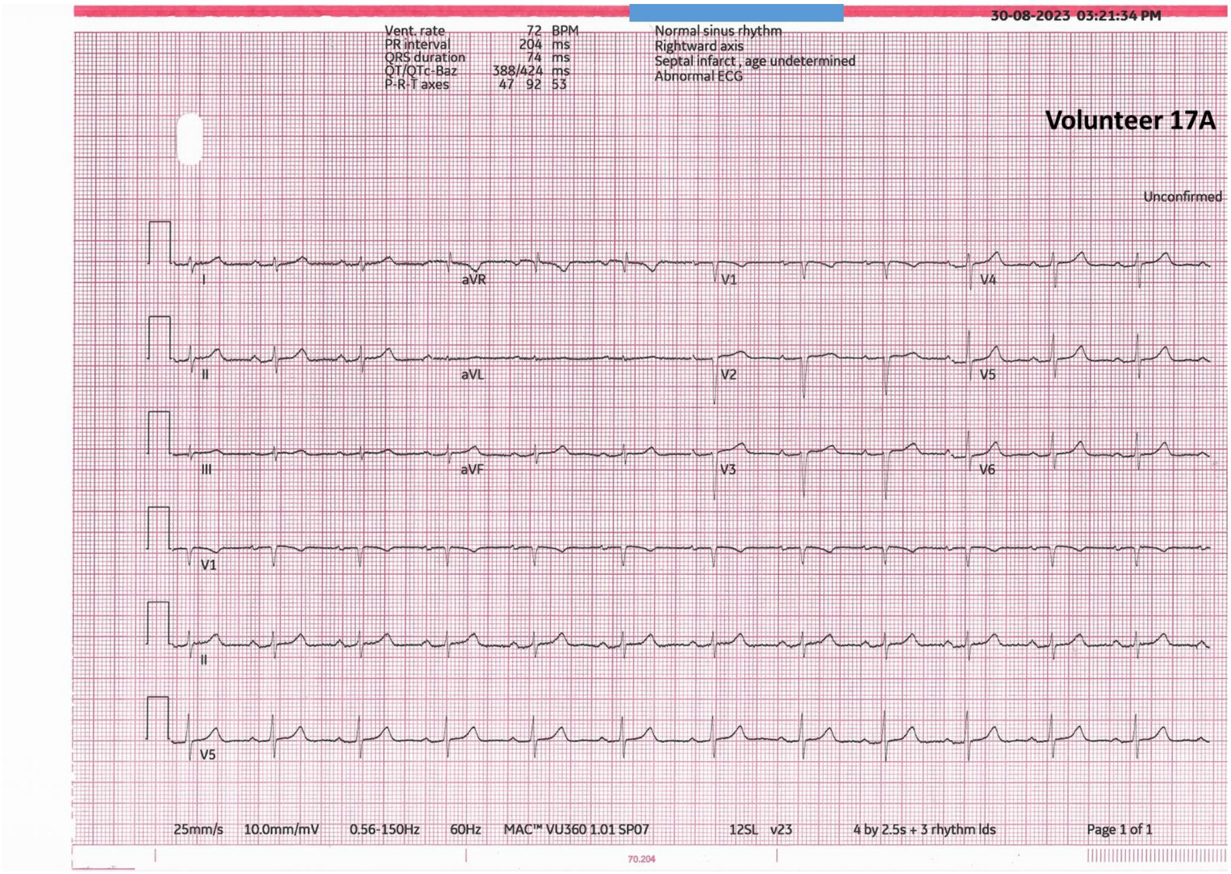

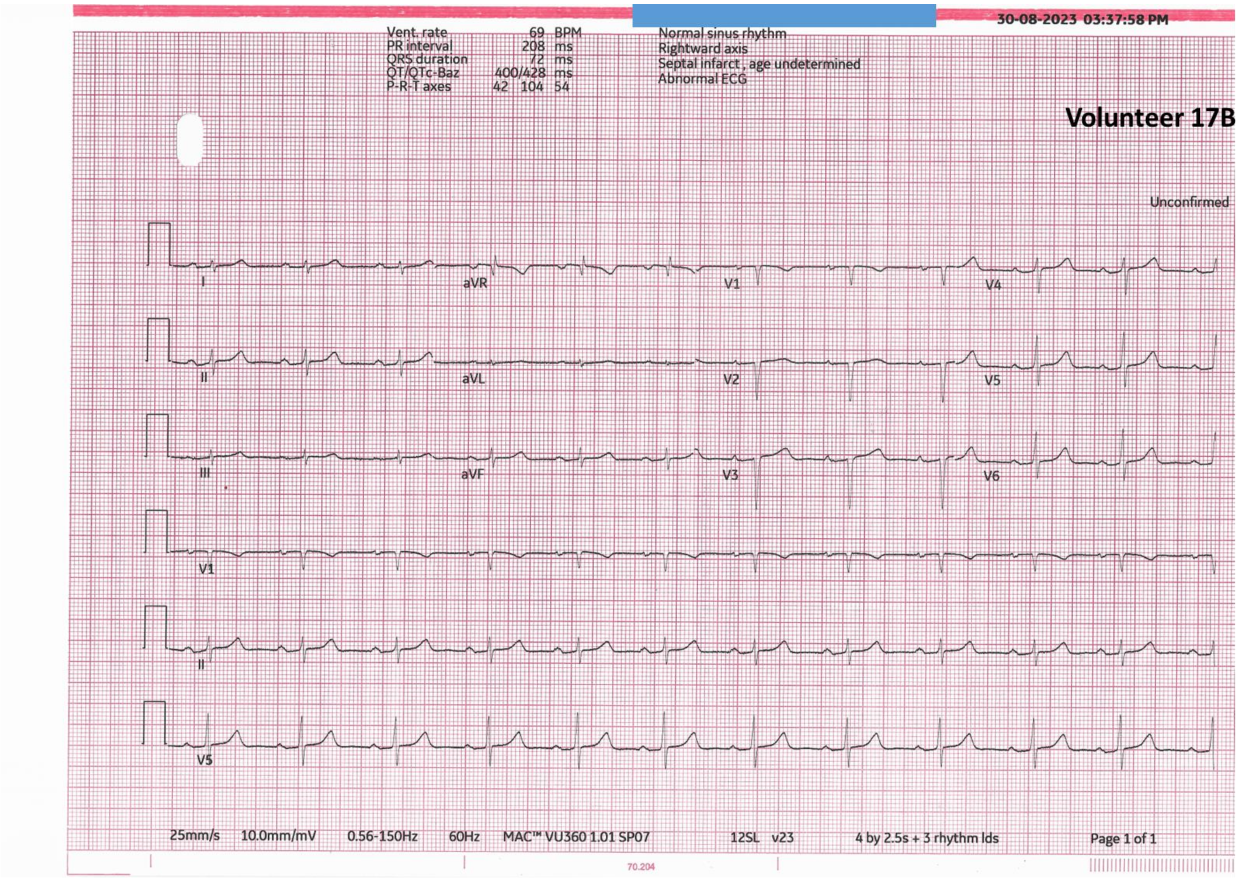

Figure S29b: Volunteer 17: Blinded review of 12 lead ECG using commercial ECG electrodes and gentle-to-skin screen printed ECG electrodes. The sample identification was hidden from the reviewers using a blue color square.

Volunteer No	ECG Type	Leads	Reviewer 1 (Cardiology Consultant)	Reviewer 2 (Cardiology Consultant)	Reviewer 3 (FM Consultant)
V18	A	12	Normal sinus rhythm, normal intervals, normal ST segments	NSR, Early R/S transition Normal intervals and segments	Normal sinus rhythm, normal intervals, normal ST segments
	B	12	Normal sinus rhythm, normal intervals, normal ST segments	NSR, Early R/S transition Normal intervals and segments	Normal sinus rhythm, normal intervals, normal ST segments
	KAUST	1	Normal sinus rhythm, normal intervals, normal ST segments	Normal sinus rhythm, normal intervals, normal ST segments	Normal sinus rhythm, normal intervals, normal ST segments
	Commercial	1	Normal sinus rhythm, normal intervals, normal ST segments	Normal sinus rhythm, normal intervals, normal ST segments	Normal sinus rhythm, normal intervals, normal ST segments
	Summary		All 4 ECGS had similar interpretation	Both 12 and single lead ECGs had similar interpretation	All 4 ECGS had similar interpretation

Volunteer 18_KAUST_Single lead ECG system

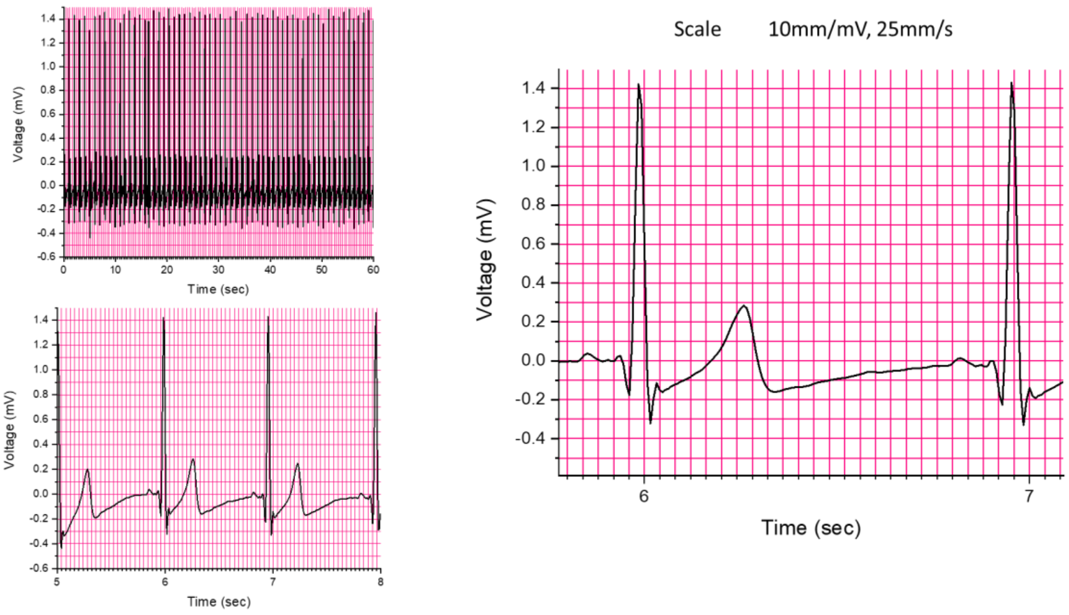

Volunteer 18_Commercial_Single lead ECG system

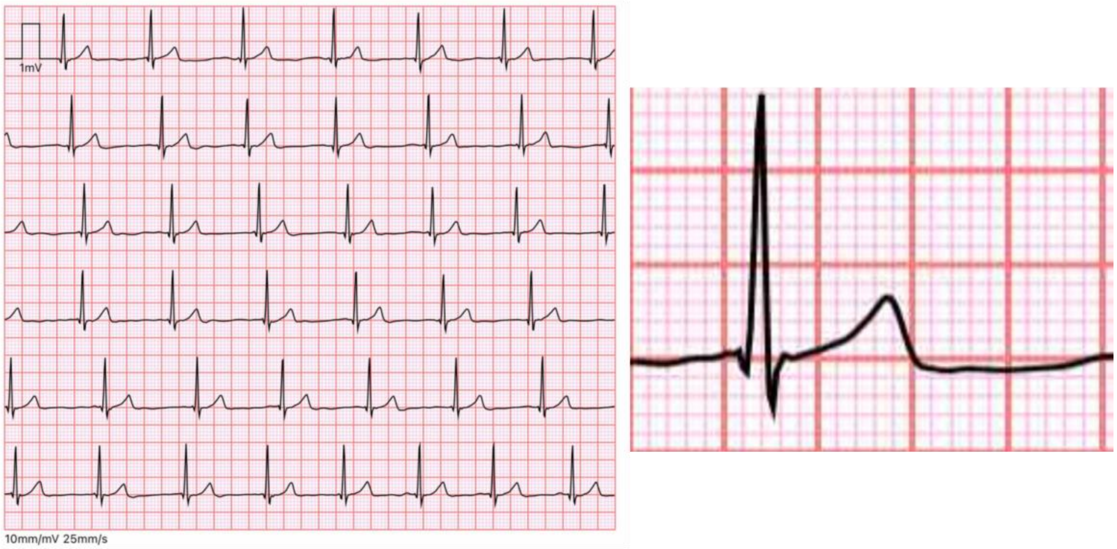

Figure S30a: Volunteer 18, KAUST single lead, and commercial single-lead ECG.

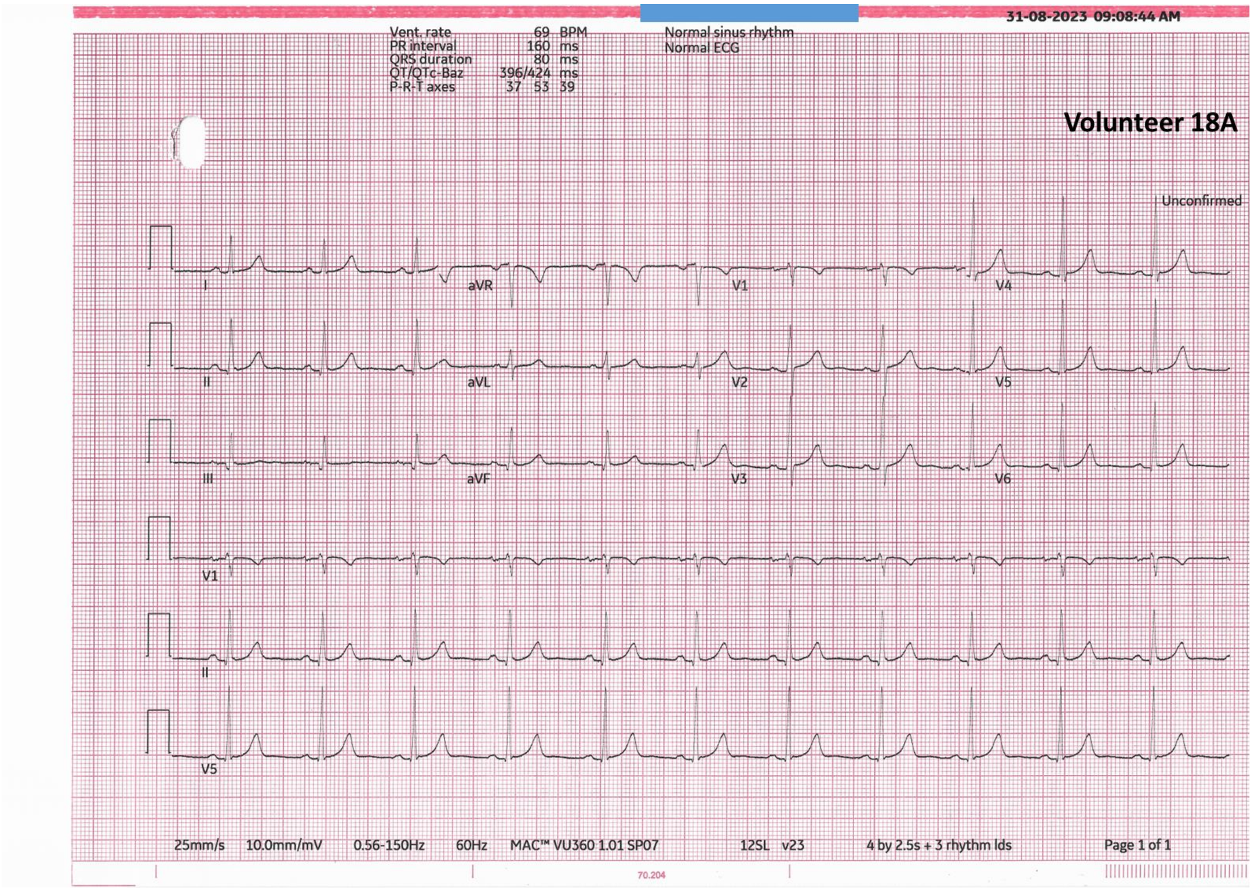

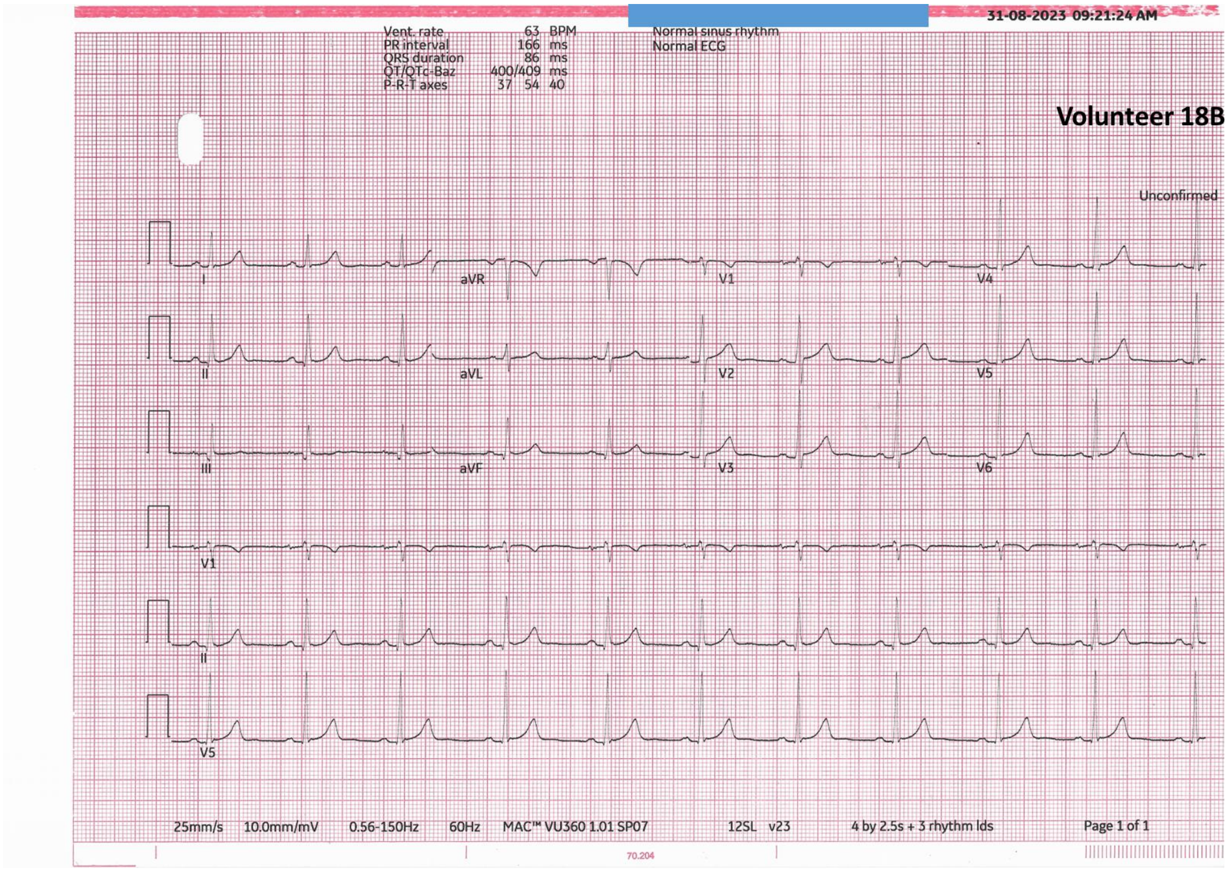

Figure S30b: Volunteer 18: Blinded review of 12 lead ECG using commercial ECG electrodes and gentle-to-skin screen printed ECG electrodes. The sample identification was hidden from the reviewers using a blue color square.

Volunteer No	ECG Type	Leads	Reviewer 1 (Cardiology Consultant)	Reviewer 2 (Cardiology Consultant)	Reviewer 3 (FM Consultant)
V19	A	12	Normal sinus rhythm, normal intervals, normal ST segments	Normal sinus rhythm, normal intervals, normal ST segments	Normal sinus rhythm, normal intervals, normal ST segments
	B	12	Normal sinus rhythm, normal intervals, normal ST segments	Normal sinus rhythm, normal intervals, normal ST segments	Normal sinus rhythm, normal intervals, normal ST segments
	KAUST	1	Normal sinus rhythm, normal intervals, normal ST segments	Normal sinus rhythm, normal intervals, normal ST segments	Normal sinus rhythm, normal intervals, normal ST segments
	Commercial	1	Normal sinus rhythm, normal intervals, normal ST segments	Normal sinus rhythm, normal intervals, normal ST segments	Normal sinus rhythm, normal intervals, normal ST segments
	Summary		All 4 ECGS had similar interpretation	All 4 ECGS had similar interpretation	All 4 ECGS had similar interpretation

Volunteer 19_KAUST_Single lead ECG system

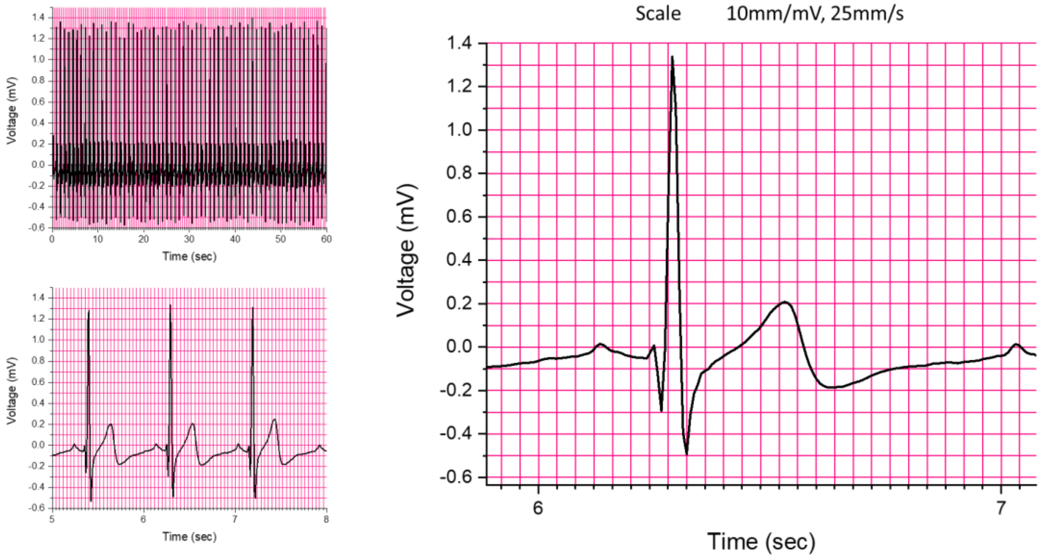

Volunteer 19_Commercial_Single lead ECG system

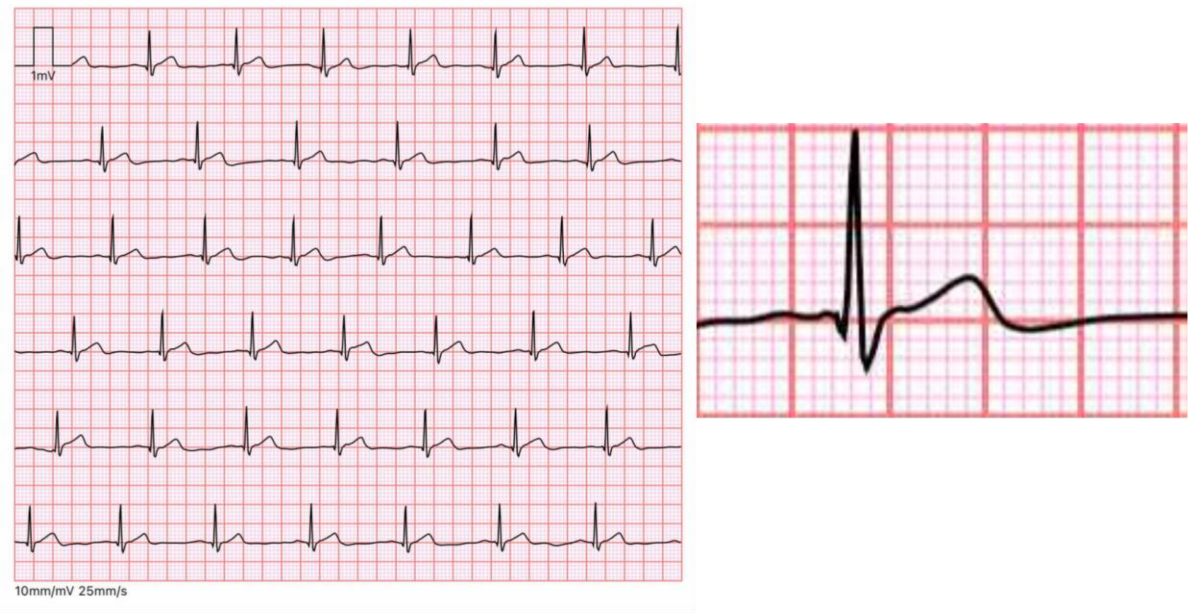

Figure S31a: Volunteer 19, KAUST single lead, and commercial single-lead ECG.

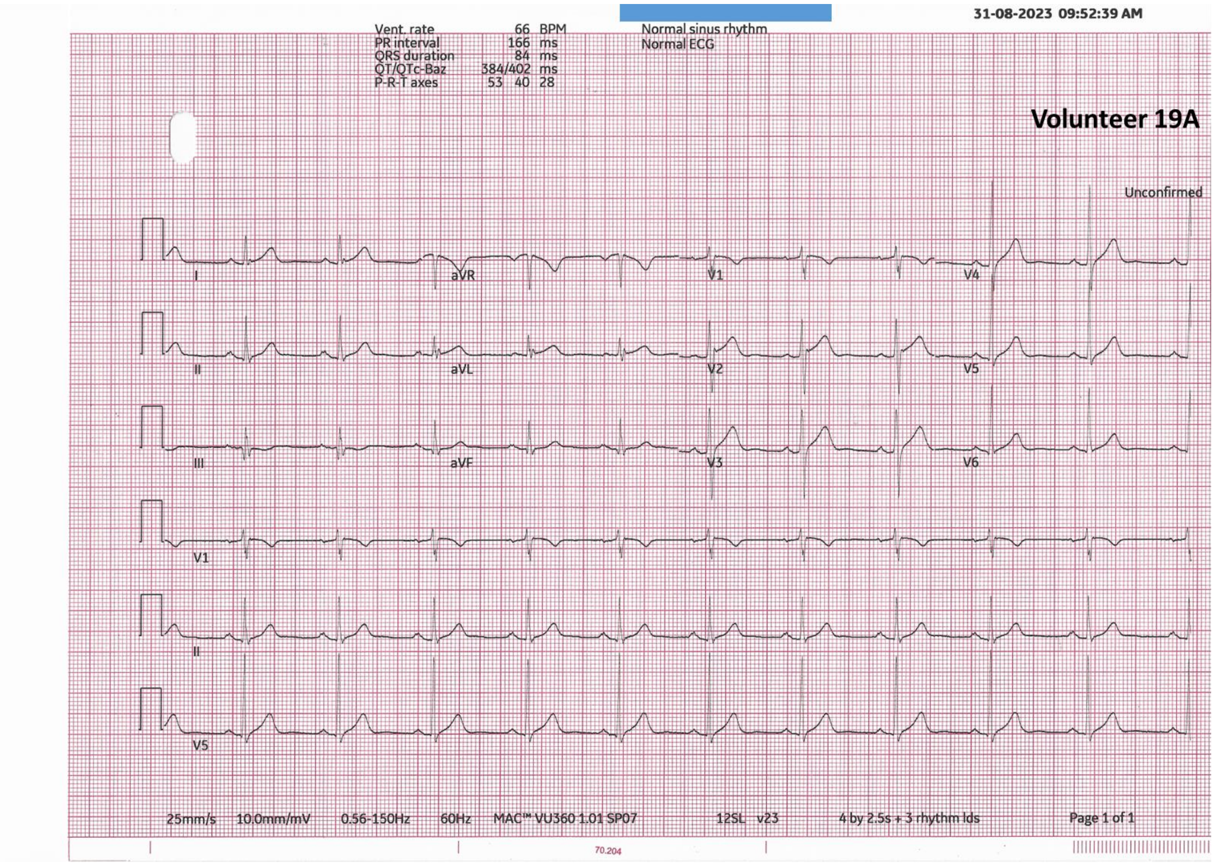

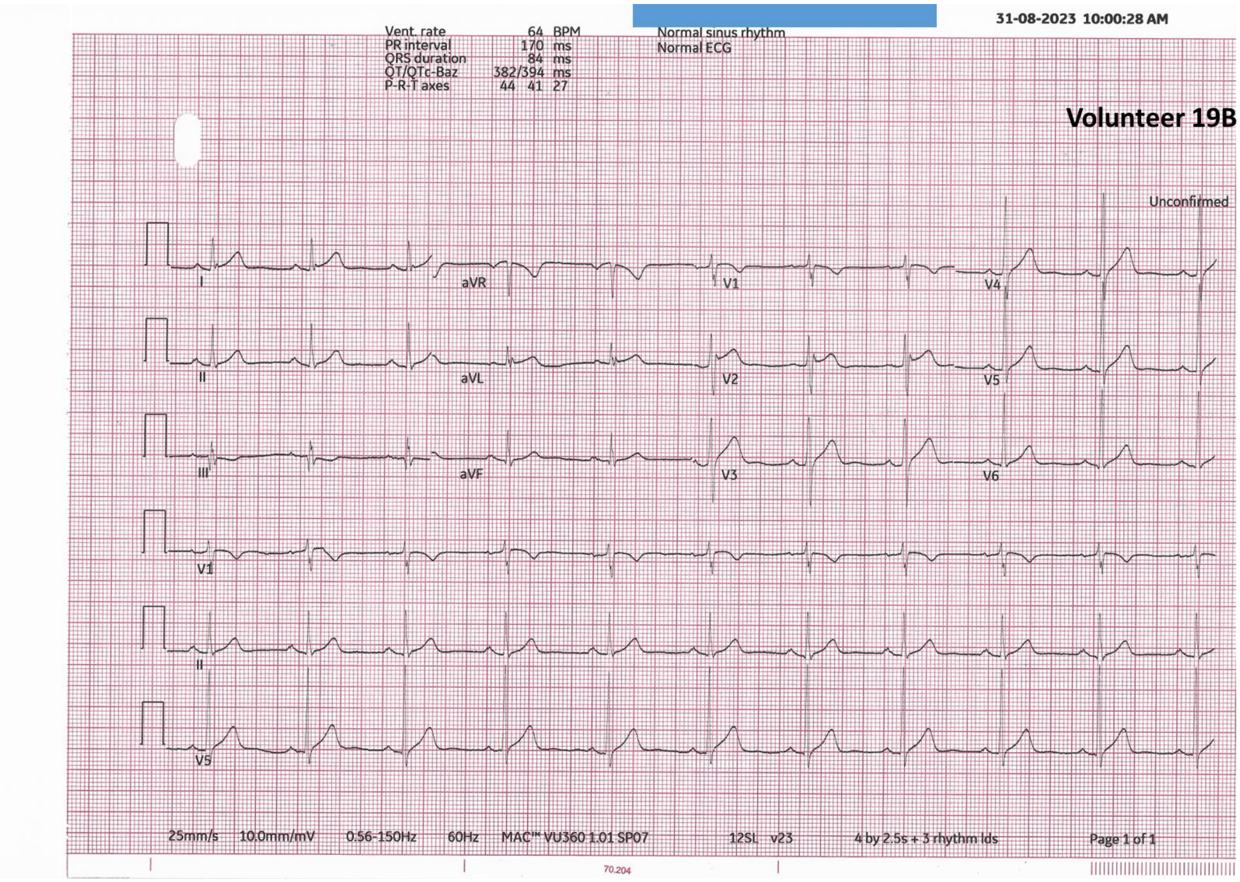

Figure S31b: Volunteer 19: Blinded review of 12 lead ECG using commercial ECG electrodes and gentle-to-skin screen printed ECG electrodes. The sample identification was hidden from the reviewers using a blue color square.

Volunteer No	ECG Type	Leads	Reviewer 1 (Cardiology Consultant)	Reviewer 2 (Cardiology Consultant)	Reviewer 3 (FM Consultant)
V20	A	12	Normal sinus rhythm, normal intervals, normal ST segments	Normal sinus rhythm, normal intervals, normal ST segments	Normal sinus rhythm, normal intervals, normal ST segments
	B	12	Normal sinus rhythm, normal intervals, normal ST segments	Normal sinus rhythm, normal intervals, normal ST segments	Normal sinus rhythm, normal intervals, normal ST segments
	KAUST	1	Normal sinus rhythm, normal intervals, normal ST segments	NSR, T wave inversion, Otherwise, normal intervals and segments	Normal sinus rhythm, normal intervals, normal ST segments
	Commercial	1	Normal sinus rhythm, normal intervals, normal ST segments	NSR, T wave inversion, Otherwise, normal intervals and segments	Normal sinus rhythm, normal intervals, normal ST segments
	Summary		All 4 ECGS had similar interpretation	Both 12 and single lead ECGs had similar interpretation	All 4 ECGS had similar interpretation

Volunteer 20_KAUST_Single lead ECG system

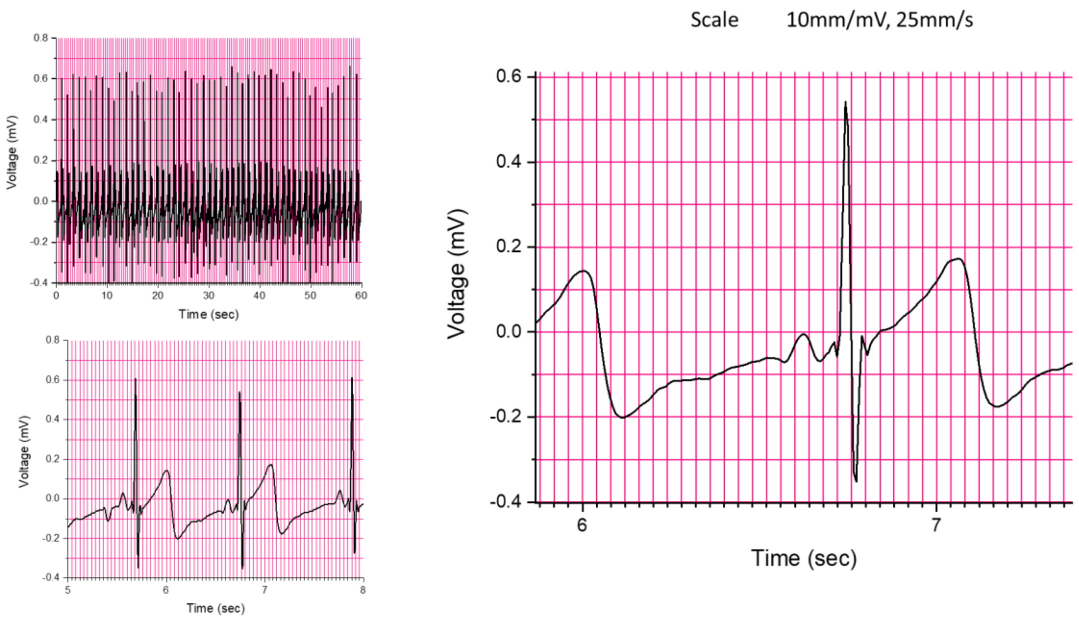

Volunteer 20_Commercial_Single lead ECG system

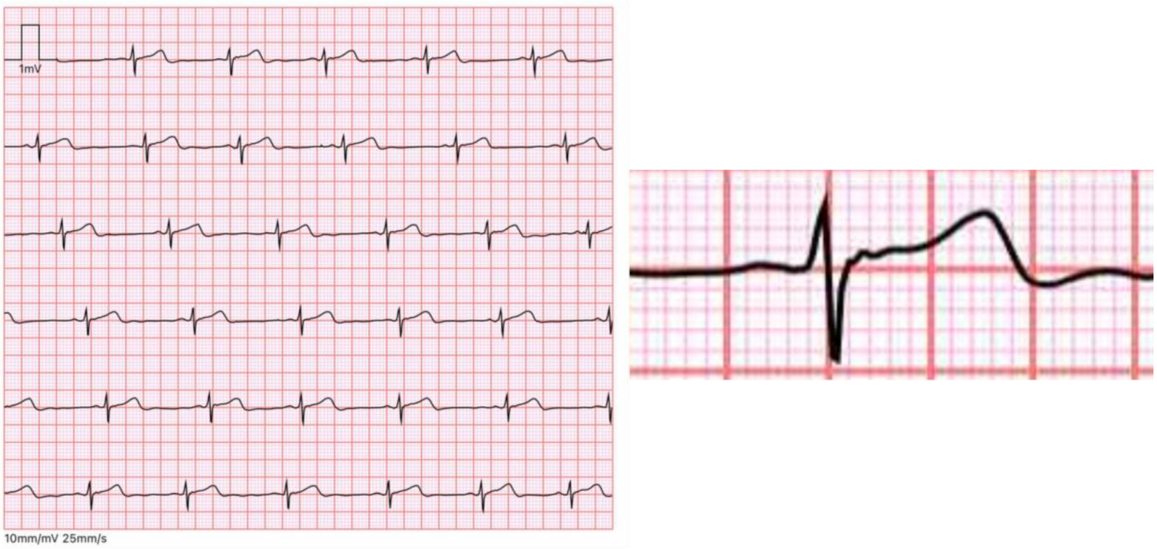

Figure S32a: Volunteer 20, KAUST single lead, and commercial single-lead ECG.

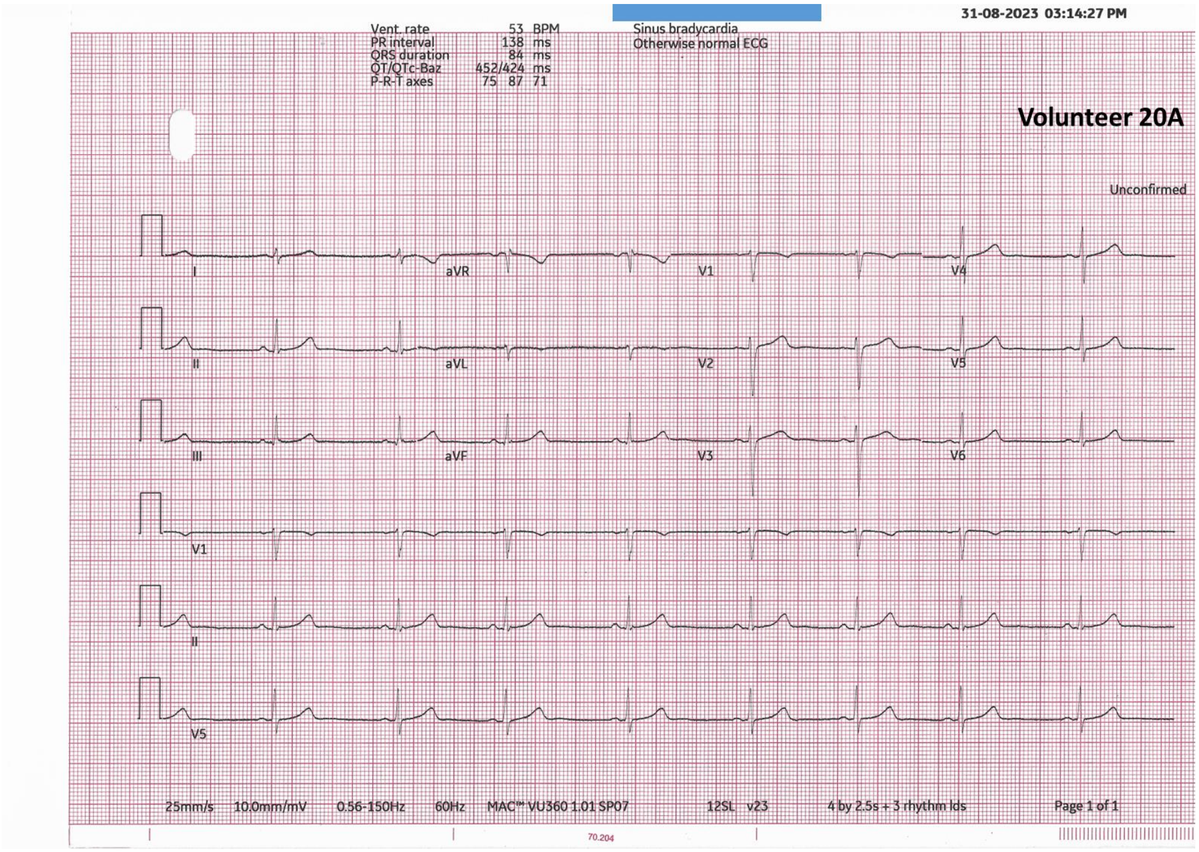

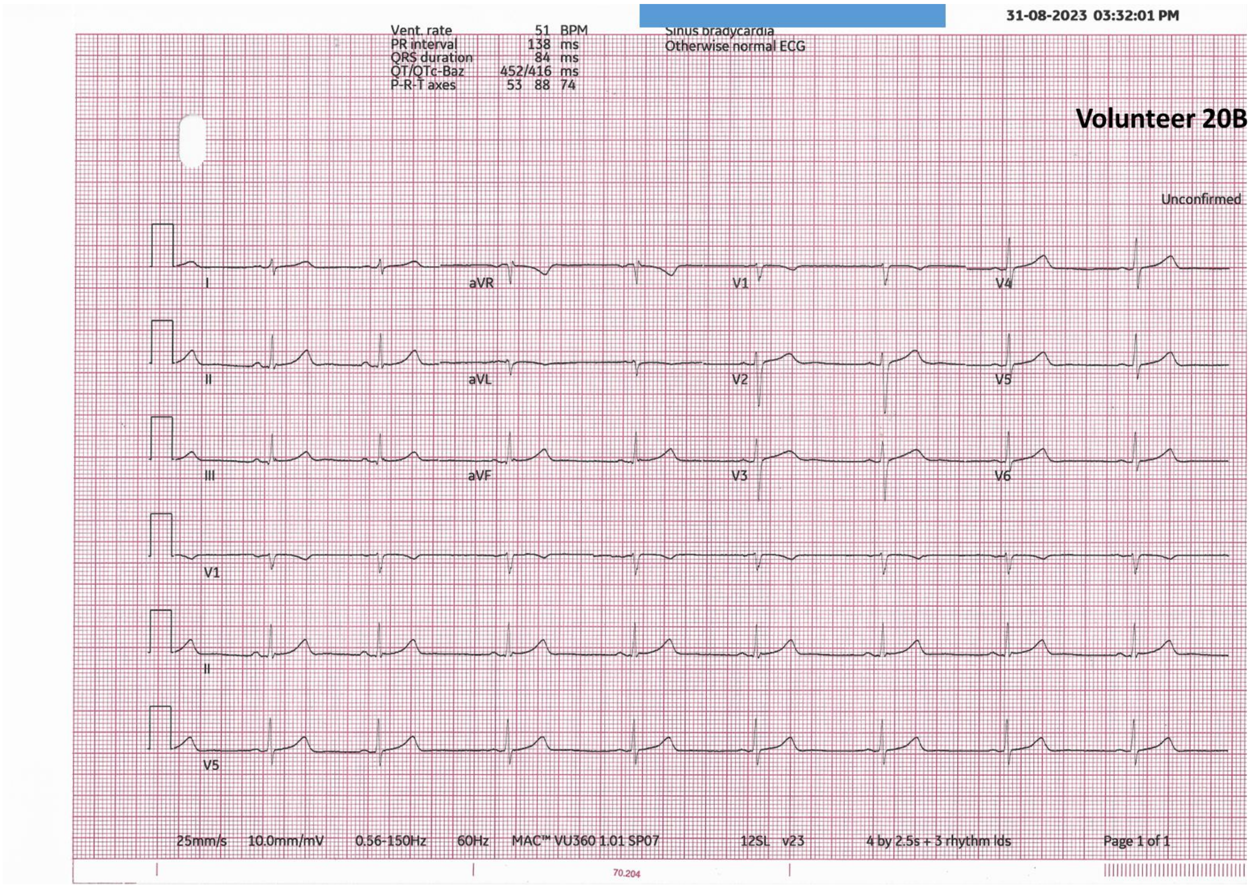

Figure S32b: Volunteer 20: Blinded review of 12 lead ECG using commercial ECG electrodes and gentle-to-skin screen printed ECG electrodes. The sample identification was hidden from the reviewers using a blue color square.

References

(1) Kim, J. H.; Kim, S. R.; Kil, H. J.; Kim, Y. C.; Park, J. W. Highly Conformable, Transparent Electrodes for Epidermal Electronics. *Nano Lett* **2018**, *18* (7), 4531-4540. DOI: 10.1021/acs.nanolett.8b01743.

(2) Abu Zahed, M.; Das, P. S.; Maharjan, P.; Barman, S. C.; Sharifuzzaman, M.; Yoon, S. H.; Park, J. Y. Flexible and robust dry electrodes based on electroconductive polymer spray -coated 3D porous graphene for long-term electrocardiogram signal monitoring system. *Carbon* **2020**, *165*, 26-36.

(3) Liu, L.; Li, H. Y.; Fan, Y. J.; Chen, Y. H.; Kuang, S. Y.; Li, Z. B.; Wang, Z. L.; Zhu, G. Nanofiber-Reinforced Silver Nanowires Network as a Robust, Ultrathin, and Conformable Epidermal Electrode for Ambulatory Monitoring of Physiological Signals. *Small* **2019**, *15* (22). DOI: ARTN 1900755
10.1002/smll.201900755.

(4) Kisannagar, R. R.; Jha, P.; Navalkar, A.; Maji, S. K.; Gupta, D. Fabrication of Silver Nanowire/Polydimethylsiloxane Dry Electrodes by a Vacuum Filtration Method for Electrophysiological Signal Monitoring. *Acs Omega* **2020**, *5* (18), 10260-10265. DOI: 10.1021/acsomega.9b03678.

(5) Kim, T. H.; Bao, C.; Chen, Z. N.; Kim, W. S. 3D printed leech-inspired origami dry electrodes for electrophysiology sensing robots. *Npj Flex Electron* **2022**, *6* (1). DOI: ARTN 5
10.1038/s41528-022-00139-x.

(6) Choi, Y. Y.; Ho, D. H.; Cho, J. H. Self-Healable Hydrogel-Liquid Metal Composite Platform Enabled by a 3D Printed Stamp for a Multimodular Sensor System. *Acs Appl Mater Inter* **2020**, *12* (8), 9824-9832. DOI: 10.1021/acsami.9b22676.

(7) Bihar, E.; Roberts, T.; Saadaoui, M.; Herve, T.; De Graaf, J. B.; Malliaras, G. G. Inkjet-Printed PEDOT:PSS Electrodes on Paper for Electrocardiography. *Adv Healthc Mater* **2017**, *6* (6). DOI: ARTN 1601167
10.1002/adhm.201601167.

(8) Zhang, H. S.; Tian, L.; Lu, H. Y.; Zhou, M.; Zou, H. Q.; Fang, P.; Yao, F.; Li, G. L. A Wearable 12-Lead ECG Acquisition System with Fabric Electrodes. *Ieee Eng Med Bio* **2017**, 4439-4442.

(9) Xu, X. W.; Liu, Z. F.; He, P.; Yang, J. L. Screen printed silver nanowire and graphene oxide hybrid transparent electrodes for long-term electrocardiography monitoring. *J Phys D Appl Phys* **2019**, *52* (45). DOI: ARTN 455401
10.1088/1361-6463/ab3869.

(10) Chansaengsri, K.; Tunhoo, B.; Onlaor, K.; Thiwawong, T. Preparation of Conductive Screen-Printing Ink for High-Performance Bendable and Wearable ECG Electrodes on Fabric Substrates. *Ieee Sens J* **2022**, *22* (24), 23683-23691. DOI: 10.1109/Jsen.2022.3217538.

(11) Tu, H. T.; Li, X. O.; Lin, X. D.; Lang, C. H.; Gao, Y. Washable and Flexible Screen-Printed Ag/AgCl Electrode on Textiles for ECG Monitoring. *Polymers-Basel* **2023**, *15* (18). DOI: ARTN 3665
10.3390/polym15183665.

(12) Sinha, S. K.; Noh, Y.; Reljin, N.; Treich, G. M.; Hajeb-Mohammadalipour, S.; Guo, Y.; Chong, K. H.; Sotzing, G. A. Screen-Printed PEDOT:PSS Electrodes on Commercial Finished Textiles for Electrocardiography. *Acs Appl Mater Inter* **2017**, *9* (43), 37524-37528. DOI: 10.1021/acsami.7b09954.

(13) Xu, X. W.; Luo, M.; He, P.; Guo, X. J.; Yang, J. L. Screen printed graphene electrodes on textile for wearable electrocardiogram monitoring. *Appl Phys a-Mater* **2019**, *125* (10). DOI: ARTN 714
10.1007/s00339-019-3006-x.

(14) Wan, C. X.; Gao, S. H.; Shang, X.; Wu, Z. Y.; Li, T. Y.; Ling, W.; Zhou, M. X.; Huo, W. X.; Guo, Y.; Huang, X. A Flexible and Stretchable 12-Lead Electrocardiogram System with Individually Deformable Interconnects. *Adv Mater Technol-Us* **2022**, *7* (3). DOI: ARTN 2100904
10.1002/admt.202100904.
